# Next-generation epidermal patches: Bridging 3D and multidimensional printing for biomedical and personal care innovations

**DOI:** 10.1016/j.bioactmat.2026.03.054

**Published:** 2026-04-08

**Authors:** Labiba K. El-Khordagui, Salma E. El-Habashy, Abdolreza Simchi, Hebat-Allah S. Tohamy, Maria Letizia Focarete, Mariangela Rea, Luana Di Lisa, Snigdha Roy Barman, Amit Nain, Ovidio Catanzano, Joshua Boateng, Jagan Mohan Dodda

**Affiliations:** a Department of Pharmaceutics, Faculty of Pharmacy, Alexandria University, Egypt; b Fraunhofer Institute for Manufacturing Technology and Advanced Materials (IFAM), 28359 Bremen, Germany; c Cellulose and Paper Department, National Research Centre, 33 El Bohouth Str., P.O. 12622, Dokki Giza, Egypt; d Department of Chemistry “Giacomo Ciamician” University of Bologna, via Gobetti 85, 40129, Bologna, Italy; e Department of Biotechnology, Indian Institute of Technology Madras, Chennai 600036, Tamil Nadu, India; f Department of Applied Mechanics & Biomedical Engineering, Indian Institute of Technology Madras, Chennai 600036, Tamil Nadu, India; g Institute for Polymers, Composites and Biomaterials (IPCB-CNR), Via Campi Flegrei 34, 80078 Pozzuoli, Naples, Italy; h Faculty of Engineering and Science, University of Greenwich, Medway Campus, UK; i New Technologies – Research Centre (NTC), University of West Bohemia, Univerzitní 8, 301 00 Pilsen, Czech Republic

**Keywords:** 3D/4D/5D printing, Microneedles, Bioprinting, Drug delivery, Wound healing, Flexible sensors, Smart aesthetics

## Abstract

Advances in additive manufacturing, particularly 3D and multidimensional printing, have enabled unprecedented control over the architecture, composition, and bioactivity of epidermal patches. These developments have broadened the scope of epidermal patches across biomedical and personal-care applications, supporting personalized and adaptive solutions for drug delivery, wound management, tissue regeneration, and skin-related interventions. This review summarizes next-generation printed epidermal patches, covering both conventional (non-microneedle) systems and microneedle-integrated platforms. Particular emphasis is placed on emerging material systems, including self-oxygenating hydrogels, nanomaterial-free bioinks derived from proteins and polysaccharides, and functional nanocomposite formulations. We examine key 3D printing strategies for fabricating acellular constructs, cell-laden matrices, and microneedle array patches (MAPs), alongside recent advances in multidimensional printing technologies. Biomedical applications are discussed with a focus on dermal and transdermal drug delivery, particularly insulin delivery for diabetes management as well as wound repair, regenerative therapies, photodynamic treatments, and biosensing. Additionally, the integration of printed epidermal patches with wearable sensors, smart devices, and artificial intelligence (AI) is highlighted as an emerging frontier in intelligent skin-interfaced systems, with implications for both healthcare and advanced personal-care technologies. Finally, key challenges related to clinical translation, regulatory pathways, and commercialization are addressed, providing strategic insights to guide the advancement of hydrogel-based additive manufacturing from laboratory innovation to real-world clinical and aesthetic applications.

## Highlights

• 3D printing enables next-generation epidermal patches using bioinks, acellular matrices, cell-laden constructs, and microneedles.
• 4D and 5D printing advances smart epidermal patches with expanded functional versatility.
• Printed patches and microneedles support drug delivery, wound healing, skin regeneration, photodynamic therapy, and biosensing.
• Integration of printed patches with sensors and AI drives innovation in personalized cosmetics.
• Clinical development, regulatory hurdles, and commercialization shape translation to market.

## Graphical abstract

**Figure:**
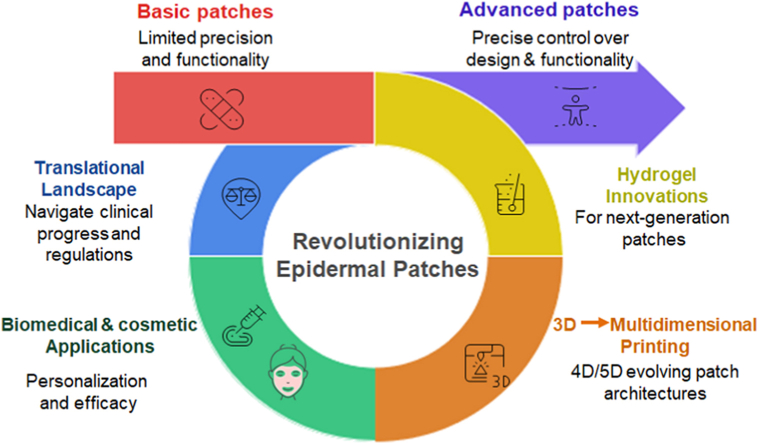

## 1 Introduction

Epidermal patches have long functioned as skin-interface platforms, offering a non-invasive, patient-friendly means of localized drug delivery via diffusion, while also supporting wound healing and skin regeneration [1]. These patches have undergone significant advancements, driven by progress in materials science, fabrication technologies, and a deeper understanding of skin biology, thereby improving their effectiveness across a wide range of biomedical and aesthetic applications. Initially, epidermal patches were designed as adhesive films, primarily functioning as matrices for dermal and transdermal drug delivery by employing polymers such as ethylene-vinyl acetate and silicone to facilitate drug diffusion through the skin [2,3]. Although effective for specific applications, their reliance on passive drug diffusion limited their ability to achieve controlled or targeted drug release. Subsequently, functionalized polymeric and hydrogel-based patches incorporating bioactive agents were developed to enhance therapeutic efficacy [1]. In this context, natural polymers such as chitosan, alginate, and hyaluronic acid have been widely utilized to create patches that not only enable drug delivery but also promote wound healing and skin regeneration [4,5]. The biological properties of natural polymers, such as biocompatibility, biodegradability, and reactivity to different stimuli, enhance their suitability for the development of drug delivery systems that provide support for enhanced therapeutic efficacy with minimized harmful effects [6]. These advancements have significantly contributed to the growth of the tissue engineering field in recent decades, leading to the development of skin substitutes that mimic the structure and function of native tissue [7]. By promoting the healing of chronic wounds and skin regeneration, these skin patches provide an alternative to traditional donor skin grafts [8]. While skin grafts remain the gold standard in skin tissue engineering, they are limited by donor availability and complications such as immune rejection, infection, and scarring [9].

In recent decades, electrospinning technologies have facilitated the fabrication of nanofibrous epidermal patches [10,11], characterized by high surface area and porosity—key features that enhance cell adhesion and proliferation. These patches also act as versatile delivery platforms capable of incorporating drugs, growth factors, nanoparticles, and living cells, supporting various applications in drug delivery and regenerative medicine [12]. Advancements in nanotechnology have also led to the development of nanoengineered, stimuli-responsive patches. These smart materials, capable of responding dynamically to diverse physical, chemical, and biological stimuli, offer innovative solutions to longstanding challenges in tissue engineering, drug delivery, and wound healing [13].

Representing a major innovation, microneedle array patches (MAPs) have emerged as a transformative, minimally invasive platform of epidermal patches, revolutionizing biomedical applications, particularly transdermal drug delivery, tissue regeneration, and wound healing [14,15]. Microneedles (MNs) offer a minimally invasive alternative to conventional hypodermic injections, designed to painlessly penetrate the stratum corneum without reaching nerve-rich deeper tissues. Their ability to deliver therapeutics, ranging from small molecules and vaccines to nanoparticles (NPs) and living cells, directly into the skin has broadened their clinical potential [16,17]. MNs enable precise transdermal drug delivery by creating microchannels in the stratum corneum, allowing localized administration, improved bioavailability, and enhanced skin penetration without contacting nerve endings or blood vessels [18].

Notably, the most recent phase in the evolution of epidermal patches has marked the emergence of 3D printing or additive manufacturing, a cutting-edge technology based on the x, y, and z axes that enables personalized solutions across a wide range of biomedical applications [19,20]. This technique allows for the layer-by-layer fabrication of structures directly from digital models, offering exceptional control over structural complexity and spatial resolution that were previously unattainable using conventional manufacturing methods. Moreover, 3D bioprinting of tissues and organs, using bioinks composed of living cells and biomaterials, is advancing rapidly, supported by growing research in bioinks, printing technologies, and applications such as drug testing and personalized implants [21]. In parallel, 3D printing is emerging as a transformative innovation in the cosmetics and personal care field, revolutionizing both product development and personalized skincare solutions. Applications range from customized makeup and skincare patches to bioprinted skin models for safety and efficacy testing, introducing a new level of precision, personalization, and sustainability [[22], [23], [24]].

Although 3D printing offers significant structural customization, it faces several technical, material, and regulatory limitations that hinder its broader adoption in advanced biomedical and cosmetic innovations. These mainly include restricted material diversity, limited functional integration, such as the inability to simultaneously incorporate sensing, actuation, or controlled drug release, and the production of constructs that are typically static in form and function after fabrication. Such constraints have driven the evolution toward multidimensional printing as an advanced extension of 3D printing. Multidimensional printing introduces dynamic capabilities through additional dimensions, such as time in 4D printing [25] and multi-axis fabrication in 5D printing [26], thereby enabling the development of responsive and functionally adaptive epidermal patches. Engineered for biomedical precision, multidimensional printed patches enable multi-material and advanced predictive, preventive, and personalized medicine by facilitating diagnostic sensing, supporting customized drug delivery, and promoting wound healing and tissue regenerative functions [[27], [28], [29], [30], [31]].

Moreover, multidimensional printing technologies have positioned bioprinting as a transformative approach that represents a significant advancement in regenerative medicine [32,33]. By integrating a diverse range of biomaterials, varying in composition and mechanical properties, alongside additional dimensions into the printing process, the limitations of single-material systems and the uniformity of fixed-dimension methods are effectively overcome. This advancement enables dynamic structural transformations and responsive functionalities. In addition to biomedical applications, multidimensional printing technologies are increasingly being investigated in the realm of personalized care, showing significant potential for cosmetotextiles. These innovations often incorporate shape-memory polymers (SMPs), enabling materials to dynamically respond to individual skin needs [34].

This review presents a comprehensive integration of advances in 3D and multidimensional printing of epidermal patches, encompassing both structurally conventional (non-microneedle) and microneedle array formats. It examines advanced materials and state-of-the-art fabrication technologies, with a particular focus on their roles in driving progress in biomedical and cosmetic applications. The review also addresses key challenges that currently impede the clinical and commercial translation of these technologies. By adopting a multidisciplinary perspective, it highlights the potential of advanced 3D/4D/5D-printed epidermal patches as next-generation platforms for personalized medicines, non-invasive therapeutic interventions, and aesthetic enhancement.

## 2 Materials for 3D printed epidermal patches

### 2.1 Required material properties

Hydrogels suitable for 3D printing must meet specific rheological, mechanical, and biological criteria. Among their physicochemical features, rheological properties are particularly impactful for determining printability [35]. Within the broader context of hydrogel rheology, several parameters, including viscosity (η), storage modulus (G′), loss modulus (G″), yield stress (τ), and recovery capabilities, have been linked to the final printing outcomes. The solution viscosity and its resistance strongly influence filament formation and spatial resolution. To achieve optimal flow and shape retention, bioinks must exhibit viscoelastic behavior, a combination of elastic deformation (shape retention after printing) and viscous flow (ability to flow through the nozzle) [36]. Shear-thinning indicates the non-Newtonian behavior in which the viscosity decreases as the shear rate increases, as a consequence of shear-induced reorganization of the polymer chains to a stretched conformation, which leads to decreased entanglement. The shear-thinning behavior of bioinks is typically quantified through flow curve tests, plotting viscosity (η) as a function of applied shear rate ($\dot{\gamma }$). Quantitatively, the power-law model (η = K $\dot{\gamma }$^n−1^) describes this behavior, where low flow index (*n*) values correspond to strong shear thinning [37]. Printable hydrogels generally exhibit a marked decrease in viscosity with increasing shear rate, enabling lower-pressure extrusion while preserving filament geometry post-extrusion. While sufficient viscosity is needed for precise printing, excessive viscosity can negatively impact the cell viability and the overall quality of the printed construct [[38], [39], [40]]. Therefore, it is advantageous to utilize hydrogels with low concentrations but high viscosities for smooth extrusion through the printer nozzle [35]. Another crucial factor is the transition from fluid-like behavior during extrusion to solid-like form retention after deposition [41]. This property depends on the ability of the hydrogel network to recover its viscosity or shear modulus after being disrupted by stress that exceed the yield point. [42,43]. During extrusion, the hydrogel ink experiences shear stress, particularly at the nozzle wall, which is influenced by many parameters such as pressure, nozzle diameter, viscosity, and the embedded cells [44,45]. It has been demonstrated that short-term exposure to high shear stress can significantly impact cell viability within cell-laden hydrogels. For instance, *in vivo* studies on mice have shown that the survival rate decreases from 91% to 76% as shear stress increases from 5 to 10 kPa to over 10 kPa, respectively [46]. It has also been demonstrated that cell viability reached up to 96% at lower stress levels.

After extrusion, the physically cross-linked network of hydrogel, disrupted by shear stress, should be able to self-recover. Therefore, thixotropy, defined as the progressive decrease in viscosity of a material with time under applied shear stress, followed by a gradual recovery when the stress is removed, is a crucial characteristic for a suitable extrusion-based bioprinting hydrogel. Thixotropic materials include thixotropic paints and silk nanofibril-based hydrogels [47]. On the other hand, the shear-thinning behavior of bioinks facilitates their easy extrusion while enabling them to retain their original shape post-deposition [48,49]. Slow network recovery may necessitate reduced printing speeds or increased filament spacing to prevent structural collapse. Gelation kinetics of hydrogels is another critical factor influencing print resolution, mechanical stability, and cell viability [50]. The rate at which a liquid hydrogel transitions to a solid gel determines the success of layer-by-layer deposition [50,51]. Rapid gelation could cause clogging or solidification at the nozzle, while slow gelation could result in filament spreading and loss of structural precision. Moreover, it safeguards embedded cells from potential damage during the rapid transition from liquid to solid [51]. Researchers employ various techniques to modulate gelation kinetics, such as temperature control [[52], [53], [54]], pH adjustment [52,55,56], light-induced gelation [57,58], and chemical crosslinking [59]. By carefully tailoring these parameters, they can achieve the desired gelation rates, enabling the creation of 3D-printed hydrogels with specific properties and applications [51,[60], [61], [62]]. Finally, the degradation rate is another key factor that influences the suitability of printed structures for specific biomedical applications. The hydrogel constructs should be able to maintain stability (*in vitro* or *in vivo*) for a particular duration, depending on their intended use [63]. Therefore, the bioink should have a suitable degradation rate to maintain structural integrity until tissue regeneration is nearly complete.

Beyond rheological properties, there are other crucial parameters that play a significant role in determining the performance and functionality of hydrogels in 3D printing and bioprinting applications. The mechanical properties of the printed structures, including their integrity and durability, are essential for maintaining structural stability and functionality over time and successful integration within biological systems [64]. Integrity refers to the hydrogel's ability to maintain its intended shape and structural coherence under various stresses, including compression, tension, and shear [65,66]. This is particularly crucial for applications involving load-bearing tissues like bone or cartilage, where the hydrogel must withstand physiological forces without deformation or fracture [65]. Conversely, durability signifies the hydrogel's resistance to degradation and fatigue over extended periods. This is vital for applications requiring long-term implantation or sustained release of therapeutic agents [67]. The mechanical properties are intimately linked to the hydrogel's crosslinking density, polymer composition, and architecture. For example, higher crosslinking densities generally enhance stiffness and compressive strength but can also reduce flexibility [68]. Matching the mechanical properties of the printed hydrogel to those of the target tissue is essential for promoting cell adhesion, proliferation, and differentiation. Mismatched mechanical properties can lead to stress shielding, tissue damage, or implant failure [69,70]. Therefore, meticulous control over the mechanical properties of the hydrogel is indispensable for creating functional and durable 3D-printed constructs for diverse biomedical applications.

Biocompatibility and hemocompatibility are essential, particularly for applications involving tissue regeneration and blood contact, ensuring that the hydrogel does not elicit adverse biological responses [71]. Biocompatibility, in essence, refers to the ability of the material to interact with biological systems without causing harmful effects. This extends beyond simple non-toxicity; it encompasses the material's capacity to support cell adhesion, proliferation, and differentiation, encouraging tissue integration and remodeling. In tissue regeneration, a biocompatible hydrogel provides a conducive microenvironment for cells to thrive, facilitating functional tissue formation [71]. Insufficient biocompatibility may trigger undesired immune activation, as the body recognizes it as foreign, leading to inflammatory responses, macrophage-driven foreign body reactions, and the formation of fibrotic capsules [72]. Hemocompatibility, a critical aspect for blood-contacting applications such as vascular grafts or heart valve replacements, focuses on the material's interaction with blood components [73]. A hemocompatible hydrogel must prevent thrombosis, the formation of blood clots, and minimize damage to blood cells, such as hemolysis. This requires careful selection of materials and surface modifications that discourage protein adsorption and platelet activation [73,74]. Failure to achieve adequate biocompatibility or hemocompatibility can lead to inflammation, rejection, or life-threatening complications. Therefore, rigorous *in vitro* and *in vivo* assays are essential to validate the safety and efficacy of 3D-printed hydrogels for biomedical applications. Furthermore, the potential toxicity of byproducts released during hydrogel degradation or crosslinking must be carefully evaluated to guarantee safety [75]. Hydrogel degradation generally proceeds through hydrolytic or enzymatic mechanisms, generating soluble fragments, such as monomers, crosslinkers, or intermediate species, that may induce cytotoxic or immunogenic effects [75,76]. Crosslinking typically improves hydrogel stability but also enhances resistance to degradation. In particular, the ester hydrolysis rate constant is higher for crosslinked hydrogels compared to non-crosslinked hydrogels [77]. The rate of hydrogel degradation *in vivo* critically shapes immune responses: rapid degradation can induce acute activation, promoting antigen presentation, and stimulating immune cell infiltration [78], whereas slow-degrading systems provide sustained modulation but may increase the risk of prolonged foreign-body responses [79]. Thorough *in vitro* and *in vivo* toxicity assessments are essential to ensure safety. These assessments should evaluate the effects of released byproducts on cell viability, tissue inflammation, and systemic toxicity. Techniques such as cytotoxicity assays, hemolysis tests, and histological analyses are commonly employed to evaluate these risks [80]. The degradation rate and byproduct release profile should also be carefully controlled during hydrogel design and fabrication to minimize potential adverse effects [81]. Natural and synthetic hydrogels exhibit distinct immunological profiles. Biopolymers such as hyaluronic acid (HA) and gelatin offer excellent biomimetic behavior and are efficiently degraded by endogenous enzymes, supporting matrix remodeling. However, their immunogenicity may vary depending on the nature of degradation fragments [72]. In contrast, synthetic materials such as PEG or PVA offer tunable and highly reproducible properties, enabling more controlled immune interactions; however, formulations with limited degradability may persist in tissues and induce low-grade inflammation or fibrotic encapsulation [82]. Upon implantation, both natural and synthetic hydrogels trigger a cascade of host responses that influence long-term stability and degradation [83]. This process begins with an acute inflammatory phase characterized by M1 macrophage activation and the secretion of pro-inflammatory cytokines, such as IL-1α, IL-1β, TNF-α, and IL-6. As inflammation progresses, a transition toward an M2 macrophage–dominated environment promotes tissue remodeling and contributes to enzymatic degradation through IL-10 and other pro-resolving mediators [84]. However, if this transition is incomplete, chronic inflammation, granulation tissue formation, and fibrous encapsulation may occur, influencing hydrogel integration, degradation kinetics, and cell-material interactions. For example, Zhang L. et al. [85] obtained two chemically modified HA-based hydrogels by methacrylic (MAHA) and maleic (MEHA) functionalization synthesis. They deeply studied the biocompatibility and immunomodulatory behavior of both synthesized materials using *in vitro* and *in vivo* models. The findings indicated that both MAHA and MEHA promote cell proliferation and exhibit anti-inflammatory properties, as reflected by the elevated IL-10 levels (57.92 ± 9.87 pg mL^−1^ for MEHA and 68.08 ± 13.94 pg mL^−1^ for MAHA). Subcutaneous implantation in BALB/c mice for 28 days confirmed the absence of chronic inflammation for either material.

A comprehensive assessment of hydrogel properties, encompassing rheology, mechanical strength, biocompatibility, hemocompatibility, and toxicity, is critical for successfully developing and translating 3D printed hydrogel constructs into effective and safe biomedical applications. The relationship between material properties and biological performance is mediated by the way hydrogels transmit biochemical and mechanical cues to cells [86]. Rheological characteristics are essential not only for ensuring smooth extrusion during 3D printing but also for preserving cell viability by minimizing shear-induced damage. It is well known that crosslinking density and network architecture define the stiffness and elasticity of the printed scaffold, which in turn influence cell behavior through mechano-transduction [87]. A higher crosslinking density improves mechanical strength but may limit cell spreading and nutrient diffusion, while moderate stiffness promotes adhesion, proliferation, and lineage-specific differentiation. These mechanical cues activate intracellular pathways that regulate cytoskeletal organization and gene expression. Hydrogel properties are therefore responsible for establishing a dynamic and instructive microenvironment that directs cellular responses, ensuring the success of 3D-printed constructs.

Sustainability has become a fundamental design requirement for epidermal patches due to the growing environmental impact associated with disposable medical devices, many of which generate significant plastic and electronic waste. 3D printing offers unique advantages in addressing these challenges, as it reduces material waste, improves energy efficiency, and integrates advanced digital innovations, demonstrating how modern manufacturing technologies can support broader sustainability objectives in biomedical device fabrication [88]. By offering precise control over material deposition and device architecture, additive manufacturing inherently reduces material waste and supports resource-efficient, on-demand production, enabling more sustainable and environmentally responsible manufacturing workflows [88].

However, the sustainability of 3D printing must also be evaluated critically, as many additive manufacturing processes are energy-intensive. In fact, the environmental footprint of most 3D printers is driven more by electricity consumption than by material choice, particularly in systems that rely on prolonged heating, laser exposure, or high-temperature extrusion. Recent analyses further indicate that the electricity powering these systems is still predominantly generated from fossil-fuel–based power stations, amplifying their overall environmental impact. Nevertheless, additive manufacturing can support more resource-efficient production when properly optimized. For example, machine utilization can be improved by minimizing idle time between prints and reducing the operation of unproductive systems, thereby avoiding unnecessary energy consumption and improving the overall sustainability of the manufacturing workflow. Material selection also contributes to the environmental profile of 3D printing [89,90]. For instance, PLA requires less energy during processing due to its lower melting point compared to ABS, making it inherently less energy-demanding and generally more environmentally favorable. Likewise, the adoption of biobased and biodegradable polymers can further reduce the ecological impact of printed biomedical devices [91]. Moreover, AI-driven approaches are increasingly enhancing the sustainability of additive manufacturing. AI's capabilities in predicting material performance, optimizing bioink formulations, dynamically adjusting printing parameters, and supporting intelligent bioprinting systems substantially reduce reliance on resource-intensive and wasteful trial-and-error experimentation, leading to significant improvements in efficiency and overall environmental performance [92]. 3D printing's ability to decentralize production also shifts supply chains toward localized and digital models, reducing transportation-related emissions and supporting circular-economy frameworks. Unlike injection molding, which requires costly molds and tooling, additive manufacturing involves relatively low fixed costs, making it economically viable for small production runs (e.g., printing of point-of-care formulations for hospital patients at the point of use), personalized devices, and niche biomedical markets [88].

### 2.2 Innovative materials

#### 2.2.1 Self-oxygenating hydrogels

**Fig. 1:**
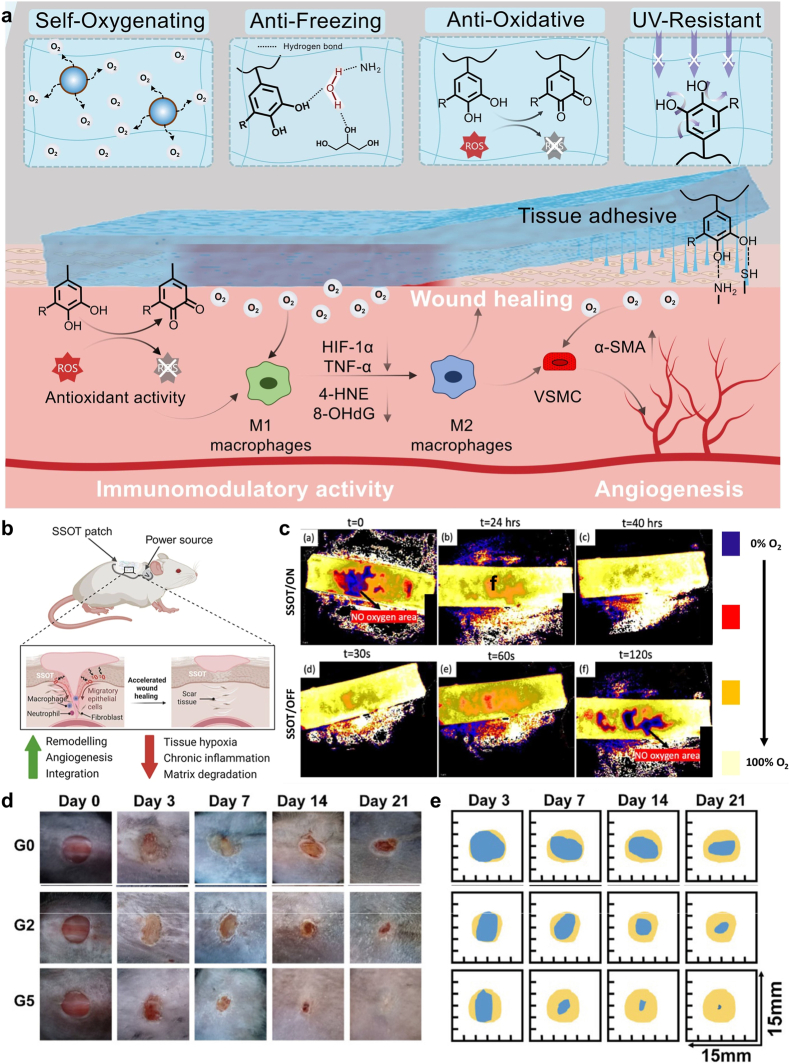
**Selected self-oxygenating hydrogel strategies: (a) Peroxide-based systems:** A multifunctional hydrogel composed of a glycerol/water matrix incorporating dopamine-modified calcium peroxide nanoparticles (PCaO_2_ NPs), demethoxylated lignin (DL), and polyacrylamide (PAM) [107]. The hydrogel exhibits adhesive, self-oxygenating, anti-freezing, antioxidant, and UV-resistant properties, promoting wound healing under high-altitude conditions via enhanced immunomodulation and angiogenesis. Adapted with permission from Ref. [107]. Copyright 2024, Elsevier. **(b**–**c) Power source-based smart self-oxygenating tissue (SSOT) platform:** A bio-ionic liquid (BIL)-functionalized hydrogel electrolyte enables localized, controlled oxygen generation (b). Oxygen evolution over time is shown under intermittent voltage application (c). Data are presented as means ± SEM (n = 4); statistical significance was determined by one-way ANOVA with Tukey's multiple comparisons (ns = not significant, ∗P < 0.05, ∗∗P < 0.01, ∗∗∗P < 0.001, ∗∗∗∗P < 0.0001). Reproduced with permission from Ref. [109]. Copyright 2026, Nature. (d–e) Cyanobacteria-based oxygen generation strategy: A double-layered hydrogel with an inner oxidized sodium alginate/CMCS layer containing a photodynamic MOF (PCN-224) and pH indicator bromothymol blue, and an outer CMCS layer loaded with cyanobacteria for diabetic wound healing: (d) Representative wound images for different treatments (G0: PBS; G2: Gel1(Cyan); G5: Gel1(Cyan)/Gel2(PCN)) on days 0, 3, 7, 14, and 21 post-operation in mice and (e) Corresponding wound healing simulation analysis. Data are shown as mean ± SD; significance assessed by one-way ANOVA (∗P < 0.05, ∗∗P < 0.01, ∗∗∗P < 0.001). Reproduced with permission from Ref. [110]. Copyright 2022, Wiley-VCH GmbH.

Researchers have explored various strategies to develop self-oxygenating hydrogels capable of autonomously generating and supplying oxygen to the surrounding cells. These hydrogels offer a promising solution to hypoxia in tissue constructs, facilitating the survival of the engineered tissue [93]. These self-oxygenating hydrogels are designed to produce oxygen via two mechanisms, falling into two major categories of oxygen generation and oxygen storage/release [94,95]. The oxygen generation mechanism is based on hydrogels' direct generation of oxygen to meet the oxygen requirement in the tissues. Typically, hydrogels are embedded with peroxides, which generate oxygen by chemical decomposition. The peroxides react with water to produce intermediate products such as hydroxides and hydrogen peroxide (H_2_O_2_) which dissociate into water and oxygen [95,96]. Compared to liquid peroxides, solid peroxides offer gradual oxygen release and significantly higher oxygen generation owing to their slower decomposition rate and comparatively lower water solubility [97]. To this end, solid peroxides, such as calcium peroxide (CPO), sodium percarbonate (SPC), and magnesium peroxide (MPO), have been incorporated into hydrogels for the preparation of self-oxygenating hydrogels [98,99]. CPO (CaO_2_) has emerged as a promising oxygen-generating material due to its low solubility coefficient, high purity, and good biocompatibility [[100], [101], [102]]. The material provides sustainable and prolonged release of O_2_ owing to its two-step decomposition process in the presence of water, which leads to the release of O_2_ along with byproducts, as per the equation: 2CaO_2_+2H_2_O→2Ca(OH)_2_+O_2_ [103,104]. Nonetheless, a catalyst-such as catalase, may be incorporated into the hydrogel matrix to enable spontaneous conversions, accelerating the O_2_ generation process [105]. Various studies have shown that the rate of O_2_ generation from hydrogels could be controlled by regulating the form and concentration of peroxides or enzymes incorporated within the scaffolds [97,106]. Xie et al. [107] developed a multifunctional wound-healing hydrogel by integrating dopamine-modified calcium peroxide nanoparticles (PCaO_2_ NPs), demethoxylated lignin (DL), and polyacrylamide (PAM) within a glycerol/water binary system. By combining self-oxygenating, anti-freezing, antioxidant, and UV-resistant functionalities, the hydrogel was specifically designed to enhance wound healing under high-altitude conditions (Fig. 1a). In such environments, physiological repair processes are severely compromised by low temperatures and reduced oxygen availability, which collectively impair cellular activity and delay tissue regeneration [108]. Due to the presence of phenolic groups in lignin, the hydrogel scavenged reactive oxygen species and mitigated inflammation, while the controlled decomposition of CaO_2_ enabled continuous oxygen supply under hypoxic conditions. In vivo studies in diabetic and high-altitude wound models demonstrated accelerated wound closure, enhanced angiogenesis, and improved collagen deposition.

The oxygen storage/release approach relies on functionalizing the hydrogels with oxygen carriers such as hemoglobin and perfluorocarbons (PFCs), which can efficiently transport and release oxygen to the targeted tissues [97,106]. Due to the presence of haem groups, organic molecules containing an iron atom bound within a porphyrin ring, various forms of hemoglobin, including natural hemoglobin and myoglobin, are incorporated within the hydrogels to enhance stability and oxygen retention, creating self-oxygenating platforms [111]. In this context, oxygen-carrying hydrogels based on stabilized oxygen nanobubbles have also been proposed as an effective strategy to locally enhance oxygen availability in skin tissue regeneration. For instance, Han et al. [112] have developed an injectable hydrogel based on dopamine-grafted hyaluronic acid (HA-DA) and polydopamine (PDA)-coated Ti_3_C_2_MXene nanosheets through the oxidative coupling of catechol groups catalyzed by H_2_O_2_/HbO_2_. This system enables sustained oxygen levels at the wound site, accelerating wound closure, reducing inflammation, and promoting collagen alignment and epidermal regeneration in full-thickness skin wound models [112].

The PFCs are another class of oxygen carriers that have recently attracted much attention due to their high chemical stability, biological inertness, and exceptional oxygen solubility [113,114]. Their unique molecular structure allows them to absorb oxygen from the environment and gradually release it to the surrounding tissues. PFCs are often encapsulated in microspheres or emulsified into a hydrogel matrix, which serves as a scaffold that holds the PFCs in place, allowing controlled release of oxygen. In aqueous microenvironments, these materials can react with water to intrinsically produce oxygen, which then diffuses through the hydrogel substrate to the surrounding tissues.

Beyond these two approaches, a few hydrogels, such as gelatin methacryloyl (GelMA), can produce oxygen in situ without incorporating external oxygen-generating agents [115]. The structural resemblance of these hydrogels to the ECM, combined with their 3D interconnected network structure, enables high water absorption, thereby enhancing oxygen generation [116]. A relevant example of oxygen-releasing hydrogels specifically designed for skin regeneration was reported by Bai et al. [117]. They developed a self-healing hydrogel incorporating oxygen-releasing microspheres for the treatment of hypoxic chronic wounds. The hydrogel was prepared by dynamic covalent crosslinking between gallic acid-grafted quaternized chitosan (QCS-GA) and oxidized hyaluronic acid (OHA) to enable sustained oxygen release. Under hypoxic conditions, the hydrogel enhanced endothelial cell survival, migration, and tube formation *in vitro*. In vivo, using a hypoxic mouse burn model, the system promoted accelerated wound healing by reducing inflammation, enhancing angiogenesis, and increasing collagen deposition. The hydrogel induced macrophage polarization from a pro-inflammatory M1 phenotype toward a regenerative M2 phenotype, demonstrating the role of oxygen-releasing hydrogels in regulating the wound microenvironment and promoting skin regeneration.

While self-oxygenating hydrogels have been developed by various fabrication techniques, such as solvent casting [118], freeze-drying [119], and electrospinning [120], these strategies face limitations in creating intricate patterns, personalized hydrogels, and scaling up production [121,122]. The solvent casting method lacks precision, leads to non-uniform distribution of oxygen-generating materials, and has a slow solvent evaporation rate, which hinders its scalability [118]. Hydrogels prepared via freeze-drying are fragile and lack durability, limiting their suitability for mechanically robust designs [119]. Since the process depends on freezing conditions, achieving precise and reproducible patterns is challenging.

In contrast, 3D printing has emerged as a promising alternative, enabling the fabrication of oxygenating hydrogels with controlled architecture and porosity [[123], [124], [125]], promoting optimized oxygen diffusion and cellular functionality. This approach supports the development of customized scaffolds with spatially controlled oxygen release profiles tailored to meet the specific requirements and needs of different tissues. To date, different self-oxygenating 3D-printed hydrogels capable of storing and releasing oxygen in a controllable manner have been explored for tissue regeneration [123,126]. Organ-scale engineering and regenerative medicine are often limited by the condition of hypoxia generated within a thick bioengineered tissues, which critically affect cell viability. Krishnadoss et al. [109] introduced a smart self-oxygenating tissue (SSOT) that exploits a bio-ionic liquid (BIL) functionalized hydrogel electrolyte for localized and controlled release of oxygen via electrolysis (Fig. 1b and c). The hydrogel electrolyte was produced by incorporating BIL into gelatin methacrylate (GelMa), then combined with cobalt phosphate (CoP) or platinum (Pt) electrodes for on-demand, localized oxygen release. Oxygen was generated within the electrolyte in a time-dependent manner as the applied voltage was alternately switched on and off. In vitro studies demonstrated that the oxygen-generating capability of the scaffold significantly enhanced cell viability and facilitated rapid vascularization under hypoxic conditions. In a diabetic wound healing model, this platform accelerated wound closure, increased collagen deposition, and promoted angiogenesis [109].

Using a distinct oxygen-generation strategy, Zhu et al. [110] developed a double-layered hydrogel that continuously produces oxygen via photosynthetic cyanobacteria, enabling simultaneous visualization of bacterial infection and oxygen supply to enhance antimicrobial photodynamic therapy (PDT) and alleviate inflammation in diabetic wounds. The inner hydrogel layer, composed of oxidized sodium alginate and carboxymethyl chitosan (CMCS) crosslinked via Schiff-base chemistry, incorporates a photodynamic metal–organic framework (PCN-224) along with the pH indicator bromothymol blue for infection monitoring. The outer hydrogel layer, formed from agarose and CMCS, encapsulated cyanobacteria that continuously generated oxygen, thereby alleviating tissue hypoxia and significantly enhancing PDT efficacy. The hydrogel offers tremendous benefits in the synergistic treatment of refractory anaerobe wounds from timely infection monitoring to tissue repair (Fig. 1d and e).

Some other examples of injectable oxygen-carrying nanocomposite hydrogels are based on mesoporous organosilica, fluorine polymer (PMOF), and alginate (Alg) [123]. These gels were 3D-printed into different patterns to improve the cell viability and differentiation. Analysis of the oxygen generation capacity revealed that the oxygen level in the PMOF and Alg-PMOF-containing scaffolds was around 8%, comparable to the physiological oxygen levels in normal tissues under hypoxic conditions. Interestingly, no difference was observed in results between the hypoxic and normoxic groups owing to the slow diffusion of oxygen from the hydrogels. In vitro studies revealed that the incorporation of oxygen agents significantly increased the viability of fibroblast cells in normal as well as hypoxic conditions over a period of 14 days. However, the survival of malignant Colo-818 cells was reduced due to the enhanced metastatic potential of tumor cells caused by destabilization of the hypoxia-inducible factors. In another study, manganese dioxide (MnO_2_) nanosheets were encapsulated within the natural hydrogel matrix of silk fibroin (SF) and carboxymethyl cellulose (CMC) to convert the excessive reactive oxygen species (ROS) present in the diabetic wounds into oxygen, thereby promoting tissue regeneration and facilitating the ECM remodeling [127]. The released oxygen was found to increase oxygen levels by 17 times and reduce ROS by threefold, alleviating oxidative stress in diabetic microenvironments. This, in turn, decreased inflammation and stimulated angiogenesis *in vivo*.

Not only chemical oxygen-generating groups but also biological agents have been utilized to develop living oxygenating hydrogel scaffolds for prolonged oxygen generation over the entire healing period. Wang et al. [128] fabricated a *Chlorella pyrenoidosa* microalga incorporated fibrous hydrogel scaffold based on alginate and GelMA using 3D bioprinting. Inspired by the mechanism of photosynthesis, the scaffold generated oxygen in situ by converting carbon dioxide and water under light stimulation. This process promoted cell activities, such as proliferation, migration, and differentiation, in hypoxic conditions *in vitro* (as demonstrated by scratch tests and tube formation experiments), while alleviating hypoxia *in vivo*. In a diabetic mouse wound model, the scaffolds reduced hypoxia, accelerated wound closure, enhanced angiogenesis, and supported ECM synthesis. Increased vascular density and collagen deposition were observed, demonstrating their potential to address chronic wound healing challenges by promoting the process of angiogenesis and ECM formation, ultimately contributing to the healing of chronic wounds. Moreover, Chen et al. [129] developed a wound dressing patch incorporating hydrogel microbeads loaded with *Synechoccus elongatus* PCC7942, a photosynthetic cyanobacterium capable of assimilating carbon sources (CO_2_, CO_3_^2−^, and HCO_3_^−^) and producing molecular oxygen upon light exposure. When incubating in a Na_2_CO_3_ solution, the hydrogel produced dissolved hydrogel concentrations up to 1400 μM under near-infrared (NIR) irradiation. Notably, the patch enabled effective oxygen generation and transdermal delivery through murine skin layers measuring 300–400 μm in thickness, achieving local oxygen levels of approximately 240 μM-more than two orders of magnitude higher than those attained with conventional topical oxygen gas therapy. In a diabetic mouse wound model, application of the patch markedly enhanced angiogenesis, re-epithelialization, and tissue formation, thereby accelerating wound closure by converting chronic wounds into an acute healing state.

#### 2.2.2 Nanomaterial-free bioinks

***Protein-based hydrogel inks:*** GelMA is one of the most widely used protein-based biomaterials for biofabrication due to its biocompatibility and tuneable mechanical properties [130]. However, its low viscosity and thermo-sensitivity can pose challenges in producing scaffolds with high fidelity and long-term stability. These limitations can be addressed by developing GelMA-based interpenetrating networks (IPNs). Anand et al. [131] prepared a tuneable IPN hydrogel combining gelatin-hyaluronan dialdehyde (Gel-HDA) with a 100% methacrylamide-substituted GelMA. Various ratios of the two polymeric components were mixed to generate different IPNs (IPN1, Gel-HDA 1% (w/v) + GelMA 9% (w/v); IPN2, GelHDA 3% (w/v) + GelMA 7% (w/v); IPN3, Gel-HDA 5% (w/v) + GelMA 5% (w/v); IPN4, Gel-HDA 7% (w/v) + GelMA 3% (w/v) and IPN5, Gel-HDA 9% (w/v) + GelMA 1% (w/v), by ultraviolet (UV) light exposure after bioprinting. The formulations were analyzed by rheometry using amplitude and frequency sweep tests, resulting in a predominance of Gʹ over Gʺ, indicating the presence of a sample-spanning network structure. A time sweep test allowed to assess a shear equilibrium modulus of the hydrogels and HDA/GelMA IPN resulted in a higher value (1000 Pa) than plain GelMA (600 Pa). A temperature sweep analysis was performed to determine the sol-gel transition temperature (30 °C for HDA/GelMA IPN and 27 °C for plain GelMA) and to establish the working temperature range (10 – 20 °C). Finally, square-shaped constructs (1 cm × 1 cm) were printed in triplicate to evaluate printing fidelity and to identify the optimal printing parameters for the formulations based on the circularity (*C*) of the square-shaped holes (Equation (1)) and the printability parameter (*Pr*) value (Equation (2)):

(1) $$C=4\pi {\text{AL}}^{-2}$$

(2) $$\mathrm{Pr}=\frac{\pi }{4}\frac{1}{C}=\frac{L^{2}}{16A}$$

where *L* and *A* are the perimeter and the area of pores, respectively. According to literature [132], a standard *Pr* value is in the range of 0.9 – 1.1. Depending on the different applied feed rates (5, 10 and 15 mm/s) and plunger velocities (0.05, 0.1, 0.2 mm/s), different *Pr* values in the range of 1.07 to 1.14 were obtained. GelMA filaments broke at 15 mm/s velocity, while IPN ones maintained their structures for all the applied velocities, confirming the increased strength of the IPN formulation resulting from the interlocked microstructure.

In another study, GelMA was exploited to produce semi-IPNs and IPNs in combination with elastin or collagen to obtain a fully protein-based scaffold [133]. Components were photo-crosslinked using genipin or irgacure as photoinitiators. The resulting hydrogels were successfully printed into grid structures. Viscoelasticity of GelMA, semi-IPN, and IPN was monitored by time-resolved rheological analyses. The semi IPN resulted in the highest shear storage modulus G′ (9195.50 Pa), followed by the GelMA polymer (7797.60 Pa) and by the full IPN (6317.60 Pa). Such behavior can be explained by the higher crosslinking degree within the IPN, a factor that compromises elasticity while increasing brittleness. Nevertheless, both scaffolds promoted PC12 cell survival and cellular migration. Analyses of nuclei eccentricity and area studies of the PC12 cells also indicated that the cells were in a proliferative state within the semi-IPN and IPN bioprinted structures.

**Fig. 2:**
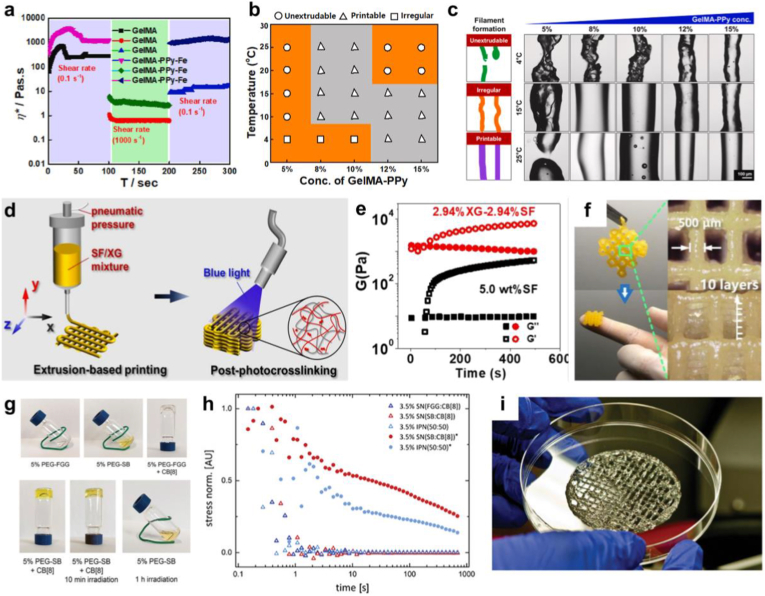
**Preparation and characterization of hydrogel inks for 3D printing**: a) Simulation of the 3D bioprinting process through 3ITT analysis and evaluation of the viscosity recovery; b) and c) Printability diagram of GelMA-PPy ink based on its concentration and temperature profile and qualitative assessment of filament formation (scale bar: 100 μm). Reprinted with permission of [136] Copyright 2024, Elsevier. d) Schematic of 3D extrusion-based printing for developing SF/XG composite gel. e) Time sweep tests of 5 wt% SF and 2.94% XG-2.94% SF hydrogel subjected to in situ UV light irradiation; f) 3D-printed cubic structure of 2.94% XG-2.94% SF hydrogel with a filament thickness of 500 μm and 10 layers. Reprinted with permission of [137] Copyright 2022, RSC. g) Images of solutions and hydrogels, displayed from left to right: 5 wt% PEG-FGG solution, 5 wt% PEG-SB solution, hydrogel formed by 5 wt% PEG-FGG crosslinked with CB [8], hydrogel formed by 5 wt% PEG-SB crosslinked with CB [8], hydrogel of 5 wt% PEG-SB crosslinked with CB [8] after 10 min of irradiation, and hydrogel of 5 wt% PEG-SB without CB [8] after 1 h of irradiation with a 370 nm LED, h) stress relaxation of 3.5 % hydrogel under constant strain of 10%, before and after irradiation with external light source at 370 nm and i) 3D printed cylindrical hydrogel with cell-laden having 50 mm diameter and 320 μm thickness. Reprinted with permission of [138] Copyright 2024, Wiley.

GelMA has also been exploited to form semi-IPN/IPN structures with conductive polymers, which are particularly useful in neuronal or cardiac tissue engineering [134,135]. However, intrinsically conductive polymers often lack optimal shear-thinning properties, i.e., viscosity decreases with increased shear rate and print fidelity, which are fundamental for using a material in 3D bioprinting applications. To address these limitations, forming a semi-IPN with another printable hydrogel may offer a valuable solution. Dutta et al. (2023) developed a semi-IPN of GelMA and polypyrrole (PPy) for direct ink writing (DIW) 3D printing [136]. The resulting construct underwent physical, photo, and ionic crosslinking. The rheological analysis indicated the ink's temperature-dependent behavior, with a solid-like structure (Gʹ of 414 × 103 Pa and Gʺ of 53.02 × 103 Pa) at temperatures below room temperature (RT). Viscosity tests demonstrated the shear-thinning behavior of the resulting ink, confirming its suitability for 3D bioprinting. The amplitude sweep test demonstrated the high stability of the crosslinked hydrogel with a yield point of 100 % shear strain. Additionally, a three-interval thixotropic test (3ITT) underscored the ink's high recovery after being subjected to 1000 s^−1^ shear rate, a common value encountered during extrusion in 3D bioprinting (Fig. 2a). Printability parameters were evaluated by printing a single-layer 20 x 20 × 1 mm construct for each parameter set and calculating strand uniformity (*U*) and *Pr* from microscope images. Screening these parameters based on extrusion temperature and concentration of GelMA-PPy hydrogels allowed classifying the filament as unextrudable, printable, or irregular according to the obtained smoothness (Fig. 2b and c).

Enzymatic crosslinking is an emerging strategy to achieve chemical bond formation between macromolecular chains in a cell-friendly manner. Unlike numerous chemical crosslinking methods that rely on harsh organic solvents and radical species, enzymatic crosslinking offers a gentler approach, promoting biocompatibility while generating a biomimetic microenvironment for native cells, proteins, and tissues [139]. This process is accomplished in the presence of a specific substrate and a reactive group recognized by a particular enzyme. For instance, horseradish peroxidase (HRP) is a highly effective enzyme for crosslinking phenolated conjugates found in natural and synthetic polymers, operating with a low hydrogen peroxide (H_2_O_2_) concentration (10 mM – 30 mM). Liang et al. [139] combined this strategy with enzymatic crosslinking methods to create an IPN hydrogel composed of tyramine-conjugated 8-arm poly (ethylene glycol) (8PEGTA) and GelMA. 8PEGTA was synthesized by activating the hydroxyl end groups of PEG with pyridine, followed by a reaction with tyramine. The two polymeric components were mixed with lithium phenyl-2,4,6-trimethylbenzoylphosphinate (LAP) and HRP, and the IPN was formed through subsequent crosslinking of GelMA via UV light irradiation (365 nm, 1 min, 10 mW/cm^2^) and immersion of the 3D bioprinted construct in a 0.03 wt% H_2_O_2_ solution. Rheological analysis was conducted to study the UV gelation kinetics through in situ photopolymerization, allowing the determination of the optimal UV light exposure time. The Gʹ and Gʺ moduli were monitored over a temperature range from 10 °C to 40 °C to identify the appropriate printing temperature, which was found to be in the range of 22.5 to 24°. The material's stability under incubating conditions (37 °C) was also affirmed by determining constant Gʹ and Gʺ moduli (around 10^4^ Pa and 100 Pa, respectively) over a time interval of 300 s. The 3D printing of cuboidal structures of GelMA and GelMA-8PEGTA IPN revealed that GelMA scaffolds collapsed after one night at 37 °C, while the IPN scaffolds perfectly maintained their shape integrity. Printing of the IPN hydrogel in an inclined tubular structure also demonstrated shape fidelity under deformation.

Another protein-based material that is often exploited in 3D bioprinting is silk fibroin (SF). Huang et al. [137] developed a photo-crosslinkable bioink based on SF with tyramine-modified sodium CMC (CMC-TA), incorporating Ru (bpy)_3_Cl_2_ (Ru(II)) and potassium persulfate (KPS) as photo-initiators, with xanthan gum (XG) used as a thickening agent. The processing involved mixing the precursor, printing the ink, and exposing the scaffold to blue light for 30 s (Fig. 2d). The resulting hydrogel formed a semi-IPN where XG was entangled in the crosslinked SF network and formed covalent bonds with tyrosine residues. The hydrogel's characteristics were studied with a focus on the bioprinting process through rheological analysis, which demonstrated shear-thinning behavior in hydrogels formulated at different ratios: 2.94 % XG-4.85 % SF, 4.76 % XG-4.76 % SF and 7.41 % XG-4.65 % SF. Three interval thixotropy tests (3ITT) under high shear rates (100 rad/s) indicated recovery properties of the bioinks post-extrusion, while photo-crosslinking was tracked via photo rheology. Fig. 2e shows that the presence of XG influenced the kinetics of photocrosslinking, and the addition of XG significantly improved the viscoelasticity of the composite, with Gʹ increasing by > 13 times compared to that of 5.0 wt % SF. The Gʹ and Gʺ moduli of the photocured constructs were also assessed using amplitude sweep rheological measurements, resulting in 10^4^ and 10^3^ Pa, respectively. In addition, the observed increased viscosity at higher strains (>10%), evidenced by a weak strain overshoot, indicated the presence of an interconnected network. Finally, printing tests of a grid pattern showed excellent alignment with the CAD model and high durability and manageability of the printed constructs (Fig. 2f). The authors optimized pneumatic pressure, movement speed (1.5 mm/s), and needle size during these tests to achieve fine filaments. For needle size of 0.5 mm, the optimal pressure was 3.3 psi; for needle size of 0.35 mm, 4.7 psi; and for needle size of 0.3 mm, 10.1 psi. In another study, Dixit et al. [140] used SF to develop a bioink for cartilage tissue engineering. The gradual stiffening through controlled gelation and desirable ionic interactions with transforming growth factor-β (TGF-β), as a promoter of chondrogenic differentiation of MSCs, was studied. Enzymatic crosslinking was performed by sulphated CMC (s-CMC) and tyraminated CMC (t-CMC). The stress–strain curves recorded on day 1, day 14, and day 28 showed no significant change in the slope for both CMC/t-CMC/silk and s-CMC/t-CMC/silk hydrogels. However, a notable increase in peak stress was observed in both hydrogels, rising from approximately 6 kPa to around 60 kPa over a 28-day period. Moreover, frequency sweep analyses, conducted at day 1 and at day 28, confirmed the increase of Gʹ moduli from around 600 Pa to 9000 Pa for s-CMC/t-CMC/silk and from 200 Pa to 3000 Pa for CMC/t-CMC/silk, mimicking the natural process of matrix stiffening during cartilage development.

***Polysaccharide-based inks:*** In addition to protein-based materials, polysaccharides are frequently used in the preparation of hydrogel inks due to their abundance in the decellularized extracellular matrix (dECM). Among them, alginate and hyaluronic acid (HA) are commonly utilized. Alginate is particularly advantageous due to its ready availability, cost-effectiveness, and the presence of lateral carboxylic groups that can be functionalized to improve the material's bioactivity and mechanical stability [141]. Thanh et al. [142] synthesized amine-hyaluronic acid (HA-NH_2_) and aldehyde-alginate (Alg-CHO) and mixed them to promote gelation via a Schiff base reaction. They also developed a copolymer of HA-NH_2_/Alg-CHO with silk fibroin (SF). Rheological studies determined the linear viscoelasticity region (LVER) of hydrogels (10% shear strain), highlighted the dependency of the network structure by the frequency with crossover points occurring between G′ and G″ for HA-Alg (5:5) and HA-Alg-SF at 78 Hz and 61 Hz, respectively. The HA-Alg (5:5) hydrogel demonstrated a higher viscosity (30.4 ± 0.83 Pa s) than that of the HA-Alg-SF hydrogel (12.6 ± 1.27 Pa s). A reduction in viscosity was observed at shear rates exceeding 9.1 s^−1^ for HA-Alg (5:5) and 6.6 s^−1^ for HA-Alg-SF, highlighting their pronounced shear-thinning behavior. Printing tests identified a 5:5 vol ratio of HA-NH_2_ to Alg-CHO as optimal for achieving the best injectability. Liu et al. [143] synthesized a HA/Alg-RGD IPN hydrogel by combining ionic-crosslinking and photo-crosslinking methods. Alginate was functionalized with arginine-glycine-aspartic acid (RGD) to enhance cell adhesion and improve cell-matrix interactions. Rheological analyses revealed the shear-thinning behavior of the HA/Alg-RGD hydrogel and its reversibility before UV irradiation, underscoring the potential of this semi-IPN formulation for 3D bioprinting applications. Strain sweep tests confirmed the suitable recovery properties of the IPN ink for Freeform Reversible Embedding of Suspended Hydrogels (FRESH) 3D printing, where it serves as a supporting bath to print gelatin containing endothelial cells (HUVECs). Gelatin acted as a sacrificial agent, creating microchannels that facilitated the spreading of HUVECs, thereby promoting vascularization within the models. Janarthanan et al. [144] functionalized HA with aldehyde (CHO) groups and CMC/carbohydrazide (CHZ) to obtain a self-crosslinked hydrogel through N-acyl-hydrazone bonding between the two components. Various 3D structures, including lattices, cubes, and tubes were printed with high precision, achieving up to 50 layers, demonstrating the high stability and recovery of the HA enriched with CMC. *In vitro* cytotoxicity studies revealed the high cytocompatibility of the formulated hydrogels, while *in vivo* studies in mice demonstrated their capacity to enhance angiogenesis.

Recent studies have focused on supramolecular hydrogels as promising candidates to mimic the dynamic microenvironment of natural dECM, in contrast to natural or synthetic single networks hydrogels [145]. Reversible bonds enable the network to adapt during cell growth and maturation. To overcome the low viscosity and the need for post-printing modification of these systems based on supramolecular interactions, IPNs were considered. For instance, Wang et al. [138] employed supramolecular IPN hydrogels with tunable physical and chemical crosslinking for 3D bioprinting. The first network was composed of a star-shaped polyethylene glycol (PEG) functionalized with tripeptide phenylalanine-glycine-glycine (FGG) or stilbazolium iodide (SB). These lateral groups were used for host-guest complexation with cucurbituril (CB [8]), forming a physical network. UV irradiation (370 nm) resulted in the photochemical [2 + 2] cycloaddition of SB units within the pre-organized network, leading to a fast and homogeneous gelation. The copolymers and CB [8] were mixed at a ratio of 2:1 with respect to the polymer end groups of CB [8] to prepare IPN hydrogels (Fig. 2g). A 3.5 wt% PEG-FGG solution with varying fractions of PEG-SB at 0%, 25%, 50%, 75% or 100% was used. Stress relaxations analyses were conducted to investigate the extrudability and self-healing properties of IPN hydrogels. When a constant strain of 10% was applied, distinct stress relaxation patterns were observed depending on the composition and method of hydrogel preparation. Due to the reversible nature of host-guest complex formation, physically cross-linked IPNs, such as 3.5% SN (FGG:CB), SN (SB:CB), and IPN (50:50), exhibited complete relaxation within 1s as a result of network disassembly. In contrast, the chemically cross-linked hydrogel (3.5% SN (SB:CB)) showed slower stress relaxation (t_1_/_2_ = 20 s), attributed to polymer chain rearrangements between cross-linking points (Fig. 2h). For the hybrid hydrogel containing equal parts physical and chemical cross-links (3.5% IPN (50:50)), relaxation occurred through both mechanisms, resulting in a faster response (t_1_/_2_ = 2 s) compared to the fully covalent network, closely mimicking the relaxation behavior of natural biological tissues. It was demonstrated that the addition of CB [8] to 5% polymer solutions resulted in hydrogels exhibiting either viscous flow-such as in 5% SN (FGG:CB)- or solid-like properties, as observed in 5% SN (SB:CB). The hydrogel was preloaded with cell suspensions to develop two-layer porous cylindrical scaffolds of various sizes (Fig. 2i). A cell viability assay on 3D bioprinted constructs using SaOS-2 cell lines revealed cytocompatibility, cell proliferation, and post-extrusion cell growth.

#### 2.2.3 Nanocomposite bioinks

Nanocomposite hydrogels can also be engineered to leverage the advantages of nanomaterials, enhancing their functionality for various biomedical applications. The nanoscaled phase may include ceramic biomaterials, which are commonly used in bone tissue engineering for their mineralization-inducing properties; nanoclays or layered silicates, which improve printability and structural fidelity; carbon nanostructures, which have been shown to support neural stem cell differentiation and cardiac tissue engineering; and polymer nano-reinforcements, e.g., cellulose, collagen nanofibers, or polylactic acid (PLA) NPs [[146], [147], [148]]. Hafezi et al. [149] prepared IPNs comprising GelMA, alginate, and nanoclays. Frequency sweep analysis showed the gel-like nature of the network and revealed the effect of nanoclays on the elastic modulus of nanocomposite hydrogels. The nano-reinforcements increased the moduli due to the entanglements and the interactions with the IPN backbone. However, at concentrations beyond 2%, the reinforcing particles aggregate and obstructed the instauration of these interactions. No effect of nanoclays on L929 fibroblast cells viability was noticed, while the degradation rate decreased. Saleki et al. [150] incorporated a covalent organic framework (COF) into a GelMA-alginate hydrogel composite ink. An increased porosity level and enhanced mechanical properties with no effects on cell viability were demonstrated. The printability and rheological behavior of the developed formulations were thoroughly investigated. Viscosity curves revealed shear-thinning behavior across all tested samples, indicating suitability for extrusion-based bioprinting. Notably, the inclusion of COF led to an increase in viscosity—from 2.098 × 10^2^ mPa s for the plain formulation to 3.839 × 10^2^ mPa s for the COF-incorporated hydrogel. Further analysis through frequency sweep and compression tests identified 1 wt% COF as the optimal loading. At this concentration, mechanical performance was maximized without compromising material integrity. However, increasing the COF content to 3 wt % resulted in a reduction in compressive modulus, likely due to COF agglomeration that adversely affected network uniformity. Ng et al. [151] developed an IPN consisting of gellan gum and collagen, incorporating pregelatinized starch (PGF) as lubricant and glycerol as hydrophilic plasticizer. A Cellink BioX™ bioprinter was used to carry out printability tests, where the printing resolution was evaluated using dimensional analysis assay. The filament widths were measured as a percentage relative to the outer diameter of the 27 G bioprinting nozzle (n = 3). A smaller deviation of the average percentage from 100% indicated a higher printing accuracy and resolution, with an average shape fidelity factor equal to 1.36. Step-strain rheological measurements were also conducted to evaluate the shear recovery of the composite bioink. The structural integrity of the composite glycerol–PGF–gellan gum bioink was further evaluated through thixotropic recovery tests. Following the application of a high shear rate (500 s^−1^), the formulation exhibited rapid viscosity recovery, reaching approximately 550 Pa s within 10 s. This observation indicated a satisfactory level of structural resilience and self-healing capacity, critical for maintaining shape fidelity during and after extrusion. In contrast, the control bioink formulation lacking PGF achieved only 250 Pa s viscosity recovery under identical conditions, highlighting the reinforcing and deformation-resistant role of PGF in enhancing the mechanical stability of the bioink. Hu et al. [152] introduced a 3D printable, highly stretchable ternary organic–inorganic nanocomposite hydrogel composed of the thermoresponsive PMeOx-b-PnPrOzi/poly (N,N-dimethylacrylamide) hydrogel and Laponite® XLG nanoclay, which was photocurable. The addition of nanoclay enhanced both the printability and mechanical properties of the composite hydrogel. Improved elongation at break (500 %), flexibility, and compressibility were also reported.

## 3 3D printing techniques

### 3.1 Acellular patches

For most skin applications, 3D-printed hydrogels and patches typically function as acellular scaffolds, where the interplay between ink material, printing technique, and scaffold design enhances printing versatility and advances biomedical functionality. This section explores innovations in fabrication strategies (Table 1) and processing parameters for the development of advanced skin hydrogels and patches.

**Table 1:** 3D-printing techniques for the development of acellular patches for skin applications.

3D printing technique	Concept	Main outcome (advantages and disadvantages)	References
**Extrusion-based printing**	Extrusion of viscoelastic, extrudable gel inks.	**Pro:** High biocompatibility hydrogels and high bioactivity inks	[[153], [154], [155], [156], [157], [158], [159], [160], [161], [162], [163], [164], [165]]
		**Con:** Low mechanical strength and resolution structures	
**Digital light processing (DLP)**	Light-assisted patterned photopolymerization of precursor resin matrix.	**Pro:** High resolution, complexity and diversity microstructures	[[166], [167], [168], [169], [170]]
		**Con:** Limited to photocurable inks	
**Fused deposition modeling (FDM)**	Deposition of high-temperature molten thermoplastic polymers.	**Pro:** High mechanical strength structures, simplicity, scalability and cost effectiveness.	[[171], [172], [173]]
		**Con:** Limited to thermostable materials, low resolution structures	
**Electrowriting**	Precise fibrous deposition of polymer solution or melt under an applied voltage.	**Pro:** High resolution and precision submicron structures	[174,175]
		**Con:** Limited to simple structures	

#### 3.1.1 Extrusion-based 3D printing

For effective extrusion-based printing, the employed inks must possess optimal viscosity and consistency to ensure both architectural precision and mechanical integrity of the printed patches. With the growing demand in 3D printed patches, there is ongoing research focused on innovative, readily extrudable ink compositions for skin application. Naik et al. [153] developed an *Aloe vera* gel-integrated amyloid fibril hydrogel based on bovine serum albumin (BSA) for chronic wound healing. Extrudable hydrogel inks were prepared via heat-induced self-assembly of BSA into mechanically stiff amyloid fibrils, where the integration of *Aloe vera* gel resulted in tunable ink viscoelasticity. The printed square-shaped lattice structure (3 × 3 × 0.5 cm) exhibited high shape fidelity with structural microporosity (pore area around 25 μm^2^). For wound healing, readily extrudable inks were prepared by combining gellan gum with starch extracted from the arrowroot plant [154]. Prior hydrothermal treatment of starch facilitated the formation of coiled-amylose structures and promoted matrix gelation. In addition, increasing starch concentration improved ink architectural precision. The printed square-shaped lattice scaffold (20 × 20 × 1 mm) demonstrated a microporous structure (40 -130 μm). In another study [155], a hyaluronic acid (HA)-based hydrogel chemically conjugated with tyramine and yeast-derived ACE-inhibitory peptide was developed for wound healing. Compared to plain HA, peptide- and tyramine-functionalized HA exhibited enhanced printability and structural stability of the printlet **(**printed construct), which was facilitated by photo-crosslinking of the conjugated tyramine. In contrast to the poor structural stability observed with plain HA after printing, the peptide and tyramine-functionalized HA demonstrated high shape fidelity (79.98 ± 3.08% pore squareness) through the photo-crosslinking process.

Another attractive class of materials for extrusion-based printed patches are tissue/biologically derived inks. With multiple reactive motifs, extracellular matrix (ECM)-based inks enrich printed hydrogels with various cellular cues for more efficient biological functionality. However, ECM materials alone lack the necessary rheological and mechanical properties to ensure optimal printability and structural fidelity after printing. To address this, various approaches have been explored to enhance the overall printing performance of ECM-based inks. For example, Bashiri et al. [156] developed a composite ink comprising placental dECM, sodium alginate and gelatin for deep wound healing. Printed disc-shaped hydrogel scaffolds (7 mm diameter) were crosslinked with calcium chloride and glutaraldehyde. Increasing the ECM concentration in the inks (from 0% to 5%) resulted in scaffolds with improved compressive strength (ranging from 0.67 to 1.24 MPa) and enhanced swelling behavior (116.33 to 136.16% at 0.5 h). Similarly, hydroxyethyl cellulose and laponite were blended with amnion membrane dECM to create rheologically suitable, easily extrudable inks for skin tissue engineering [157]. It was demonstrated that 2% w/v laponite enhanced the shear-thinning behavior of the ink and improved printability, resulting in a spreading ratio of 1.7 for a square-shaped lattice scaffold (3 × 3 cm). In another study [158], a tissue-specific ink based on dECM from porcine skin, gelatin, quaternized chitosan, and poly (ionic liquids) was used for printing square-shaped, lattice scaffolds (10 × 10 × 2 mm) for prospective skin tissue engineering. The developed ink exhibited good printability and shear-thinning properties, while the printed scaffolds displayed high structural integrity and microporosity after printing (90 μm).

**Fig. 3:**
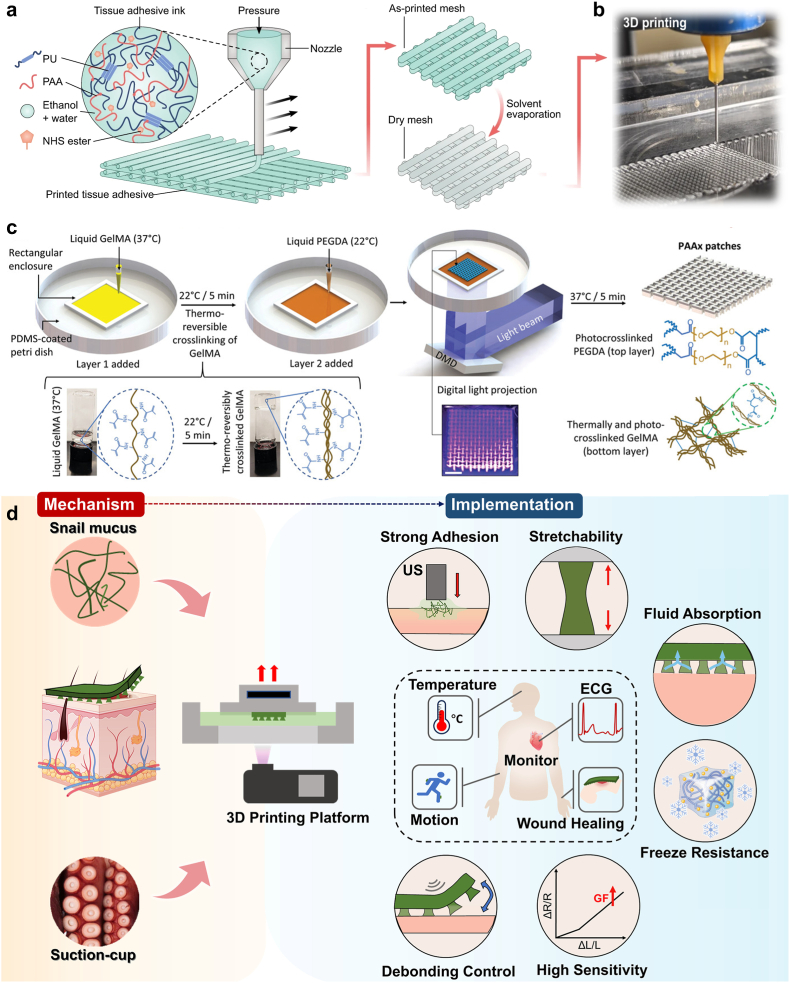
**Extrusion 3D-printing of tissue adhesive patch: a)** Overview of the printing process for the PAA-NHS-PU adhesive ink into a dry mesh that hydrates on contacting the skin, initiating amide linkage-associated adhesion and **b)** 3D printed patch morphology. Reproduced with permission from Ref. [159] Copyright 2024, Nature. c) Fabrication of the bilayered auxetic patch with multi-direction anisotropic stretchability employing 3D printing of GelMA and PEGDA followed by thermal and photo crosslinking. Reproduced with permission from Ref. [163]. Copyright 2022, Wiley. d) UV-digital light processing for the development of 3D printed diagnostic-therapeutic ultrasound-mediated, adhesive skin-electronic interface based on acrylamide hydrogel and inspired by sucker microstructures and snail mucus. It exhibits mechanical and electronic properties, moderate water swelling, and antifreeze capabilities, making it applicable for temperature sensing, motion and ECG monitoring, and frostbite wound healing. Reproduced with permission from Ref. [167] Copyright 2024, Elsevier.

A versatile tissue-adhesive ink was developed for 3D printing of various bio-adhesive patches and devices, comprising poly (acrylic acid)-N*-*Hydroxysuccinimide (NHS) ester grafted to polyurethane (PU) [159]. Upon application of the dry mesh patch to a hydrated tissue surface, adhesion is initiated via covalent amide linkage between tissue amine groups and PAA-NHS ester. The developed patch achieved rapid adhesion with effective sealing potential for over 4 weeks (Fig. 3a and b). In addition to ink materials, patch design can be strategically optimized to print scaffolds with high versatility. For example, Cui et al. [160] developed composite tailorable wound dressings by printing three chitosan/glycerol micropatterns (sheet, strip, and mesh) with different spacing pitches between filaments. The micropatterns were printed on a commercial dressing substrate (with a top perforated polyethylene film). When microscopically examined, the printed structure (square of 3 cm side length) demonstrated different patterns: plain solid surface, parallel bands, and mesh strips for sheet, strip, and mesh micropatterns, respectively, with high geometric precision.

Integrating 3D printing with other fabrication techniques is crucial for creating more diverse scaffold designs capable of replicating skin complexity and enhancing biological functionality. For example, Shahriari-Khalaji et al. [161] integrated extrusion 3D printing and electrospinning to develop a bilayer hydrogel scaffold as a skin substitute for infected burn wounds. Inks comprising carboxymethyl chitosan, oxidized alginate grafted catechol, gallium, and platelet-rich fibrin were 3D printed on a hydrophobic gallium-PCL electrospun layer, simulating a functional dermal layer and a backing epidermal layer, respectively. The printed, disc-shaped scaffold (7 mm in diameter) exhibited a microporous architecture with a pore size of 63 μm, while the backing layer displayed a microfibrous entanglement. The printed, disc-shaped scaffold (7 mm in diameter) featured a microporous architecture with a pore size of 63 μm, while the backing layer exhibited a microfibrous entanglement with a fiber diameter of 2 μm. In a similar approach, Marjan et al. [162] fabricated a three-layered scaffold for skin tissue regeneration by combining 3D printing and electrospinning. The top layer, an electrospun polyurethane (PU) nanofibrous coating, prevents microorganism penetration. The middle layer, 3D printed from Pluronic F127, quaternized chitosan, and silver nitrate NPs, serves as a porous absorbent and antibacterial layer. The bottom layer features a core-shell nanofibrous structure of F127-mupirocin/pectin-keratin for tissue regeneration and antibacterial activity. This trilayer structure afforded high antibacterial activity, cell adhesion, proliferation and angiogenesis (around 0.6 blood vessel density/area). Chansoria et al. [163] developed a bilayered adhesive patch, embracing organ-associated anisotropic and auxetic features (Fig. 3c). Extrusion 3D-printing was employed for patch fabrication, where the bottom layer comprised GelMA and the top layer was made from PEGDA, followed by thermal and photo crosslinking. The auxetic patch could conform to the volumetric dynamics of different organs more efficiently than non-auxetic patches.

Tunable macroporous structure could be developed via computer-aided wet spinning (CAWS), coupling extrusion printing and wet spinning into a polymer non-solvent bath [164]. Carboxymethyl chitosan-based scaffolds (20 mm squares) were deposited as microfibrous structures (<60 μm fiber diameter), crosslinked with Zn^2+^ to impart pH-sensitive water uptake, and functionalized with collagen grafting post-printing for improved breaking strain. The composite structure supported high fibroblast cell proliferation (approximately 200% after 7 days) and migration (around 90% after 24 h). Nanofibrous microspheres were also incorporated into an extrusion 3D printed scaffold for diabetic wound healing [165]. Polylactic acid (PLA) nanofibrous microspheres (100 nm fiber diameter) functionalized with DDAB-modified ZnO were integrated into a chitosan/HA ink to print disc-shaped scaffolds (15 mm diameter) at 70% ink flow. The printed scaffolds exhibited multi-level porosity with microsphere and scaffold pore sizes of 20 μm and 160 μm, respectively.

#### 3.1.2 Digital light processing

Among popular photocuring methods, vat photopolymerization, particularly digital light processing (DLP), has proven to be a highly functional and versatile technique for skin-related applications. The photocurable gel matrix precursor can be tailored with additives to enhance printing resolution [166] and printed patch functionality [167]. DLP has been employed for the development of skin medical devices/skin sensors [167], re-entrant auxetic structures [168], radiation therapy bolus [166] as well as skin regenerative patches [169], among others. DLP enables the fabrication of diverse structural architectures. Ma et al. [167] developed a diagnostic-therapeutic integrated patch serving as an ultrasound-mediated adhesive skin-electronic interface. Bioinspired by adhesion of octopus-sucker (suction cup microarchitecture) as well as snail mucus (high viscosity), patches were developed via UV-DLP curing of acrylamide, polyethylene glycol diacrylate (PEGDA; crosslinker), MXene (metal carbide/carbon nitrides) nanosheets (conductivity and cohesion enhancer), and laponite precursor gel (Fig. 3d). When applied to porcine skin pretreated with a chitosan solution and exposed to ultrasound, the developed patches demonstrated strong ultrasound-mediated adhesion to the skin. The bioinspired patch demonstrated moderate swelling properties, high deformability (460%), and tough adhesion (shear increased by 109%). In another study, Tsegay et al. [168] employed DLP to print a pH-indicating wound dressing with a re-entrant auxetic structure, enabling effective adhesion to complex body contours and joint areas without wrinkling or surface instability. A UV-photocurable hydrogel resin was used, comprising phenol red-integrated hydroxyethyl methacrylate, PEGDA, (trimethyl benzoyl) phosphine oxide (TPO; photoinitiator), and acrylamide. The printed patch showed high tensile strength (140 kPa), 14% swelling capacity and 1.2% porosity. To increase the mechanical strength of photo-curing hydrogels, He et al. [170] introduced multiple strengthening mechanisms for fabricating a blue-light (450 nm) DLP-printed hydrogel for wound healing. The precursor gel was based on acrylamide-2-methyl-propanesulfonic acid reinforced with carboxymethyl cellulose nanofibers (NFs). Additionally, double photoinitiators (TPO and laponite) and dual crosslinking (PEGDA and CaCl_2_) were used. The developed structure demonstrated good mechanical properties (0.15 MPa ultimate stress) and high-water absorption and retention capacities.

#### 3.1.3 Fused deposition modeling

Fused deposition modeling (FDM) is widely regarded as a simple and cost-effective additive manufacturing tool for fabricating structurally versatile and mechanically robust skin patches. When used to develop advanced skin patches, research often combines FDM with other fabrication techniques to broaden the scope and enhance the material properties and biomedical applications. For example, Park et al. [171] combined electrospinning and FDM for the fabrication of a scaffold with a fine pattern for developing an auxetic structure for burn wound contractures at joints. The bilayer scaffold comprised an electrospun PCL layer (mimicking the epidermis) and a 3D printed PCL auxetic layer (mimicking the dermis), patterned as a cut-missing rib. The printed structure exhibited a negative Poisson's ratio-a negative ratio of transverse strain to lateral or axial strain-ranging from −0.5 to −0.1. The structure expanded in the direction perpendicular to stretching with reduced deformation (<60%). Fibroblasts incubated with the printed structure exhibited significant cell attachment and proliferation over 7 days. Along the same line, a tunable porosity scaffold was developed via FDM and electrospinning for wound healing [172]. The bimodal patch consisted of a printed PCL micro-mesh with a grid geometry and interconnected micropores, along with PCL/gelatin/polylysine fibrous layers. The composite patch exhibited micro-scale (300 μm) to nano-size (400 nm) pores, and optimum mechanical features (4 MPa tensile strength). In a different context, FDM printing was combined with an in-house molding process to fabricate a shell-core structured skin collimation patch designed to protect healthy tissue during skin cancer radiation therapy [173]. Hollow PLA molds, serving as the shell material, were printed using FDM according to a 3D-scanned human face. Molten Cerrobend, a lead-containing alloy used as the core material, was subsequently cast into the molds to create the collimation patches. The 3D-printed molds demonstrated micron-level conformity to the scanned facial structure, ensuring precise fit and functionality. Hollow PLA molds (shell material) were FDM-printed, exhibiting the shape of a 3D scanned human face structure. Afterward, molten Cerrobend (lead-containing alloy; core material) was poured for the development of the collimation patches. The 3D printed molds efficiently presented micron-level conformity with the scanned structure.

#### 3.1.4 Electrowriting cellular patches

Recently, electrowriting has emerged as a high-precision, high-resolution additive manufacturing technique, in which a polymer, either in solution [174] or molten form [175], is precisely deposited under an applied voltage to form fibrous-patterned skin patches. To investigate the influence of electric fields and substrate topography on directional cell migration during wound healing, solution electrowriting was used to fabricate guided straight-line patterns composed of 0.2% multiwalled carbon nanotubes (MWCNs) embedded in a PCL conductive matrix [174]. As the concentration of MWCNTs increased from 0.1% to 0.4%, both ink conductivity and viscosity increased accordingly. The printed lines provided topographic cues with multilevel line spacing, narrow (50 μm) and wide (400 μm). Molten PCL was also utilized for melt electrowriting of scaffolds modified with yeast-derived peptides for wound healing [175]. Plasma treatment was further performed to endow surface hydrophilicity to the written PCL scaffold and to facilitate peptide functionalization.

### 3.2 Cell-laden 3D printed hydrogels and patches

Bioprinting has rapidly gained attention for enabling the effective fabrication of 3D cell-laden hydrogel structures with high spatial precision, an achievement previously limited by various fabrication challenges. Here, the hydrogel matrix favorably provides the essential nurturing microenvironment, while the hosting scaffold microarchitecture securely harbors the seeded cells [176]. These cell-scaffold structures guide cell proliferation, differentiation, and multidirectional migration through customizable topographic cues. These tunable cell–hydrogel 3D assemblies have greatly advanced the use of AM in skin tissue regeneration and the development of *in vitro* models for mechanistic investigation of various skin pathologies.

**Fig. 4:**
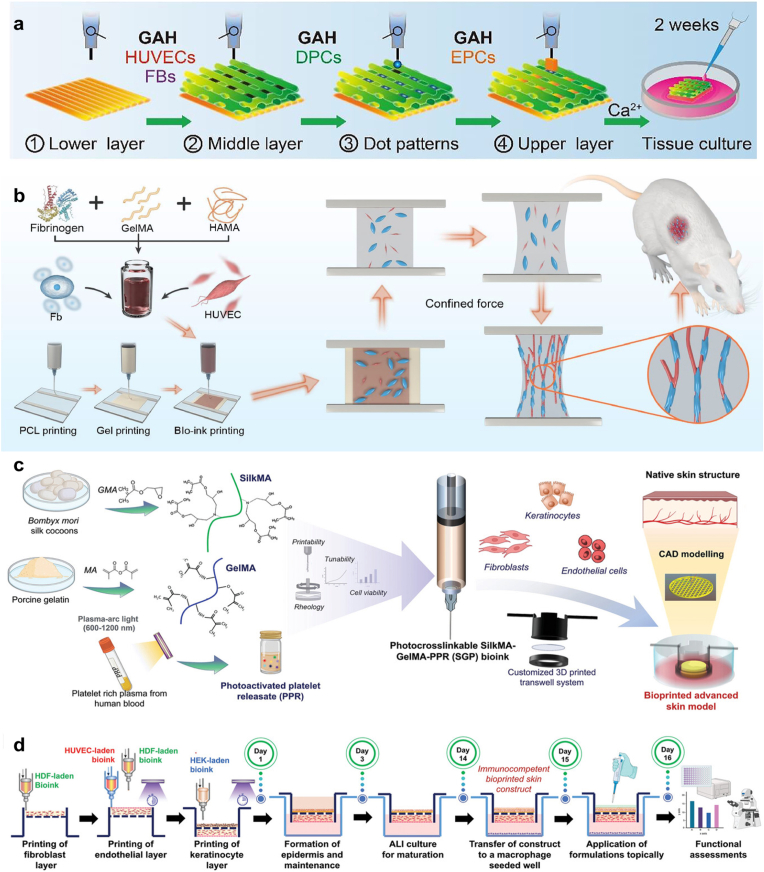
**Different designs of 3D-printed, cell-laden hydrogels.** a) A scheme for the bioprinting of a gelatin/alginate multilayer scaffold for hair follicle regeneration. Reproduced with permission of [179] Copyright 2023, Elsevier. b) A scaffold comprising cell-laden GelMA/HAMA/fibrin bioink printed within a PCL confining framework to promote prevascularization. Reproduced with permission of [180] Copyright 2023, Elsevier. c) Concept and formulation of silk fibroin/GelMA and platelet releasate bioink. d) Stepwise flow of the study. Reproduced with permission of [181] Copyright 2024, Wiley-VCH.

A scaffold is an engineered construct that mimics the cellular environment or ECM, providing a supportive platform for cell seeding. To achieve a defined therapeutic objective, the scaffold design must consider a range of factors, including material properties, architecture, and biological compatibility [177]. Equally critical is the selection of cell types to ensure the development of a functionally effective cell-laden hydrogel scaffold. In this context, particular emphasis is placed on the structural design of scaffolds fabricated through 3D bioprinting [178]. The microarchitecture of such scaffolds can be precisely engineered to enable spatial control over the distribution and behavior of encapsulated cells, especially when incorporating multiple cell populations. For example, a multilayer scaffold was successfully 3D printed for hair follicle regeneration using four different cell types, including fibroblasts, human umbilical vein endothelial cells (HUVECs), dermal papilla cells, and epidermal cells (Fig. 4a) [179]. Three bioinks were prepared from gelatin and alginate, hosting either fibroblasts and HUVEC (bioink 1), dermal papilla cells (bioink 2) or epidermal cells (bioink 3). The multilayer structure enhanced dermal papilla cell proliferation by 1.2-fold and promoted their self-assembly into spheroids with upregulated expression of hair induction–associated genes. Then, 3D printed square-shaped lattice scaffolds (10 × 10 × 2 mm) were deposited into three layers comprising a lower dermis layer (bioink 1), a middle follicle appendant layer (bioink 1 and dot-printed pattern bioink 2), and an upper epidermis layer (bioink 3). In a similar context, a dermal layer was engineered for the development of a prevascularized artificial skin (Fig. 4b) [180]. The printed construct was fabricated by using a bioink composed of gelatin methacrylate (GelMA, 3%) and methacrylated hyaluronic acid (HAMA) at a 15:1 ratio, fibrin (5 mg/mL), and laponite, encapsulating HUVECs and fibroblasts. This bioink was printed within a supportive PCL framework. The contractile activity of the encapsulated cells generated confining forces against the mechanically resistant PCL enclosure, effectively guiding the formation and alignment of vascular networks.

To provide specific cellular cues, scaffold composition must be carefully optimized. For instance, microstructural alignment can be achieved by incorporating shear-responsive filler materials. This was exemplified by a cell-adaptive, 3D-printed hydrogel with a highly oriented, anisotropic microporous structure using a bioink composed of GelMA, sodium alginate, and laponite, integrated with shear-aligned polyethylene oxide filler [122]. The bioink encapsulated NIH/3T3 fibroblasts, and the printed hydrogel grids (15 × 15 × 1 mm^3^) were crosslinked sequentially using CaCl_2_ and UV irradiation. This intricate composition promoted fibroblast-to-myofibroblast transition, establishing an engineered dermal akin layer. A bilayer skin construct was further developed by the coculture of HaCaT keratinocytes atop the printed dermal layer. Along the same line, Fu et al. [182] utilized a pre-gel comprising adipose-tissue derived dECM, GelMA, HAMA and photoinitiator for the bioprinting of human adipose-derived stem cells (hADSC)-laden skin substitute. The adipose tissue-derived dECM provided an inherently supportive microenvironment for the embedded hADSCs, exhibiting thermosensitive gelation at 37 °C. Meanwhile, GelMA and HA enhanced the mechanical properties of the photo-crosslinked, disc-shaped scaffold (8 mm in diameter), resulting in a uniform pore morphology with an average diameter of 73 μm.

The ECM serves as a favorable bioink matrix for fabricating cell-laden scaffolds, derived from both mammalian [183] or marine [184] sources. Notably, fish-derived dECM, compositionally similar to its mammalian counterpart, has been utilized for skin cell bioprinting [184]. Recently, decellularized ECM from Korean amberjack fish skin has been integrated into HAMA and divinyl sulfone to formulate a composite bioink for constructing a bilayer artificial human skin. It has been demonstrated that increasing ECM content enhances ink injectability, printability, and hydrogel swelling (>1500% at 120 min, at 30% w/w ECM). The printed hydrogel provides a supportive environment for the coculture of human dermal fibroblasts (HDF) and keratinocytes (HaCaT), promoting cell proliferation, keratinization, and the formation of distinct epidermal and dermal layers.

Despite significant advancements, preserving cell viability during crosslinking remains a major challenge, as the process often compromises the survival of embedded cells. Zhang et al. [185] addressed this issue by employing dual-sided photo-crosslinking (from both the front and back) to fabricate a 3D skin organoid laden with HaCaT keratinocytes, dermal fibroblasts, and HUVECs for full-thickness skin repair. The GelMA–laponite bioink was printed into disc-shaped constructs (10 mm diameter, 1 mm thickness). This dual-light strategy ensured uniform crosslinking while minimizing localized cell damage. The resulting organoid displayed a skin-like architecture, with surface keratinocytes encapsulating a stromal core, and could be customized to match wound geometry.

Beyond therapeutic applications, 3D printed hydrogels are increasingly leveraged to replicate the physiological complexity of normal skin [186,187] and to model pathological conditions for in-depth mechanistic investigations [188,189]. Physiologically relevant skin models of variable designs have been developed for different testing purposes. For instance, Girard et al. [186] prepared a full-thickness, well-differentiated skin model for dermo-cosmetics and pharmaceutical testing by combining electrospinning and melt electrowriting. A bilayer membrane/scaffold model was developed by first electrospinning a PCL solution to form a nanofibrous epidermal/dermal interface membrane seeded with keratinocytes, followed by electrowriting of a porous PCL microfibrous scaffold as the dermal layer to host fibroblasts. Changing the microfibrous dermal scaffold structure from a straight fiber-design to a wavy, sinusoidal fiber-design resulted in more heterogenous collagen matrix organization in the neosynthesized ECM, creating a tailorable skin model for prospective testing. In another study, Bhar et al. [181] developed a 3D skin model for the assessment of skin sensitization. The bioink matrix was fabricated by extrusion-based printing of methacrylated silk fibroin, GelMA and photoactivated human platelet (Fig. 4c and d). A sandwich type structure was created on both sides of an artificial basement membrane, with a keratinocyte-laden epidermis on one side and a HUVEC/HDF-laden dermis on the other. The developed immunocompetent model could differentiate between irritant and non-irritant substances when transferred to a macrophage-seeded well.

Physiologically relevant skin models of variable designs have been developed for different testing purposes. For example, Choi et al. [187] employed DLP to fabricate a full-thickness skin model as a platform for biomaterial testing and mechanistic studies. A bioink composed of methacrylated silk fibroin and GelMA was 3D printed into three distinct layers: a vascularized dermis (with HUVECs and HDFs), an avascular dermis (with HDFs), and an epidermis (with keratinocytes). Skin wounds were modeled by printing scar shapes into each cell layer. The resulting trilayer construct supported robust cell proliferation and effectively simulated wound healing following epidermal growth factor treatment.

A metastatic melanoma model was created for drug screening using a trilayer scaffold [188]. Human amniotic membrane-derived dECM served as bioink for extrusion-printed basement membranes, while porcine skin dECM was used for inkjet-printed dermis and epidermis. Stromal, metastatic, and nonmetastatic cells were pre-labeled with tracking probes. This model effectively mimicked early cancer cell migration across the basement membrane and demonstrated potential in anticancer drug evaluation. To replicate the skin tumor microenvironment, López de Andrés et al. [189] developed a malignant melanoma model as a multicellular, trilayered 3D-printed construct using an agarose/collagen bioink. The construct consisted of mesenchymal stem cells in the bottom layer, fibroblasts and HUVECs in the middle layer, and cancer stem cells with keratinocytes in the top layer. The scaffold supported robust cell proliferation, vascularization, and metabolic activity, and exhibited a cytotoxic response to vemurafenib.

### 3.3 Microneedle array patches

**Fig. 5:**
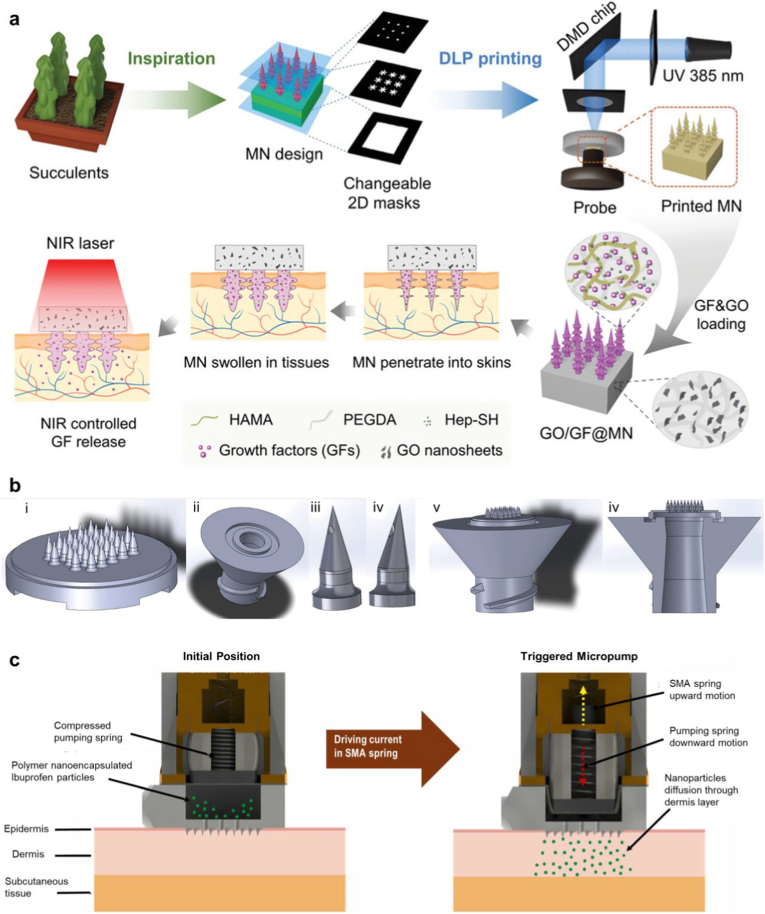
**3D printing of various design-microneedle array patches (MAPs). a)** Concept, design and fabrication of succulent-structured MAPs comprising PEGDA for the hard base and PEGDA/HAMA for the tips. Reproduced with permission of [190] Copyright 2024, Wiley-VCH. **b)** 3D-printed components of the hollow MAPs-Luer lock assembly: (i) High-resolution MAPs array. ii) Luer lock connector. iii) Concentric and iv) Half Eccentric needle tips. v) Assembled device. f) Cross-section view of the assembled device. Reproduced with permission of [192] Copyright 2024, Wiley-VCH. c) The drug release triggering mechanism of the electromagnetically actuated, 3D-printed micropump integrating MAPs. Reproduced with permission of [193] Copyright 2024, Elsevier.

As skin patches, microneedle array patches (MAPs) have significantly advanced through 3D printing, allowing precise customization via simple parameter adjustments. Succulent-structured hydrogel MAPs were recently developed using DLP with variable 2D masks, incorporating PEGDA for a rigid base and PEGDA/HAMA for moisture-sensitive tips [190]. These MAPs exhibited reversible shrink-swell behavior, remaining rigid for skin puncture when dry, and swelling post-insertion to ensure strong tissue adhesion and prolonged drug release (Fig. 5a). In another study, FDM enabled the fabrication of PLA MAPs by horizontally elongating molten films, with tunable printing speed and extrusion length yielding conoid or neiloid shapes featuring smooth surfaces and efficient skin penetration [191].

The integration of MAPs into multifunctional biomedical microdevices has been enabled by combining various 3D printing techniques [194,193]. For example, Islam et al. [194] reported a 3D-printed hollow MAP system with a Luer-lock-integrated reservoir for secure connection and uniform fluid delivery via microchannels. Micro-SLA was used to print the hollow MAPs, while mesoscale SLA printed the Luer-lock reservoir. Two tip geometries—half-eccentric and concentric conical—were evaluated for penetration efficiency (Fig. 5b) [192]. Sedky et al. [193] introduced an electromagnetically actuated micropump integrating MAPs for rapid, painless drug delivery. The PLA casing and polyurethane cover were FDM-printed, while the MAPs were fabricated via stereolithography using photosensitive resin. Upon activation by a 200 mA current, a shape-memory-alloy spring triggered drug release from a chamber loaded with ibuprofen-loaded PLGA NPs, with MAPs enabling efficient transdermal delivery (Fig. 5c).

Recent advancements have explored the use of artificial intelligence (AI), including machine learning (ML) and deep learning (DL), with 3D printing as an emerging strategy to enhance MAPs design and fabrication. ML models can capture the relationships between printing parameters (e.g., layer thickness, flow rate) and the resulting feature (e.g., shape fidelity, mechanical strength), thereby enabling predictive and real-time adjustment of printing conditions [195]. This could minimize material waste and could improve fabrication consistency. ML algorithms could unlock deeper insights into additive manufacturing processes, such as predicting material properties, optimizing designs, and improving the production quality. Further to parameter optimization, DL architectures can forecast microneedle performance from geometric descriptors (e.g., needle diameter, needle height), identifying suboptimal designs before printing [196]. A semi-supervised machine learning approach using a convolutional neural network (CNN) was applied to optimize the DLP printing of dissolvable, ibuprofen-loaded PEGDA MAPs [197]. The CNN model analyzed training data to predict final printing parameters, improving print fidelity and needle geometry. Despite the advantages of 3D printing, printed MAPs often suffer from suboptimal skin penetration. To address this, Razzaghi et al. [198] investigated the impact of printing angle on PEGDA MAPs produced via DLP. Their findings revealed that a 45° tilt angle minimized the insertion force required for effective skin puncture.

### 3.4 Artificial intelligence and 3D printing

Recent advances in machine learning (ML), deep learning (DL), and computer vision (CV)—key components of artificial intelligence (AI)—are increasingly being integrated into 3D printing workflows to address persistent challenges in bioink formulation, process control, and construct reproducibility, thereby expanding the efficiency, robustness, and sustainability of additive manufacturing processes [199,200]. This innovative synergy between AI algorithms and additive manufacturing is transforming the fabrication of printed hydrogels for biomedical applications from a time- and resource-intensive trial-and-error approach into a data-driven and predictive manufacturing paradigm.

During the formulation phase, AI-based strategies employing DL, support vector machines (SVM), Bayesian optimization, and explainable models enable rapid predictive modeling, AI-guided experimental iteration, and accelerated optimization of hydrogel compositions [201]. For instance, Chen et al. (2023) developed decision tree, random forest (RF), and DL models to predict the 3D printability of 210 biomaterial formulations comprising 16 bioactive or smart materials and four solvents. While all models successfully captured formulation–printability relationships, RF achieved the highest overall performance (accuracy: 88.1%, precision: 90.6%, F1 score: 87.0%), whereas DL exhibited the highest recall (87.3%) and generated the most refined printability maps, enabling accurate delineation of printability windows.

Beyond formulation optimization, AI-driven computer vision has enabled real-time process monitoring and defect detection. Sani et al. [202] developed a dual-camera, real-time defect-detection system to identify common extrusion defects during active 3D printing. Using a custom dataset of stringing, spaghetti, under-extrusion, and over-extrusion defects, lightweight YOLOv11n and YOLOv12n models were fine-tuned via transfer learning, achieving high detection accuracy and precise localization while maintaining real-time performance. AI models further enable inverse screening and reverse design by establishing quantitative, data-driven mappings between hydrogel composition and functional performance. Cadamuro et al. [203] reported a user-friendly ML-based framework for designing ECM–mimicking hydrogels with predefined rheological properties for 3D bioprinting; using click-chemistry crosslinking and a limited experimental dataset. The model accurately predicted gelatin-to-hyaluronic acid ratios required to achieve targeted mechanical behaviors.

AI-driven inverse design platforms have enabled the creation of previously unknown, multifunctional biomaterials tailored to complex clinical requirements. In this context, Jiang et al. [204] developed an AI-guided antimicrobial peptide hydrogel design platform that integrates generative modeling with multi-objective optimization, leveraging transformer-based generative models, prompt-tuning, and reinforcement learning. The resulting AI-designed antimicrobial peptide (AI-AMP) was incorporated into hydrogel networks, achieving bactericidal efficiencies exceeding 99.99% against methicillin-resistant *Staphylococcus aureus* (MRSA) and a wound healing rate of 99.5% in a rat model of MRSA-infected full-thickness wounds. Collectively, these studies underscore the transformative potential of AI-enabled reverse design strategies in accelerating the development of multifunctional, application-specific hydrogel systems for advanced biomedical and translational applications.

Although the synergy between AI and 3D bioprinting remains at an early stage, it holds remarkable potential to revolutionize the fabrication of complex biological constructs for tissue engineering and regenerative medicine [205]. In particular, AI-aided monitoring of bioprinting processes enables automated detection, classification, and correction of printing errors during fabrication, thereby enhancing process robustness, construct fidelity, and reproducibility. When integrated with closed-loop control systems, these AI-driven frameworks allow dynamic adjustment of printing parameters in response to real-time feedback, paving the way toward autonomous, standardized, and clinically scalable bioprinting platforms [206].

Recent advancements have also explored the use of AI tools with 3D printing as an emerging strategy to enhance MAPs design and fabrication. ML models can capture the relationships between printing parameters (e.g., layer thickness, flow rate) and the resulting feature (e.g., shape fidelity, mechanical strength), thereby enabling predictive and real-time adjustment of printing conditions [193]. This could minimize material waste and improve fabrication consistency. ML algorithms enable unlocking deeper insights into additive manufacturing processes, such as predicting material properties, optimizing designs, and improving the production quality. Further to parameter optimization, DL architectures can forecast microneedle performance from geometric descriptors (e.g., needle diameter, needle height), identifying suboptimal designs before printing [192]. A semi-supervised machine learning approach using a convolutional neural network (CNN) was applied to optimize the DLP printing of dissolvable, ibuprofen-loaded PEGDA MAPs [195]. The CNN model analyzed training data to predict final printing parameters, improving print fidelity and needle geometry. Despite the advantages of 3D printing, printed MAPs often suffer from suboptimal skin penetration. To address this, Razzaghi et al. [196] investigated the impact of printing angle on PEGDA MAPs produced via DLP. Their findings revealed that a 45° tilt angle minimized the insertion force required for effective skin puncture. Overall, AI is expected to enable fully data-driven 3D printing of MAPs, optimizing geometry, materials, mechanical performance, and drug-loading properties while ensuring real-time monitoring, high precision, and reproducibility. These advances pave the way for scalable production of microneedles tailored for drug delivery [207], vaccines [208], biosensing [209] and other biomedical applications, accelerating both preclinical and clinical translation.

## 4 4D printing

**Fig. 6:**
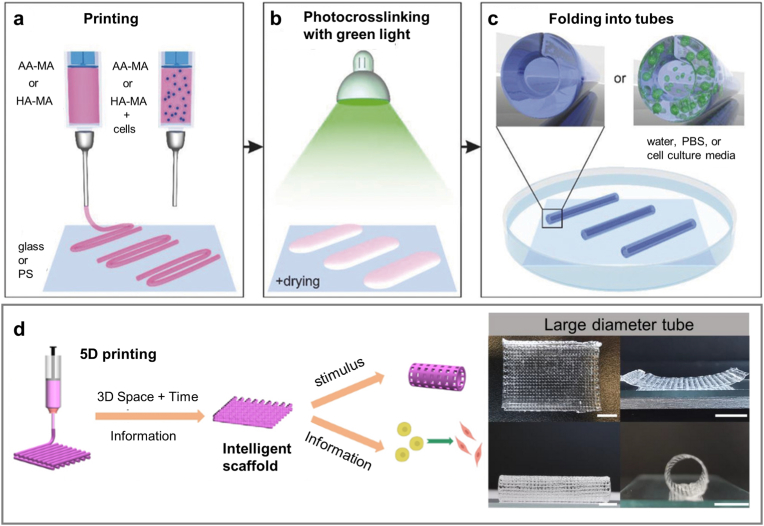
Mechanism of 4D and 5D printing techniques**:** a) printing of AA-MA or HA-MA polymer solutions without/with living cells onto different substrates; b) crosslinking of the printed films with green light and mild drying; c) instant folding into tubes upon immersion of the crosslinked films in water, PBS, or cell culture media. Reprinted with permission of [219] Copyright 2017, Wiley-VCH, d) Schematic illustration of 5D bioprinting of bilayer cell-scaffold constructs with shape-morphing ability (provided by the first layer) and in situ delivery of information (provided by the second layer). Images show the top and side views of large tubular poly (d,l-lactide-co-trimethylene carbonate) (PDLLA-co-TMC) scaffolds. Reprinted with permission of [226]. Copyright 2023, Springer Nature.

Despite the significant advances enabled by 3D printing, particularly in regenerative medicine and drug delivery [210], there remains a critical need for more dynamic constructs that can not only better mimic the complex, adaptive nature of living systems but also deliver therapeutics in response to specific stimuli at targeted sites. In this context, 4D fabrication offers a groundbreaking approach by enabling the creation of complex structures that also respond and adapt to external stimuli [211]. In 2013, Tibbits introduced the concept of 4D printing, describing it as ‘3D printing plus time as the fourth dimension’ [212]. This innovative technique is broadly defined as the fabrication of 3D printed structures capable of self-transformation in shape, property, and function when exposed to specific stimuli [213] such as heat [214], pH [215], light [216] electric and magnetic fields [217], and mechanical forces [218]. Unlike conventional 3D printed devices, 4D printed structures interact dynamically with their surroundings, responding to these stimuli with various outputs, such as mechanical movements or biological reactions. The 4D bioprinting process advances this approach by embedding stimuli-responsive biomaterials into 3D bioprinting, creating biologically active structures that can morph in response to specific triggers, thereby achieving targeted functionality (Fig. 6a–c) [219,220]. The ability of printed objects to change their shape, properties, or function over time offers great potential for meeting the real-time, adaptive needs of complex tissue and organ environments [221,222]. Specifically, skin tissue engineering can benefit from 4D printing structural complexity and responsiveness for the realization of dynamic tissue architecture that could better integrate within and recapitulate the dynamic physiological environment [223]. In a similar context, 4D printing fundamentally contributes to smart drug delivery by developing structures that controllably respond to specific physiological triggers/stimuli for the on-demand, targeted drug release [223]. While 4D printing of hydrogels is primarily designed to create constructs capable of dynamic adaptation, much of the research to date has focused on shape-morphing devices [224,225].

Stimuli-responsive hydrogels are central to the 4D printing process due to their ability to undergo controlled changes in physical and chemical properties in response to external stimuli such as temperature, pH, ion concentration, and electric or magnetic fields [227,228]. Shape memory hydrogels (SMHs) can adopt temporary configurations and return to their original shapes by forming or breaking reversible bonds in response to external triggers [229,230]. In SMHs, certain network segments remain permanent, while dynamic networks are employed to shape memory function [231]. However, incorporating reversible interactions into hydrogel systems to achieve shape-memory properties often compromises mechanical properties. To address this, various strategies have been employed, including developing double networks [232] and dual or triple crosslinking within a single hydrogel network [233] to fabricate more robust SMHs. Guo J [234]. developed a hydrogel system by in situ polymerization of acrylamide within an agarose matrix, incorporating laponite to improve shear-thinning behavior and shape stability. This dual network hydrogel, comprising thermal-reversible agarose nanofibers and lightly cross-linked polyacrylamide (PAM), confers strength, toughness, and dynamic shape transformation capabilities. Reversible sol–gel transitions enabled printed 3D structures to morph into different patterns. MTT assays revealed biocompatibility, with 90% cell viability after three days.

### 4.1 Synthetic stimuli-responsive hydrogels

Stimuli-responsive synthetic hydrogels, such as poly (N-isopropylamide) (PNIPAm) and poly (N, N-dimethylacrylamide) (PDMAAm), have been extensively studied for 4D bioprinting [235]. PNIPAm is especially notable for its thermoresponsive properties, undergoing a phase transition near physiological temperature. However, the limited mechanical strength and printability of such systems remain challenges. Goyal et al. [236]. Addressed these issues by designing a PNIPAm/alginate hydrogel ink reinforced with bio-sourced nanocellulose fibers (TCNF), which demonstrated anisotropic shape morphing when exposed to temperatures exceeding 36 °C. The TCNF-reinforced hydrogels demonstrated improved mechanical properties, achieving a tensile strength of 150 kPa, a Young's modulus of 6.77 MPa, and a toughness of 83 kJ/m^3^. Additionally, the printed hydrogels demonstrated superior drug release profiles compared to casted samples, underscoring their potential for antimicrobial applications. Deng et al. [237] developed a printable, light-curable magnetic hydrogel elastomer (PLMHE) with magnetic responsiveness, enabling rapid gelation and enhanced control. Bentonite was incorporated into the PLHME ink to improve thixotropic properties, increase viscosity and modulus, and prevent ferromagnetic particle aggregation by forming “house of cards” structures. An innovative approach integrated magnetization and programming directly into the printing process, eliminating the need for pre-magnetization.

### 4.2 Natural stimuli-responsive hydrogels

Naturally derived hydrogels are commonly preferred as cell carriers due to their inherent biocompatibility, water retention, and ability to support tissue formation [238]. 4D bioprinting of naturally derived hydrogels holds significant potential for creating dynamic structures for biomedical applications. However, challenges such as poor printability and surface roughness persist, especially when using micro-extrusion-based printers [239]. To address these challenges, Lai et al. [240] developed a hydrogel composed of alginate (Alg) and methylcellulose (MC) for 4D printing. The Alg/MC hydrogel demonstrated favorable rheological properties, extrudability, and high shape fidelity, enabling the precise fabrication of patterned 2D architecture with encoded anisotropic stiffness and swelling behaviors. By strategically controlling network density gradients perpendicular to the patterned strips, these 2D architectures transformed into prescribed simple and complex 3D morphologies after immersion in calcium chloride solution. ‌Building on the unique properties of alginate and methylcellulose, Siminksa-Stanny et al. [241] further developed this concept by incorporating polyacrylic acid-stabilized magnetite NPs (PAA-MNPs) into the hydrogel matrix, thereby creating patterned magnetic hydrogel actuators. This magnetic ink enabled the manufacturing of diverse 3D structures with macroscopically anisotropic magnetic properties, supporting steerable motion and dynamic responses, such as rolling, bending, and jumping under magnetic fields. The integration of magnetic and non-magnetic hydrogels within a single construct, combined with enhanced cytocompatibility, have underscored the potential of these innovations for biomedical applications.

Endogenous stimuli, such as pH and ROS, which are inherently dysregulated in chronic and infected wounds, are most appropriate for autonomous wound monitoring and regenerative activation. pH-responsive systems enable real-time visualization of infection status, while ROS-responsive hydrogels can trigger oxygen release or anti-inflammatory activity, as supported by several examples discussed above. For transdermal drug delivery in inflamed skin, temperature-responsive and ROS-responsive materials are more suitable. Local temperature elevation associated with inflammation naturally activates thermo-responsive systems (e.g., PNIPAm-based hydrogels), enabling on-demand drug release, whereas oxidative stress in inflamed tissues can be exploited for ROS-triggered therapeutic delivery. Conversely, exogenous stimuli such as light or magnetic fields are more appropriate when precise operator-controlled activation is required, including photodynamic therapy or programmable release.

## 5 5D printing

5D printing, also known as 5-axis 3D printing, represents an evolutionary advancement of 3D and 4D printing that was developed in 2016 by the Mitsubishi Electric Research Lab (MERL) team [242]. This technique involves an additive manufacturing process in which additional rotational axes are included for curved line deposition and hence more structural rigidity and functional versatility [243]. In simple-form 5D printing, the printing head moves along the typical three flat/planar axes (X, Y and Z), while the printing bed itself moves along two additional rotating axes (back and forth), enabling printing along curved paths and multidimensional, curved-layer deposition instead of the traditional flat-layer printing [244]. Such a dynamic range of motion allows for the fabrication of multidimensional, complex geometries with improved resolution and structural complexity, while simplifying the process and eliminating the need for support materials required in conventional 3D printing. Indeed, 5D printing consumes 20–30% less material than 3D printing for deposition of the same structure [244]. Moreover, 5D-printed structures demonstrate substantially higher structural rigidity (approximately 3.7 MPa) than their 3D-printed counterparts (0.1 MPa), according to MERL testing [245].

5D printing enables the fabrication of advanced tissue engineering constructs that integrate shape-morphing behavior with controlled delivery of biomolecular cues, supporting the regeneration of complex tissues such as multi-layered, cell-laden tubular structures (e.g., blood vessels). Fig. 6d schematically illustrates the 5D bioprinting of bilayer cell–scaffold constructs comprising a shape-morphing layer and an information-embedded layer containing rat bone marrow mesenchymal stem cells (rBMSCs) and biomolecule-delivery functionality [226]. The shape-morphing layer was 4D-printed using the shape-memory copolymer PDLLA-co-TMC, composed of poly (D,L-lactide) and trimethylene carbonate, with a glass transition temperature of ∼37 °C, enabling shape transformation under physiological conditions. The rBMSC-containing, biomolecule-delivering layer was fabricated using a dual-nozzle 3D printing system with two inks/bioinks. Upon integration into a bilayer scaffold, heating to 37 °C induced self-bending and self-folding into a tubular structure, as illustrated by the top and side views of large-diameter PDLLA-co-TMC scaffolds in Fig. 6d.

Briefly, the evolution of epidermal patch technologies reflects a shift from structural fabrication toward dynamic and multifunctional systems enabled by advances in additive manufacturing. 3D printing provides precise control over patch geometry, material composition, and drug distribution, enabling customized constructs with tunable mechanical and release properties. However, these systems remain largely static after fabrication**.** 4D printing introduces a temporal dimension through the incorporation of stimuli-responsive materials, allowing constructs to adapt their structure or therapeutic release in response to environmental cues such as temperature, electrical signals, moisture, or pH changes. Multidimensional or multi-axis (5D) printing has enabled the fabrication of complex curvilinear architectures with improved mechanical performance and anatomical conformity, supporting real-time sensing, guided responses, and closed-loop drug delivery**.**

Furthermore, the convergence of 4D and 5D printing technologies, sometimes referred to as 6D printing, holds promise for imparting additional stimuli-responsive functionalities to complex structures, thereby enhancing their functional versatility [244]. A major limitation of 5D printing lies in the technical complexity and the high cost of the specialized software and hardware required to accurately control the additional two rotational axes [246]. Nevertheless, this emerging platform—alongside 3D and 4D printing—holds substantial promise as a versatile and powerful manufacturing approach for engineering functional tissues and organ systems.

**Fig. 7:**
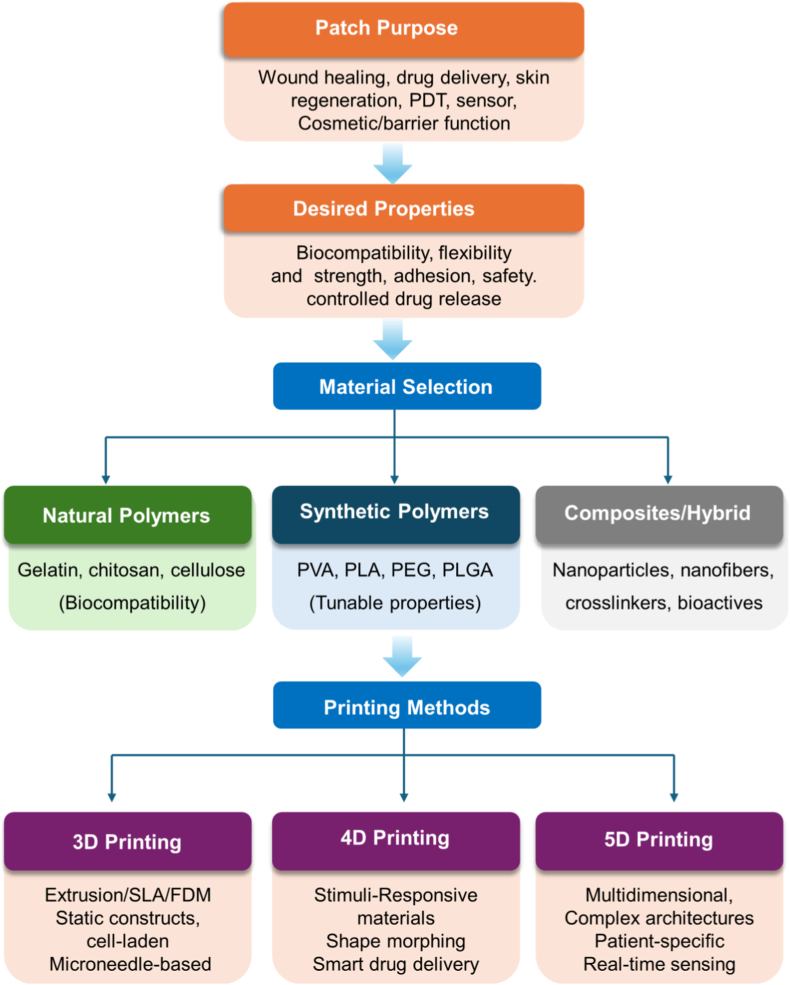
A schematic diagram illustrating a decision framework for selecting materials and techniques based on patch purpose.

The fabrication techniques described above enable the integration of diverse functional materials into flexible patch platforms. Fig. 7 presents a decision framework that connects patch purpose with suitable materials and compatible printing strategies, illustrating how design choices translate into practical device implementations. Guided by this perspective, the following section discusses key applications of patch-based systems.

## 6 Printed epidermal patches for biomedical innovation

While multidimensional printing techniques present promising avenues for the fabrication of personalized, dynamic constructs, the development of 4D- and 5D-printed epidermal patches for biomedical applications remains at an early stage. In contrast, 3D printing is currently the most mature and widely adopted additive manufacturing technology in healthcare, accounting for the majority of reported epidermal patch systems and clinical-oriented studies. Accordingly, this section primarily focuses on innovative biomedical applications of 3D-printed epidermal patches, encompassing both conventional (non-MAP) platforms and microneedle-based systems (MAPs), while selectively highlighting emerging examples of 4D printing to illustrate evolving trends and future opportunities.

Recent advances in materials engineering and printing dimensionality have driven a progressive expansion of skin-interfacing applications, reflecting a unifying shift toward functionally adaptive patch designs capable of precise skin conformity, spatiotemporal control of payload delivery, and responsive biological performance [247,248]. Within this integrated framework, drug delivery (dermal and transdermal), wound healing, and skin tissue engineering represent closely interconnected application domains, unified by shared requirements for mechanical compliance, controlled release of bioactive agents, and biocompatibility. These foundational functionalities are further extended to photodynamic therapy, biosensing, and cosmetic dermatology through the incorporation of stimuli-responsive materials, programmable architectures, and multifunctional design strategies. Collectively, these applications demonstrate that advances in 3D-printed epidermal patches do not constitute isolated technological developments, but rather an application-driven continuum that underpins personalized, multifunctional, and clinically relevant skin therapies.

### 6.1 Printed patches for dermal and transdermal drug delivery

This section examines both conventional (non-microneedle) epidermal patches and microneedle-based systems (MAPs) as complementary platforms for dermal and transdermal drug delivery, unified by shared design principles—such as biocompatibility, mechanical compliance, and controlled drug release—while differing in their modes and depths of skin interaction.

#### 6.1.1 Conventional patches

The application of 3D-printing for biomolecule delivery to the skin is an emerging and rapidly growing area of research. 3D-printing of conventional patches for dermal drug delivery have been the subject of many studies focusing on various printable inks made of polymer blends or composite systems. Examples include propranolol patches made of Eudragit RL for the treatment of cutaneous infantile haemangiomas [249], and an implantable procaine-loaded PCL and chitosan (CS) scaffold for local sustained post-surgical pain management [250]. Studies have shown that the formulation of topical corticosteroids in 3D-printed patches for treating inflammatory skin diseases, such as psoriasis and atopic dermatitis, improve their clinical use [251]. The patches enable customization of drug concentration, precise targeting of skin lesions, and controlled drug release rate. For example, extrusion-based 3D-printing was used to prepare patches containing blended vanillin-CS derivatives and ι-carrageenan incorporating fluticasone propionate [252]. Drug amorphization significantly enhanced *in vitro* drug delivery according to a desirable biphasic sustained release pattern. In another study [253], a bioadhesive patch was 3D-printed using a CMC/pectin blend, combined with clobetasol propionate-loaded mesoporous silica nanomaterials. Enhanced drug release was attributed to partial drug amorphization. Additionally, inkjet 3D-printing of dermal patches loaded with prednisolone nanosuspension in PLGA NPs demonstrated prolonged drug release, which reduces the frequency of application compared to conventional semisolid preparations [254].

Conventional 3D-printed skin patches can be engineered to incorporate biological materials either as bioinks or bioactive agents. For instance, a 3D-printed patch fabricated from a tissue-derived biomaterial ink was developed to deliver gentamicin and triamcinolone—an antibiotic and an immunosuppressant, respectively—in a spatially and temporally controlled manner [255]. This dual-delivery patch effectively reduced inflammation and minimized fibrous capsule formation around silicone implants in a rat model of breast reconstruction. In another study, acellular gelatin-based patches produced via extrusion-based 3D printing were designed with a controlled network topology and bioactivated using cell culture medium as a secretome model [256]. These constructs demonstrated the potential for personalized therapeutic applications, offering reproducible and lesion-specific pore architecture tailored to individual patient needs.

3D-printed conventional patches were also used for TDD, although improvements regarding material selection, structural design, skin adherence, efficacy, and safety are still needed [257,258]. Among recently reported 3D-printed TDD patches, a multicomponent HA/synthesized polylactone/methacrylate gelatin scaffold developed for the transdermal delivery of indomethacin exhibited release properties dependent on the relative ratios of its components [259]. Maurizii et al. [260] developed an ethylene vinyl acetate (EVA) copolymer-based transdermal patch fabricated by a direct powder extrusion method and medicated with ibuprofen and diclofenac sodium. The release and permeation profiles of both drugs were dependent on the EVA grade and VA content of the copolymer. In another study [261], a novel reservoir-type composite 3D-printed transdermal patch was developed using a Carbopol 934 gel matrix incorporating a supramolecularly structured zero-oxidation-state selenium Se [0]. This formulation demonstrated controlled drug release alongside pro-angiogenic properties, indicating strong potential as a TDDS for cutaneous wound healing with enhanced bioactivity and safety.

A more complex triple-layered transdermal patch was proposed for the local treatment of rheumatoid arthritis using a combination of electrospinning/electrospraying and 3D printing technologies [262]. A central composite layer, consisting of electrospun/electrosprayed polyvinyl alcohol NFs and NPs-conjugated diclofenac, was sandwiched between a supporting layer of electrospun PCL NFs and a 3D printed alginate-based hydrogel layer loaded with hyaluronate and rosuvastatin-loaded lipid nanocapsules (LNC). The patch released diclofenac via skin-secreted esterase enzymes at the inflamed sites, while rosuvastatin was almost completely released from LNC over 5 days. *In vitro* and *in vivo* evaluations confirmed the patch efficacy through a reduction in cell infiltration in the rats’ ankle joints with preservation of the joint tissue structure and alleviation of symptoms. Sharan et al. [263] developed a novel 3D-printed hydrogel patch designed as a subcutaneous (SC) implant for the sustained delivery of tenofovir for the treatment of hepatitis B. The implant, composed of a crosslinked polymer-reinforced bovine serum albumin (BSA) hydrogel, demonstrated favorable rheological properties, including shear-thinning and thixotropic behavior. Fabricated via semisolid extrusion-based 3D printing, the hydrogel formed a highly porous architecture that supported high cytocompatibility and enabled a sustained drug release profile. These characteristics make the system a promising candidate for the localized and prolonged treatment of chronic viral infections.

#### 6.1.2 Microneedle array patches

Although 3D-printed MAPs are mainly used for TDD [248,264,265], they have also been of considerable benefit to the intradermal delivery of drugs for treating local skin conditions. Upon application to the skin, MNs bypass the stratum corneum and painlessly access the microcirculation of deeper skin layers, enabling direct delivery of small molecules, proteins, and nucleic acids [16,266]. MAPs are actively investigated for their potential in personalized drug delivery and healthcare monitoring. In this context, hollow microneedles (HMNs) present a transformative approach for topical diagnostics and therapy, offering adjustable dosing and seamless integration with microfluidic and microelectronic systems to overcome conventional limitations [267].

***MAPs for intradermal drug delivery.*** Intradermal drug delivery based on 3D-printed MAPs has been the subject of some recent studies. For example, latticed MAPs (L-MAPs) fabricated using high-resolution 3D printing offered a tunable and versatile platform for intradermal drug delivery [268]. Modulating the L-MAPs with viscous coatings and combining different needle geometries on a single patch enabled the delivery of both liquid- and solid-state cargos and the tuning of cargo release into porcine skin, respectively. The L-MAPs potential was demonstrated using small drug molecules as well as protein and lipid NPs. In another study, 3D printed round MAPs (14 mm diameter) were manufactured using CLIP for the delivery of a novel oleogel-based betamethasone dipropionate formulation for psoriasis treatment [269]. The needle geometry varied from conical, square pyramidal, and obelisk and their length ranged from 400 to 1000 μm. The obelisk MAPs significantly increased the amount of corticosteroid penetrating the skin in a needle length-dependent manner. In a comprehensive study, hydrogel-forming microneedles made of Gantrez S97 and HA were fabricated using 3D printed masters for enhancing the dermal delivery of diclofenac sodium [270]. An optimized formulation, characterized by superior mechanical strength and enhanced drug accumulation in skin tissue, demonstrated greater efficacy in treating xylene-induced ear edema compared to a conventional preparation.

***MAPs for transdermal drug delivery.*** The field of TDD has been transformed by 3D printing, leading to numerous research reviews and patents for microneedle innovations [[271], [272], [273]]. Development of 3D-printed MAPs as a platform for minimally invasive biosensing/smart drug delivery [[274], [275], [276]], wearable technology for transdermal sensing/drug delivery [277], and skin permeation of bioactives for regenerative applications [278] have been the subject of recent literature reviews. Barnum et al. [279] introduced a simple and low cost strategy for the fabrication of 3D-printed hydrogel-filled MNs composed of a rigid outer layer, which was 3D-printed onto a conformal backing and filled with drug-loaded customizable hydrogels. The MAPs included MNs of different lengths or geometry on a single patch for the delivery of various agents to different tissue depths with controlled temporal release kinetics. Delivery of vascular endothelial growth factor (VEGF) verified the patch capabilities. In another study, SLA was used to produce six MN designs with three aspect ratios to enhance TDD [265]. MNs with a higher aspect ratio exhibited greater deformation characteristics, facilitating easier penetration to deeper skin layers. The MN width, which directly influences the force required for skin penetration, was reduced by using auxetic structures known for their negative Poisson's ratio in the design of MNs [280]. Upon skin insertion, compression of MNs causes a shrinkage in their radial dimension, unlike structures with positive Poisson's ratio, which expand. This shrinkage resulted in reduced penetration force.

In an attempt to reduce the gap between planar patches and contoured skin that affects effective drug delivery, Zhu et al. [281] have prepared a photothermal polymer doped with gold NPs (AuNPs) for high-fidelity 3D printing of customizable transdermal MAPs. The irradiation-induced photothermal heating triggers heat release by AuNPs, leading to sweat production. The sodium ions in sweat induces network rearrangements, resulting in curving of the patches to fit the body surface shape. The substantial increase in particle size of AuNPs promotes release sustainability. Other photo-printable inks comprising four functionally diverse monomers crosslinked by aluminum hydroxide NPs have been developed for DLP of high-precision, triple-responsive nanocomposite hydrogel MNs with exceptional mechanical strength [282]. Transdermal delivery of BSA provides a proof of concept for the triple sensitivity of the hydrogel MNs to pH, temperature and glucose levels, enabling more precise on-demand drug delivery. Another advancement involves the fabrication of stretchable MAPs through a single-step DLP process for transdermal drug delivery (TDD), enabling them to adapt to the dynamic movements of the body [283]. Rigid arrays of pyramidal MNs, each featuring a pore on each side for optimizing drug loading, are attached to a flexible patch capable of withstanding 50 % strain. This patch maintains the release of rhodamine B through both artificial and rat skin for over 70 h.

***3D-printed MAPs for transdermal insulin delivery and diabetes management.*** 3D-printed MAPs offer a minimally invasive innovative TDD platform in diabetes care enabling precise, painless, and patient-friendly diabetes management [284]. Recent studies have focused on 3D-printed MAPs as innovative, more comfortable insulin delivery devices that can be personalized through glucose sensing (Table 2). Economidou et al. [285] developed SLA-printed microneedles (MNs) for transdermal insulin delivery using a biocompatible resin. Subsequently, inkjet printing was employed to deposit thin layers of insulin and sugar onto the surface of the 3D-printed MNs. In vivo evaluations in diabetic mice demonstrated fast insulin release with excellent glycemic control within 60 min, maintaining a steady state glucose level over 4 h, compared to traditional SC injections. Later on, the authors introduced an innovative device combining 3D-printing, MNs and microelectromechanical systems (MEMS) for personalized TDD [286]. Hollow MNs built by SLA were integrated into a MEMS designed to pump single strokes of defined volumes of liquids. Advanced imaging techniques using fluorescein dye allowed monitoring of liquid cargo distribution within mice tissue in real time, while *in vivo* testing using insulin solution revealed improved glycemic control in diabetic mice compared to the SC injection.

**Table 2:** 3D-printed microneedle array patches (MAPs) for the transdermal delivery of insulin.

MN type	Material(s)	Printing technique(s)	Glucose responsive	Main outcomes	References
Solid MNs inkjet print-coated with insulin/xylitol	Photopolymerizable biocompatible resin	Stereolithography	No	Fast *in vitro* release of insulin within 30 min in porcine skin	[287]
3D-P^a^ resin MNs coated with 3D-P insulin/sugar thin layers	Biocompatible photopolymer	Stereolithography and ink jet	No	Fast and excellent hypoglycemia control in diabetic mice combined with steady state glucose level over 4 h.	[285]
Hollow MNs by SLA 3D-P/MEMS^b^ for liquid delivery	Biocompatible photopolymer	Stereolithography	No	Liquid cargo distribution within mice tissue in real time and improved glycemic control in diabetic mice relative to SC^c^ injections.	[286]
Curved pyramid and syringe-like hollow MNs	Biocompatible resin	Liquid crystal display	No	Possible MN-shape dependence of insulin transport across full thickness human skin using Franz diffusion cells	[288]
Conical MNs dip-coated with insulin	Biocompatible resin polymer	Stereolithography	No	High efficiency insulin delivery producing a hypoglycemic effect similar to that of SC^c^ injections.	[289]
Conical MNs formed by stretching the patch top surface	Sodium alginate/hydroxyapatite	Extrusion	Yes	Regulation of blood glucose levels in diabetic mice within normal ranges for up to 40 h with alleviation of their diabetic symptoms	[290]
Conical shape	Biocompatible light-sensitive resin	Digital light processing	Yes	Continuous and real-time monitoring of subcutaneous glucose levels under the intake of food or insulin injection.	[291]
Hollow MNs coated with a printed sensor and integrated with an electroosmotic micropump and a printed control circuit board	Polystyrene and a graphene composite ink-printed sensor	Extrusion-based ink deposition	Yes	Excellent blood glucose control in diabetic rats via smart control of the sensor and pump to measure interstitial glucose level and deliver insulin through the MN channels.	[292]

a 3D-printed.

b Microelectromechanical systems.

c Subcutaneous.

Curved pyramid and syringe-like hollow MNs were fabricated using a liquid crystal display (LCD) vat polymerization method for TD of insulin [288]. The method enabled fast fabrication of highly complex objects at low cost. Diffusion studies across full thickness human skin using Franz diffusion cells revealed a potential dependence of insulin transport on the shape of MNs. Using a different approach, Anbazhagan and Suseela [289] developed 3D printed polymer conical MNs that were dip-coated with insulin solution and PVA. Following process optimization, *in vivo* studies in diabetic rats revealed consistent and efficient insulin delivery, providing a hypoglycemic effect similar to that of SC injections. For deeper and faster transdermal insulin delivery, Chen et al. [293] developed a lightweight and minimized 3D-printed MAP, employing a cymbal-type ultrasound transducer. Guided by finite element modeling, the optimized device achieved insulin delivery in diabetic mice comparable to intraperitoneal injection, showing strong potential for cutaneous and intraoral therapies. In another attempt, transdermal insulin delivery enhancement was achieved by employing insulin/GOx-loaded ZIF-8 MNs [248]. A key innovation is the use of a polymerizable deep eutectic solvent (PDES)-based ink for DLP 3D printing, enabling rapid photopolymerization and precise microneedle fabrication. The developed MAPs resulted in effective insulin delivery, stability, and controlled kinetics as verified by *in vitro* and *in vivo* data.

Despite potential advantages of 3D-printed MAPs for insulin delivery, integrating a glucose-responsive component into the patches generates glucose sensitive 3D-MAPs (GSMAPs) as closed-loop devices capable of continuous glucose monitoring (CGM) and modulation of insulin release. So far, two main types of GSMAPs systems based on glucose oxidase and phenylboronic acid have been developed [294,295]. Razzaghi et al. [275] reported a theragnostic GSMAP of hollow MNs using an array of colorimetric sensors for quantitative measurement of pH, glucose, and lactate. The system integrated an ultrasonic atomizer and an on-demand, remotely triggering insulin delivery mechanism. It was also paired with a smartphone application for interfacing the sensing and drug delivery components, offering solutions for long term drug delivery challenges in the remote management of chronic conditions, including diabetes.

Although several studies involving glucose-responsive TD of insulin based on conventional MNs have been recently reported [275,296,297], 3D printed MAPs technology has not yet gained its full potential in developing diagnostic/therapeutic devices. This domain is challenged by integrated technologies for continuous glucose monitoring, intelligent control algorithms, personalized insulin release, alongside with regulatory constraints [298]. These constrains may explain the limited number of publications showcasing smart, and wearable insulin closed-loop TDDSs. Recently, a minimally invasive alginate/hydroxyapatite GSMAP was fabricated by extrusion 3D-printing [290]. Conical glucose-responsive insulin-loaded MN-like tips were formed by stretching the top surface of a cylindrical array of the patch. By releasing insulin in response to the glucose levels in type 1 diabetic mice, the patch enabled fast insulin release within 60 min with excellent hypoglycemic control for up to 40 h. Liu et al. [291] fabricated an integrated MN-based biosensing device for insulin delivery using 3D printing, microfabrication, electroplating, and enzyme immobilization processes. The device displayed accurate SC glucose level sensing performance. More recently, the authors [292] developed a rapidly manufacturable wearable MN patch for closed-loop diabetes management. The patch was made of hollow MNs integrated with a graphene composite ink-printed sensor, a PEG-functionalized electroosmotic micropump, and a printed circuit smart control board. This patch enabled the measurement of interstitial glucose level and the responsive delivery of insulin through the MN channels. It demonstrated excellent blood glucose control in diabetic rats. In a recent study, Liu et al., [299] introduced a sustainable, multifunctional 3D-printed dissolving MAP integrating a polymerizable deep eutectic solvent (PDES) matrix with a recyclable eutectogel backing. The biocompatible construct enabled rapid photopolymerization, effective skin penetration, and glucose-responsive insulin release, achieving significant glycemic control in diabetic rats. The conductive, adhesive backing supports wound healing, wearable sensing, and real-time movement monitoring. The system accelerated wound closure and minimized inflammation in diabetic mice.

***Transdermal delivery of other biomolecules.*** Beyond insulin delivery for diabetes management, 3D-printed MAPS have recently been used for the transdermal delivery of other drugs and biomolecules, offering potential treatments for a range of conditions. Table 3 summarizes recent studies on this topic. Among reported drug delivery MAPs, reservoir-based 3D printed estradiol valerate MAPs, fabricated using PLA and a combination of FDM 3D printing and injection volume filling, enabled painless and prolonged estradiol transdermal drug delivery for up to 7 days [300]. Bagde et al. [197] designed dissolvable AI-optimized DLP-printed MAPs for the transdermal delivery of lipophilic drugs. Ibuprofen was selected as a model drug, and skin permeation experiments revealed sustained permeation over 72 h. Pharmacokinetic (PK) studies in rats indicated a polynomial relationship between the release of ibuprofen and its fraction absorbed *in vivo*. The authors also developed a 3D DLP-printed MAPs for enhanced cannabinoid bioavailability [301]. An *ex vivo* permeation study showed that skin permeation of the drug from a 3D-printed MAP was enhanced compared to a 3D-printed conventional patch. PK assays also demonstrated higher drug bioavailability compared to SC injection.

**Table 3:** 3D-printed microneedle array patches (MAPs) for the transdermal delivery of various drugs.

Drug	MN or MAP type	Material (s)	Printing technique(s)	Main outcomes	References
Estradiol valerate	Reservoir-based MAPs	Polylactic acid	Combined FDM and injection volume filling techniques	Painless skin penetration and prolonged TDD for up to 7 days.	[300]
Ibuprofen	Dissolvable MAPs	LAP^a^, PEGDAMA 550^b^ and photoinitiator	AI-optimized digital light processing	Sustained skin permeation at 72 h, biphasic rapid first-order drug absorption with sustained zero-order input in pharmacokinetic studies in rats with an in vitro-in vivo polynomial relationship	[197]
Cannabinoid	Sharp MNs with a tip with a radius of curvature (RoC) of ∼15 μm	LAP^a^ and PEGDAMA 550^b^	Digital light processing	Enhanced e*x vivo* skin permeation and *in vivo* bioavailability compared with a conventional 3D-printed patch and subcutaneous injection, respectively	[301]
Dexametha-sone	Bioresorbable MAPs	Poly (propyl-ene fumarate-co-propylene succinate) oligomers	Continuous liquid interface production	Considerable relief of postoperative pain at a significantly lower dose than intravenous injection in a murine tibial fracture model	[302]
Ceftrioxone sodium	Hollow MNs (HMNs) with bio-inspired labrum tip	Photo-crosslinkable and curable resin	Vat-photopolymerization mediated stereolithography	The reservoir HMNs patch allowed 100% *ex vivo* drug permeation through porcine skin in 18 h as well as efficient *in vivo* skin penetrability and bioavailability in an animal model	[303]
Melatonin	Dual function pyramidal MAPs for ISF^c^ aspiration and hormone delivery	PEGD^d^	Masked stereolithography	Potential of melatonin loaded MAPs to deliver and collect ISF for melatonin analysis.	[304]
Denosumab (Dmab) monoclonal antibody	Hollow MNs with durable mechanical properties and piercing capacity	Biobased photocurable resin	Stereolithography printing combined with a microelectron-mechanical system	Enhancement of therapeutic efficacy manifested as restoration of the serum levels of bone minerals in osteoporotic rats compared to SC^a^ injections	[305]
Imiquimod nanocrystals	Dissolving MNs	PEGD^d^ + NVP^e^ + LAP^a^ as photo- initiator	Digital light processing	Imiquimod as nanocrystals was more homogeneously distributed in the printing achieving 48% increase in release from MAPs *ex vivo* in natural skin compared to its original form.	[306]
Lidocaine	Solid MNs Combined with nanostructured lipid carriers (NLC)	High Temp V2 resin	Stereolithography	The 3D-P solid MNs/lidocaine NLC combination synergistically improved *in vitro* drug delivery through human epidermis but not deeper skin layers.	[307]
Rivastigmine and N-acetyl-cysteine	Solid MNs coated with drug-loaded PLGA^f^ NPs^g^	Biocompatible Class I resin	Digital light processing	Simultaneous release of the 2 drugs into skin *ex vivo* without considerably affecting the stratum corneum integrity	[308]
Donepezil	Solid conical MNs coated with a drug/HPMC^h^ film	Biocompatible Class I Dental SG resin	Digital light processing/semisolid extrusion	Significantly increased drug permeation compared to plain coating material, as well as distribution within skin layers, sustained release and transcellular transport demonstrated by CLSM.	[309]
Gentamicin	Solid MNs coated with a drug/PVA/sucrose film	Anycubic UV resin	Digital light processing	*In vitro* data indicated ultimate drug release within 312 h.	[310]

a LAP: Lithium phenyl (2,4,6-trimethylbenzoyl) phosphinate.

b PEGDAMA 550: Polyethylene glycol dimethacrylate 550.

c ISF: interstitial fluid.

d PEGD: polyethylene glycol diacrylate.

e NVP: N-Vinylpyrrolidone.

f PLGA: polylactic-co-glycolic acid.

g NPs: nanoparticles.

h HPMC: hydroxypropyl methylcellulose.

To achieve high local concentrations while maintaining low plasma levels of dexamethasone (DXM), Bahnick et al. [302] incorporated the drug in photochemically 3D-printed bioresorbable MAPs for controlled transdermal delivery. Poly (propylene fumarate-co-propylene succinate) oligomers were CLIP 3D printed into DXM-loaded MAPs. Application of the MAPs in a murine tibial fracture model showed considerable relief of postoperative pain at a significantly lower dose than intravenous injection. Moreover, a 3D printed patch of hollow MNs (HMNs) was used for the transdermal delivery of ceftriaxone, an antibiotic with gastrointestinal instability and low oral bioavailability [303]. The HMNs featured a bioinspired labrum tip to reduce insertion force. The patch was fabricated using a vat-photopolymerization/SLA assisted 3D printing technique employing a biocompatible photo-crosslinked curable resin. The ceftriaxone-reservoir HMNs patch allowed 100% *ex-vivo* drug permeation through porcine skin in 18 h and achieved the required bioavailability in an animal model. In a recent study, 3D-printed (MAPs) were developed for the simultaneous delivery and monitoring of melatonin levels in interstitial fluid (ISF) [304]. A polyethylene glycol diacrylate patch featuring pyramidal microneedles with robust mechanical properties was fabricated using an optimized masked stereolithography (mSLA) 3D printing technique. Two types of MAPs were produced: one designed for ISF collection (aspiration) and the other loaded with melatonin for transdermal delivery. Simultaneous application of both patches in a rat model enabled real-time monitoring and localized hormone administration.

Drug encapsulation into biodegradable nanocarriers is a different strategy for drug skin permeation enhancement achieved by 3D printed MAPs. As demonstrated recently, skin permeation of lidocaine, a drug used for local anesthesia and the management of inflammation and pain, was enhanced by dual passive and active permeation-enhancing strategies based on drug loading into a nanostructured lipid carrier (NLC) and 3D-printed solid MNs, respectively [307]. The 3D printed solid MNs/lidocaine NLC combination provided an innovative and synergistic approach to improving *ex-vivo* lidocaine delivery through the human epidermis but not deeper skin layers. Using a similar approach, Monou et al. [308] coated electrosprayed PLGA NPs encapsulating rivastigmine (RIV) and N-acetylcysteine (NAC) onto solid MNs for treating Alzheimer's disease. The coated MNAs fabricated by DLP printing simultaneously released the two drugs into the skin *ex vivo* without considerably affecting the stratum corneum integrity. Another approach for Alzheimer's disease involved the fabrication of solid MNs hybrid coated with a drug-containing film [309]. Solid MNs fabricated by DLP were coated with a hydroxypropylmethylcellulose (HPMC) solution of donepezil using a semisolid extrusion (SSE) method. Skin permeation studies indicated a significant increase in donepezil permeation compared to plain coating material. Confocal laser scanning microscopy (CLSM) verified drug distribution within skin layers, demonstrating sustained release and transcellular transport pathways. A simpler MN coating technique was used by Mutlu et al. [310] to fabricate gentamicin-coated 3D printed MAPs. Resin-based MAPs were prepared by a DLP technique and the MNs were dip-coated with a solution of gentamicin sulfate containing PVA and sucrose. *In vitro* studies indicated complete drug release within 312 h. Overall, 3D printed epidermal patches offer great promise for the drug delivery to skin, providing innovative, patient-friendly treatment options with enhanced efficacy and safety.

Among the limited reports on 4D-printed hydrogels for drug delivery, Regato-Herbella et al. [311] developed triple-responsive hydrogels incorporating ketoprofen for the treatment of inflammatory conditions. These hydrogels were synthesized via UV-induced photopolymerization of thermoresponsive N-isopropylacrylamide, pH-responsive methacrylic acid, and a tailor-made ROS-responsive diacrylate thioether monomer. The resulting smart hydrogels were amenable to fabrication via DLP 4D printing. Their responsiveness to temperature, pH, and reactive oxygen species (ROS) was assessed through swelling and rheological tests under various conditions. These hydrogels enabled stimuli-responsive drug release and demonstrated inherent anti-inflammatory activity *in vitro*. In another study, Regato-Herbella et al. [312] developed ROS-responsive 4D printed acrylic thioether-based hydrogels for smart drug release using 5-fluorouracil as a model drug. Thioether-based difunctional monomers (EGnSA, n = 1–3) were synthesized via thiol–Michael addition from ethylene glycol/thioether acrylates. Acrylate groups enabled UV-induced photopolymerization, while thioether moieties conferred ROS sensitivity. Hydrogels with improved post-swelling stability and aqueous compatibility were processed via 4D printing demonstrated significant H_2_O_2_-triggered swelling (∼130%), confirming their ROS-responsiveness. The hydrogels enabled sustained, ROS-modulated release of 5-FU. In vitro studies with B16F10 melanoma cells showed notable growth inhibition, underscoring their potential for localized, stimuli-responsive cancer therapy.

Moreover, Zhang et al. [313] reported a 4D printing strategy using plant protein (zein) gel inspired by amyloid fibril formation. Zein was printed in a layered Carbopol support bath with varying water concentrations in ethanol–water mixtures, inducing temporal functional changes via modulated hydrophobic and hydrogen bonding. Constructs printed in higher water content showed increased drug loading, faster release, and faster degradation. This approach expands 4D printing beyond shape transformation by enabling spatial and temporal functional tuning of drug delivery and other biomedical applications. Using a different approach, Goyal et al. [236], developed a nanocellulose-reinforced 4D printed hydrogels with thermoresponsive shape morphing for controlled drug release. A Poly (N-isopropylacrylamide) (PNIPAM)/alginate ink reinforced with TEMPO-oxidized cellulose nanofibers (TCNF) enabled anisotropic shape morphing above 36 °C. Shear-induced fibril alignment during direct ink writing, followed by ionic and photo-crosslinking, enhanced shape fidelity in bilayer structures. The TCNF-reinforced hydrogels demonstrated controlled drug release and antimicrobial potential compared to casted ones, holding promise for biomedical applications.

Notably, MAPs designed for dermal or transdermal drug delivery share key mechanistic and engineering principles with those developed for minimally invasive diagnostics. The same micro-projections and printed architectures that enhance drug permeation, by generating transient microchannels and ensuring intimate epidermal–dermal contact, also provide direct access to interstitial fluid (ISF), a biofluid increasingly recognized as a reliable surrogate for blood-based monitoring [314]. Diagnostic MAPs leverage this ready access by integrating ISF-extracting microneedle arrays (MNA) with biological recognition elements (e.g., enzymes, antibodies) and electrochemical transducers to enable rapid, sensitive detection of biomarkers such as glucose, lactate, electrolytes, and cancer biomarkers [315,316]. The evolution of these MN-integrated sensors broadens their utility from mere drug delivery to complex sensing and therapeutic applications, supporting closed-loop management from detection to therapy, including insulin delivery for diabetes management [317].

Advances in additive manufacturing and printed electronics have enhanced the efficiency of ISF sampling, enabling continuous blood glucose monitoring [291,318]. These developments have enabled the integration of microfluidic channels, biochemical recognition layers, and flexible electrochemical sensors directly into patch substrates, supporting bidirectional operation in which therapeutics are delivered while physiological signals are continuously monitored. A recent review highlighted the promise of MAPs for advanced diabetes management, emphasizing their capacity to monitor multiple biomarkers beyond glucose, autonomously deliver insulin and glucagon via glucose-responsive materials, and integrate diagnostic and therapeutic functions within a single MN platform [319]. Beyond glucose regulation, Chauhan and Venuganti [304] optimized masked stereolithography 3D printing to fabricate PEGDA MAPs capable of both melatonin delivery and ISF sampling. The printed pyramidal MNs aspirated ISF in rats for circadian melatonin monitoring, while the simultaneous use of melatonin-loaded and blank ISF-collection patches demonstrated a dual-function platform for hormone delivery and minimally invasive ISF analysis.

3D-printed theragnostic MAPs are emerging as adaptable platforms capable of real-time biomarker monitoring and precision drug delivery across a wide range of clinical applications. Although diabetes remains the primary focus, propelling the development of AI-guided, MN-based fully integrated closed-loop “sense–release” artificial pancreas systems that optimize glycemic control, these technologies also hold substantial promise for broader therapeutic use, including chronic inflammatory disorders, infectious diseases, and cancer.

### 6.2 Cutaneous wound healing

Cutaneous wound healing is a multifaceted, precisely coordinated process that safeguards the efficient restoration of tissue architecture and preservation of the skin barrier function [320]. The process comprises overlapping phases of hemostasis, inflammation, proliferation, neovascularization, and remodeling synchronized by a complex interplay of cellular and molecular mechanisms [320]. While acute wounds follow a relatively short path toward closure, healing may be delayed in case of surgical or traumatic injuries and diabetic wounds, necessitating effective personalized wound care solutions [321,322]. A wide range of product formats—such as films, foams, hydrogels, electrospun nanofibers, smart dressings, pads, and patches, including conventional, microneedle-based, electronic, and hybrid/composite types—have been explored for wound care applications [[323], [324], [325]]. Among these, 3D printing technology stands out for its ability to precisely tailor the design, composition, and functionality of both conventional and MN-based patches, thereby enhancing the efficiency of wound closure and healing outcomes. Building on their established role in dermal and transdermal drug delivery, 3D-printed epidermal patches of both conventional and MAP-based types have emerged as versatile platforms for addressing the challenges of wound management. The same design principles that enable precise and localized drug administration, including mechanical compliance, biocompatibility, and tunable release kinetics, are equally critical for promoting tissue repair and maintaining an optimal wound microenvironment. Multifunctional patch architectures, particularly those enhanced through 4D printing, can deliver growth factors, antimicrobial agents, or other bioactive molecules in a spatiotemporally controlled manner, while conforming to complex wound geometries and adapting to dynamic tissue changes [326]. Collectively, these technological advances in patch design and printing not only support targeted therapeutic delivery but also actively promote wound healing, illustrating a rather an integrated, application-driven framework in which material innovation, dimensional printing, and functional design converge to enable personalized and clinically relevant skin therapies.

#### 6.2.1 3D-printed conventional patches

3D-printed conventional epidermal patches and scaffolds have emerged as a highly versatile wound-care platform, offering unprecedented levels of customization and precision [327,328]. Their architectures can range from simple geometries to complex multifunctional constructs depending on the ink composition, printing technique, and design strategy [329]. Functionalization with antimicrobial agents, antioxidants, immunomodulators, and growth factors further enhances their regenerative performance [[330], [331], [332], [333]]. Moreover, integrating nanoparticles and microparticles [165], cells [334], natural bioactive healing promotors [335,336], exosomes [337] or platelet rich plasma [338] confers more potent wound-healing capabilities.

Within the scope of relatively simple 3D-printed geometries, Hu et al. [339] fabricated an adhesive, flexible wound patch based on dopamine, PEDA, and NVP using DLP printing, which performed effectively in a challenging *in vivo* model. Likewise, a 3D-printed amphiphilic chitosan graft copolymer blended with elastin, collagen, and gelatin and loaded with levofloxacin showed strong wound-healing and anti-infective potential [330]. Patil et al. [333] further introduced a biocompatible, dual-crosslinked multifunctional hydrogel patch made of glycol-functionalized chitosan reinforced with zinc and tannic acid, which demonstrated potent antibacterial and immunomodulatory effects with enhanced wound healing in a rat model.

As the field advances, 3D printing is increasingly being leveraged to create complex constructs with stratified or gradient architectures that mimic the epidermis and dermis, along with precise control of porosity to regulate moisture, gas exchange, and cellular infiltration. Functionally, these patches enhance therapeutic efficacy and accelerate wound repair by combining biomimetic structure and programmable delivery of bioactive agents, antibacterial functions, cell deposition, and bioelectric stimulation [340]. In this context, a bioinspired 3D-printed bilayer patch comprising a dense HA/CS hydrogel top layer and a nanofibrous microsphere-based bottom layer spatially loaded with DDAB-modified nano-ZnO markedly enhanced healing of infected diabetic wounds in rats, achieving ∼95% closure and full skin-layer regeneration within 14 days [165]. The modular bilayer design, tunable porosity, and spatially confined bioactive loading markedly improved antibacterial performance and tissue regeneration. Similarly, a 3D-printed double-layer hydrogel system featuring methacrylated silk fibroin/GelMA as an epidermal-mimetic top layer and GelMA/HAMA loaded with copper-epigallocatechin gallate as the dermal layer, promoted scarless burn repair by enhancing angiogenesis, maintaining epidermal hydration, and mitigating fibrosis [341]. To better replicate the skin hierarchical structure, Koupai et al. [342] developed a multifunctional tri-layer wound dressing. A 3D-printed alginate-tragacanth-ZnO nanoparticle layer was positioned between a hydrophobic PCL upper layer mimicking the epidermis and a Soluplus®/IGF-1 lower layer promoting cell activity. The dressing showed strong antibacterial and pro-regenerative effects through ZnO and IGF-1 release and markedly accelerated full-thickness wound healing in rats compared with mono- and bilayer designs.

Beyond structural design, the precise spatial loading of bioactive agents and the controlled temporal release enabled by 3D-printing provide therapeutic advantages that conventional dressings cannot achieve. For instance, Hu et al. [343] developed a multilayer 3D-printed temporospatial hierarchical patch that mimics native skin architecture and sequentially delivers cell-modulating cues aligned with the natural stages of wound repair. By directing the transition from the pro-healing to the pro-remodeling phase, specifically the shift from the M2a to the M2c macrophage sub-phenotype, the patch promoted more mature tissue formation and improved overall functional regeneration. On the other hand, integrating 3D-printing with bioelectric stimulation (BES) enables the fabrication of wound patches that combine electrical functionality with tailored geometry, controlled porosity, spatially defined drug reservoirs, and precise sensor/electrode placement, thereby improving the rate and quality of chronic and acute wound healing [344]. Bioelectric stimulation (BES) refers to the application or restoration of electrical cues at the wound site to mimic or enhance the endogenous electric fields generated after injury. These signals regulate essential cellular processes, including galvanotactic migration, proliferation, differentiation, angiogenesis, and ECM deposition, while exerting antibacterial effects and modulating immune responses [345]. Multifunctional BES-activated 3D-printed patches integrate conductive inks or hydrogels [346], conductive patterned patches or electrodes [347,348], and piezoelectric or triboelectric generators [349] to provide spatially targeted, temporally controlled electrical stimulation while simultaneously delivering bioactive agents. Chai et al. [350] reported a 3D-printed conductive composite wound patch fabricated from a poly (vinyl alcohol)/κ-carrageenan bioink incorporating the conductive polymer complex poly (3,4-ethylenedioxythiophene):polystyrene sulfonate (PEDOT: PSS) and catechin-loaded mesoporous ZnO (CmZnO). The construct exhibited appropriate conductivity, strong adhesiveness, rapid hemostasis, and potent antibacterial activity. When combined with optimized electrical stimulation, the dressing accelerated full-thickness wound repair *in vivo* by enhancing angiogenic gene expression (CD31) and suppressing inflammatory markers (IL-6). Likewise, Kumi et al. [351] described a 3D-printed flexible porous electrode composed of quaternized chitosan (QCS) and PEDOT:PSS, custom-shaped to conform to wound contours and improve the electrode-wound contact. Under electrical stimulation, this electrode promotes healing of infected diabetic wounds, underscoring the advantages of 3D-printing in enabling tailored electrode designs and improved signal distribution.

Integrating 3D printing with complementary technologies, such as electrospinning, microfluidics, and biosensing, further enhances the physicochemical and biological performance of wound-care patches. Hybrid electrospinning-3D printing systems are particularly promising, offering customizable porosity, improved mechanical stability, and tunable drug release [352]. Microfluidic-assisted 3D printing has also emerged as an approach to highly ordered fibrous constructs [353]. For instance, Guo et al. [354] developed a wound-healing scaffold composed of microfluidically 3D-printed poly (3-hydroxybutyrate-4-hydroxybutyrate) [P (3HB-co-4HB)] and PCL, featuring a hierarchical porous architecture. Loaded with two types of stem cells, this biomimetic construct markedly enhanced re-epithelialization, collagen deposition, and angiogenesis in a rat wound model. Moreover, Dong et al. [355] developed a dual-function asymmetric tri-layer skin patch composed of polyurethane and bioactive glass using microfluidic-regulated 3D bioprinting. When integrated with electronic skin (e-skin), the patch exhibited enhanced hemostatic, antibacterial, and pro-angiogenic properties, leading to improved wound healing.

Despite notable progress, most 3D-printed wound patches still lack real-time monitoring capabilities, which can be addressed by integrating sensing technologies. Tsegay et al. [356] used DLP printing to create smart auxetic hydrogel dressings with paper-based colorimetric sensors for detecting changes in pH and glucose level. Analogously, Guo et al. [357] developed a wearable PVA/sucrose hydrogel patch incorporating a pH-responsive photoacoustic probe to monitor exudate pH (5.0–9.0) without interference from blood or drugs. To improve conformal adhesion, Ma et al**.** [167] designed a DLP-printed diagnostic–therapeutic patch inspired by octopus suckers and snail mucus, combining bioinspired adhesion with ultrasound-enhanced toughness. The patch exhibited strong mechanical and electronic performance, antibacterial and photothermal activity in addition to effective wound-healing and sensing functions.

Beyond 3D printing, 4D-printed patches are emerging for advanced wound healing. Lu et al. [358] developed a DLP-printed, adhesive, thermo-contractile, and degradable hydrogel patch for diabetic wounds, comprising NIPAm, curcumin-loaded Pluronic F127 micelles, and PEGDA575-dopamine. Thermo-responsive NIPAm chains enable body temperature-induced wound contraction, while the patch's strong adhesion, antibacterial activity, hemostasis, biodegradability, and inflammation regulation accelerated healing in MRSA-infected diabetic skin, highlighting its potential for complex wound care. Moreover, Jensen et al. [359] reported a 4D-printed, high-swelling composite hydrogel of GelMA and sodium polyacrylate (SPA) for wound healing, tissue regeneration, and drug delivery. The hydrogel patch exhibited >500% area expansion and a 100-fold water weight increase, surpassing existing 4D bioprinting materials. It enables cyclical, on-demand swelling and shrinking in response to ionic strength, while providing superior cytocompatibility, cell support, and print fidelity, highlighting its potential for dynamic biomedical applications.

#### 6.2.2 3D-printed microneedle patches

Various types of MAPs functionalized with diverse bioactive agents have been developed to accelerate wound healing [[360], [361], [362], [363]]. However, the application of 3D printing in MAP fabrication provides additional advantages, including enhanced geometric flexibility, multifunctionality, and improved mechanical performance. For example, extrusion-based 3D printing of MXene- and spidroin-laden MAPs has demonstrated multifunctionality, including self-healing, NIR responsiveness, and high drug-loading capacity [364]. In vivo studies using full-thickness wound models in rats showed enhanced healing under NIR irradiation. In a related approach, MAPs with polyurethane needle tips and a biocompatible backing of spidroin and aloe vera gel were fabricated [365]. Incorporating eutectic gallium–indium and a photothermal polymer enabled NIR-triggered pulsed VEGF release, significantly accelerating wound closure in mice.

An innovative bioinspired indwelling MN system was developed for diabetic wound healing, featuring therapeutic exosome-loaded polyvinyl alcohol (PVA) tips attached to a detachable medical tape substrate [366]. This system was fabricated using a combination of template replication and 3D transfer printing. To optimize mechanical performance, sulfate and nitrate ions were incorporated into the PVA tips, enhancing tip hardness for effective skin penetration and promoting softening after detachment to facilitate exosome release. The indwelling MNs significantly accelerated wound healing and tissue regeneration in a rat model of full-thickness diabetic cutaneous wounds. In a separate study, Wang et al. [367] engineered bioinspired microfluidic and 3D-printed multifunctional origami MAPs with superfine microneedle structures and integrated microfluidic channels. These devices demonstrated capabilities in biomarker detection, controlled drug release, and motion monitoring, collectively contributing to improved wound healing outcomes. The MAP substrate was designed to be porous and incorporated a thermosensitive N-isopropylacrylamide (NIPAM) hydrogel and polydopamine, which served as a photothermal-responsive agent. To enable motion sensing capabilities, MXene-based electro-circuits were printed onto the MAPs. *In vivo* assessments using a mouse model of full-thickness cutaneous wounds demonstrated that the multifunctional patch significantly accelerated wound healing.

The development of 4D-printed patches for wound healing has gained momentum in recent years. For example, Lu et al. [358] developed a 4D-printed, adhesive, thermo-contractile, and degradable hydrogel patch for diabetic wound healing. The dressing conforms to wounds with complex shapes and depths, promoting closure and tissue regeneration. Fabricated via DLP printing, the hydrogel comprised N-isopropylacrylamide (NIPAm), curcumin-loaded Pluronic F127 micelles, and PEGDA575-dopamine as a degradable crosslinker. The thermo-responsive NIPAm chains enable body temperature–induced wound contraction. Key features, including strong adhesion, temperature responsiveness, antibacterial activity, hemostasis, biodegradability, and inflammation regulation, contributed to accelerated healing in MRSA-infected diabetic skin and liver injury models, demonstrating the patch's potential in advanced wound care.

In another study, a 4D-printed, high-swelling composite hydrogel composed of gelatin methacryloyl (GelMA) and sodium polyacrylate (SPA) was developed for wound healing, tissue regeneration and drug delivery applications [359]. The hydrogel exhibited over 500% area expansion and a 100-fold increase in water weight, outperforming existing 4D bioprinting materials. It enables cyclic, on-demand swelling and shrinking in response to ionic strength changes, and offers superior cytocompatibility, cell support, and print fidelity compared to SPA alone. These features highlight its potential for advanced 4D printing and dynamic biomedical applications.

In a recent review, Ni et al. [27] explored advanced technologies—3D/4D printing, network pharmacology, and multiomics—for personalizing traditional Chinese medicine gels to enhance their performance in wound healing. These multicomponent, multitarget gels show strong potential for addressing key challenges in the wound healing process, including inflammation, angiogenesis disorders and microbial infections.

Although MAPs, including 3D-printed variants, offer considerable potential for minimally invasive drug delivery and diagnostics, their clinical translation is limited by several interrelated challenges. MN performance is highly sensitive to material composition, fabrication technique, needle geometry, density, and tip sharpness, as well as application variables such as insertion mode, consistency, duration, and repeatability [368,369]. Drug-loading capacity remains inherently limited by MN dimensions, restricting MAPs to low-dose therapeutics that do not require repeated administration [370]. Additionally, patient-specific skin characteristics, including anatomical site, thickness, hydration, mechanical properties, and barrier integrity, further impact insertion efficiency, drug permeation, and overall dosing consistency [371,372]. Moreover, scalability and standardization remain major hurdles, particularly regarding uniformity of microneedle geometry, mechanical strength, and reproducibility of drug loading across large batches [373,374]. Regulatory approval is similarly challenging, as many MAPs function as combination products, necessitating evidence for both device and drug components and prolonging approval timelines [375]. Recently, a white paper provided a platform for the development of dosage form specific guidance that expedites clinical translation of safe and effective microneedle-based products [375].

The integration of 3D printing, while enabling precise architectural control, introduces compromises among printability, biocompatibility, drug-loading efficiency, and mechanical robustness [376,377]. Printable materials with favorable processing properties often lack the toughness needed for reliable skin penetration, and their stability under sterilization or long-term storage remains unresolved, prompting safety and regulatory concerns [378]. High-resolution printing technologies are further constrained by low throughput and high cost. The absence of suitable physiological models for evaluating efficacy and safety may delay the translation and commercialization of 3D-printed MAPs. A unified regulatory framework governing their manufacture and quality control is still lacking [377].

### 6.3 Skin regeneration

Beyond acute wound repair, the principles and technologies underpinning 3D-printed epidermal patches also facilitate skin regeneration, supporting the reconstruction of functional tissue architecture and restoration of skin integrity over longer timescales. Approaches such as allografts, cell therapies, and skin substitutes have been employed to treat hard-to-heal wounds, including large-area injuries resulting from trauma, burns, cancer, infection, or diabetes, highlighting the range of strategies available for promoting effective tissue regeneration [[379], [380], [381]]. However, challenges like insufficient donors, damage to donor site, inadequate repair, scarring, immune rejection, and high cost have hindered these strategies. Emerging skin regenerative interventions involve the use of polymer scaffolds functionalized with diverse bioactive agents, such as small drugs, peptides, growth factors, and autologous and allogeneic cells, RNA interference, and gene therapy [382]. When combined with bioactive materials, 3D bioprinting enables the creation of complex structures that mimic the natural tissues and enhance wound healing [383]. For example, Fu et al. [182] 3D printed an innovative thermosensitive pre-gel of adipose tissue dECM to fabricate tissue-engineered skin substitutes incorporating hADSCs for full-thickness wound healing. *In vivo* studies indicated accelerated wound healing with attenuation of the inflammatory response and increased angiogenesis, re-epithelialization, and collagen deposition. To develop artificial skin, Damle et al. [384] produced a 3D printed skin-specific bioink, derived from digested chicken skin incorporated into gelatin and PVA. The ink demonstrated biocompatibility, stability, and wound healing potential, inducing fast cellular recruitment at the wound site and accelerating wound healing in an animal model.

Bilayer skin substitutes have also emerged as biomimetic constructs for more effective skin regeneration. Cavallo et al. [385] fabricated a bioprinted skin substitute using a fibrinogen-based bioink to construct the dermal layer, followed by the deposition of human keratinocytes to form the epidermal layer. This approach enables precise spatial control over the layering of biomaterials and cells, effectively mimicking the hierarchical structure of native skin. The resulting tissue-like construct, comprising two distinct and functional layers, supported cell viability and proliferation, demonstrating significant potential for wound healing applications. Another bilayer cell-adaptive hydrogel with highly oriented microporous structures has been developed by Shi et al. [386] to accelerate the closure of full-thickness wounds via regulating fibroblast-to-myofibroblast transition, mitigating inflammation, stimulating angiogenesis and remodeling ECM. The biomimetic construct was built by co-culturing human keratinocytes on a 3D printed dermal layer of crosslinked gelatin methacrylate/sodium alginate hydrogel, incorporating a shear-oriented polyethylene oxide filler. Girard et al. [186] introduced a bilayer skin tissue engineering scaffold featuring an electrospun membrane at the dermal-epidermal interface, integrated with a melt electro-written open-pore scaffold that mimics the dermis. By developing a well differentiated full-thickness skin model with newly synthesized ECM within 18 days, the construct served a vibrant tool for studying wound healing and a promising graft for skin regeneration.

In a recent study, Dong et al. [387] developed multifunctional 3D bioprinted artificial skin patches with combined antimicrobial-mechanoadaptive functions to accelerate healing of infected wounds. The patches, composed of PCL, carboxymethyl chitosan, and curcumin, supported cell migration and maintained structural integrity under dynamic wound conditions. In vivo studies demonstrated enhanced wound healing, including improved wound contraction, reduced IL-6 expression, increased epithelialization, collagen deposition, angiogenesis, and macrophage polarization.

Challenges related to insufficient vascularization can be addressed through cell-mediated traction forces [388]. A 3D bioprinted, degradable, macroporous scaffold based on gelatin methacrylamide and seeded with cells was developed to promote vascularized wound healing [180]. When implanted in skin defects in nude mice, the scaffold harnessed cell-generated mechanical forces to enhance tissue regeneration by stimulating vascular connectivity, growth factor secretion, and collagen deposition. Du et al. [389] introduced a degradable astragalus polysaccharide‐containing 3D-printed scaffolds co‐cultured with VEGF165 gene‐modified iPS‐HFSCsGFP. The scaffold surface was enriched with honeycomb‐like meshwork, promoting cell proliferation. Transplantation of the tissue engineered skin onto the dorsal trauma of nude mice provided early vascularization, collagen and hair follicle regeneration, and wound repair acceleration. Adopting a novel approach, Zhang et al. [185] used skin organoids to promote in situ regeneration of large skin defects. The organoid spheres, composed of human fibroblasts, keratinocytes, and endothelial cells with a stromal core and keratinocyte surface, were 3D-printed via extrusion-based bioprinting with dual light-source cross-linking. The constructs were tailored to match wound dimensions for precise implantation. In immunodeficient mice, application to full-thickness skin defects significantly accelerated wound healing.

Other studies have shown that wound healing can be effectively accelerated by using skin-electronic interfaces with enhanced skin adhesion. In this context, Ma et al. [167] developed a 3D-printed multifunctional, diagnostic-therapeutic integrated patch using DLP micro-nano additive manufacturing technology. The patch exhibited promising mechanical and electronic properties, biocompatibility, antibacterial activity, and photothermal properties, featuring high-performance diagnostic-therapeutic sensing and effective frostbite wound healing promotion. More recently, Shin et al. [325] developed an electronic-skin (e-skin) patch involving material design, fabrication, and integration strategies for accelerating healing and monitoring of cutaneous wounds. The patch was fabricated using photolithography-compatible functional hydrogels including poly (2-hydroxyethyl acrylate as substrate, Ag flake hydrogel for interconnection, poly (3,4-ethylenedioxythiophene:polystyrene) hydrogel as working electrode, polydopamine hydrogel as tissue adhesive, and PVA hydrogel for encapsulation. *In vivo* wound healing assessments demonstrated efficient migration, proliferation, and differentiation of fibroblasts which was promoted by electric field stimulation and iontophoretic drug delivery, along with the capacity of monitoring the healing process through impedance mapping. The e-skin patch creates new opportunities for various tissue interfacing applications.

Recent advances in 4D bioprinting have enabled the fabrication of dynamic, cell-laden 3D bioconstructs using smart biomaterials, biological components, and living cells [390]. A notable development is a single-component, jammed micro-flake hydrogel with heterogeneous size distribution, introduced by Ding et al. [390] as a novel bioink for 4D bioprinting. This hydrogel combines scalable fabrication, a streamlined formulation, and rapid self-healing capabilities. It can be 3D printed into robust constructs, which may be further cross-linked to establish cross-linking density gradients through the use of a UV absorber and photoinitiator. Following shape morphing, the system enables the formation of intricate bioconstructs with precise architectures and high cell viability, underscoring its promise for cutting-edge tissue engineering applications.

### 6.4 Photodynamic therapy

**Fig. 8:**
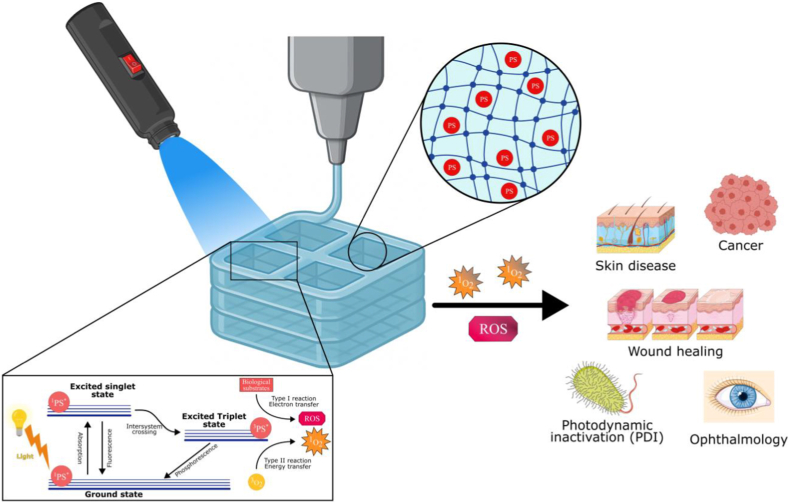
**Schematic representation of 3D printed hydrogel for photodynamic therapy.** A photosensitizer (PS) is incorporated into the hydrogel network, which remains entrapped until light is exposed. Upon irradiation, the PS undergoes a photochemical reaction that generates singlet oxygen and reactive oxygen species (ROS) through a Type I or Type II mechanism. This transparent PS-loaded hydrogel could significantly amplify the efficacy of PDT, making it highly effective for a range of therapeutic applications, including infection control, wound healing, cancer treatment, and ophthalmic interventions. This figure was partly generated using Servier Medical Art, provided by Servicer, licensed under CC BY 4.0.

Building on their roles in drug delivery, wound healing, and skin regeneration, 3D-printed epidermal patches can also support photodynamic therapy, enabling localized, controlled activation of photosensitizers. By leveraging the same conformable, biocompatible, and stimuli-responsive designs, these multifunctional patches allow precise spatiotemporal therapy while minimizing damage to surrounding tissue [391,392]. Photodynamic therapy (PDT) is a cutting-edge treatment that harnesses the power of light energy combined with photosensitizing molecules (PS) to effectively produce reactive oxygen species (ROS), such as singlet oxygen (^1^O_2_), in targeted areas [[393], [394], [395]]. When exposed to a specific wavelength or energy of light, these molecules reacts with substrates, such as intracellular components or cell membrane elements, leading to the formation of free radicals [396]. Transparent PS-loaded hydrogels present an interesting therapeutic option, rapidly advancing to enhance PDT's effectiveness across various applications, including infection control, wound healing, cancer treatment, and bone tissue formation [397,398] (Fig. 8). 3D printed patches have been developed by integrating the photosensitive Ce6-containing cyanobacterial or antibacterial agents for enhanced PDT against osteosarcoma, wound repair and bone regeneration [399,400].

Recently, Zhao and colleagues [401] developed 3D-printed artificial skin patches based on a highly effective printable bioink derived from natural biomacromolecules and a photoactive cationic conjugated poly (phenylene vinylene) derivative (PPV). The bioink not only possessed antibacterial properties but also promoted tissue regeneration, making it ideal for skin trauma. Compared to the FDA-approved anionic photosensitizer verteporfin, cationic PPV exhibited lower cytotoxicity, exceptional visible light absorption capabilities, and robust electrostatic interactions with the printing matrix. These features enhanced the patches' effectiveness in PDT, offering strong protection against *S. aureus* in both *in vitro* and *in vivo* settings.

Nanotechnology-based PDT has proven highgly effective in enhancing the delivery, stability, and targeting efficiency of PS, thereby improving therapeutic outcomes in cancer treatment [393,402] and wound healing [403]. The combination of photoactive nanomaterials with hydrogels presents another exciting opportunity for developing innovative light-based treatments, particularly to address the challenge of drug-resistant bacterial infections [404]. For instance, antimicrobial hydrogel dressings have been developed by combining traditional PDT hydrogels with NPs that possess photothermal properties [405,406] showing outstanding light-activated antibacterial activity and enhanced ROS production and accumulation. Moreover, integrating 3D printing with nanotechnology-based photodynamic therapy (PDT) offers new possibilities in modern medicine [407,408]. This approach enables the fabrication of hydrogels precisely tailored to the size and shape of the target treatment areas. The ability to produce durable, intricately designed hydrogels enhances precision, customization, and cost efficiency, positioning 3D-printed PDT systems as a promising step toward more effective and personalized healthcare solutions.

### 6.5 Flexible sensors

**Fig. 9:**
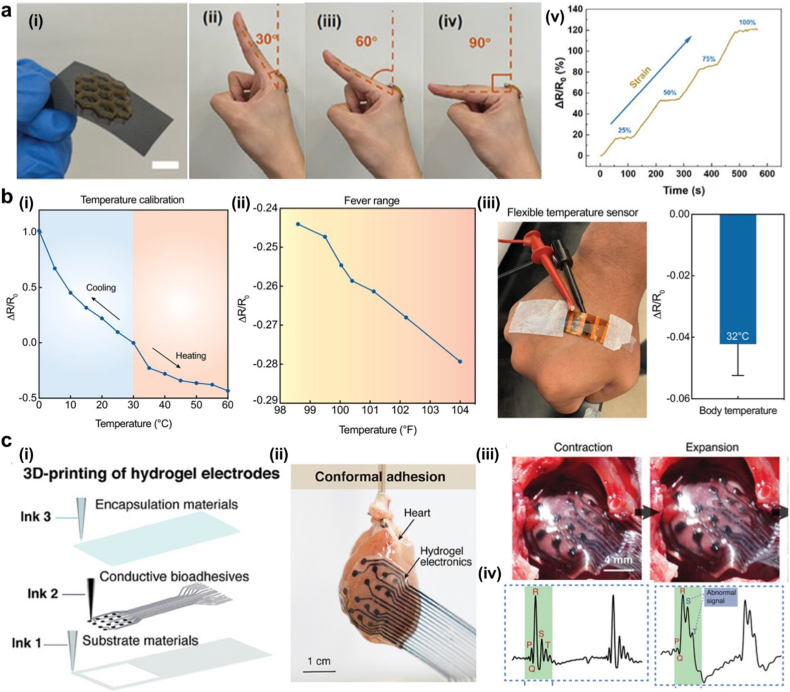
**Applications of printable hydrogels as flexible sensors**. a-(i) A honeycomb structure printed on PET film using a hydrogel based on poly (acrylamide) (PAAm), silk fibroin, poly (acrylic acid) (PAAc), and MgCl_2_ for strain sensing. (ii-iv) Real-time strain monitoring of different bending angles of finger joints. v) Strain sensing performance of the hydrogels represented by relative resistance change. Reproduced with permission from Ref. [417]. Copyright 2024, Wiley-VCH. b(i) The temperature sensing response of an electronic skin represented by relative resistance change; (ii) Fever measuring performance at a range of 98─104 °F. (iii) Real-time detection of human body temperature to monitor fever. Reproduced with permission from Ref. [419] Copyright 2023, Wiley-VCH. c(i) Multi-material extrusion printing using three inks (substrate, conductive and encapsulated materials); c(ii) Adhesiveness of the printed electrodes towards rat heart; (iii) Digital images of adhered hydrogel electrodes to the rat heart; iv) their corresponding biopotential measurements of healthy heart (left) and heart with sinus rhythm (right). Reproduced with permission from Ref. [420]. Copyright 2023, Wiley-VCH**.**

In addition to therapeutic functions, multifunctional epidermal patches can integrate sensing capabilities, enabling real-time monitoring of wound status, tissue regeneration, and treatment response. The conformable, biocompatible, and programmable architectures that support drug delivery, regeneration, and photodynamic therapy provide a platform for biosensing, bridging therapeutic intervention with continuous assessment and feedback. Literature survey indicates that 3D printed hydrogels have been utilized as flexible sensors for a plethora of applications [409,410]. These soft sensors rely on their ability to detect and respond to environmental changes, which can be mechanical, chemical and transducing, or electrical stimuli into quantitative signals. As the paradigm is shifting towards personalized medicine, hydrogel flexible sensors stand out compared to traditional sensors owing to their exceptional stretchability, flexibility, high water content and capability to mimic human tissue. One of the most extensively studied mechanisms of printed hydrogel is the change in conductivity. By incorporating conductive materials into the hydrogel matrix, electrical resistance of hydrogels changes, allowing them to sense physical or chemical signals [411,412]. Conductivity can be introduced by incorporating conductive polymers, metals, ionic agents, or carbon nanomaterials, enabling the sensing of physical parameters such as strain, motion, and pressure using conductive hydrogels [[413], [414], [415], [416]]. For instance, Wu et al. [417] prepared multifunctional printable hydrogels with chemically and physically cross-linked networks of poly (acrylamide) (PAAm), silk fibroin, and poly (acrylic acid) (PAAc). A wearable resistive-type strain sensor containing magnesium chloride (MgCl_2_) as conductive ions was fabricated by DLP-based 3D printing (Fig. 9a (i)). The sensor was then attached to the metacarpophalangeal joints of the finger, where the bending of the joints was assessed. As the bending angle of the finger increased from 0° to 60°, ΔR/R_0_ increased significantly, then decreased to 0 as the finger returned to its original position (Fig. 9a (ii-iv)). Under mechanical deformation, the distance between Mg^2+^ and Cl^−^ ions changes, resulting in a shift in the ionic conductivity of the hydrogels. Leveraging this conductivity change mechanism, the sensor exhibited a resistance change (ΔR/R_0_) that increased with the strain percentage, ranging from 25% to 100% (Fig. 9a (v)). The authors reported a gauge factor of 1.29 for tensile strain ranging from 0 to 100%. In another study [418], a mesh-structured PVA/tannic acid/polyacrylamide PVA/TA/PAM composite hydrogel was 3D-printed and functionalized with conductive carbon nanocolloids for strain sensing in large workable ranges for body movement detection. The sensor exhibited high sensitivity with a gauge factor of 32.95 (3.5%-5% strain) and 21.5 (100 %-120 % strain), good stability for up to 500 cycles, and the ability to detect subtle movements such as breathing.

In a recent study, Roy et al. [419] fabricated a 3D printed electronic skin based on pullulan (Pul) hydrogel incorporated with MoS_2_ and polydopamine (PDA) NPs for highly sensitive sensing and monitoring of human body temperature. The as-developed sensor displayed a linear temperature response over a broad range of temperature (0-60 °C) and was able to differentiate between hot and cold surfaces efficiently (Fig. 9b(i-ii)). The resistance decreased rapidly within 6 s when temperature rose to 50 °C, then steadily increased over 550 s as the temperature approached 8 °C. Real-time monitoring of the human body revealed that the sensor enabled fever detection with a sensitivity of 0.5 °F (Fig. 9b (iii)).

Printed hydrogel-based flexible sensors have also been utilized to detect physical markers, such as gait phase for minimally invasive healthcare monitoring, especially for neurodegenerative diseases, including Parkinson's Disease (PD) [421]. For example, Roy et al. [422] developed biocompatible flexible sensors based on β-cyclodextrin (β-CD), polyacrylamide (pAAm)PAM, poly 2-(acryloyloxy) ethyl trimethylammonium chloride (AETAc), and potassium persulfate (KPS) without using crosslinking agents. The printable hydrogels were used to detect abnormal gait in simulated PD patients by incorporating the sensors in gloves and monitoring normal, moderate, and severe hand tremors by measuring the current signals using the chronoamperometry method. When the movement was normal, the current signal appeared as a straight line; however, with the increase in the severity of the tremor, the frequency of the signal increased. On placing the sensors in the toe, the normal gait demonstrated sharp peaks which later vanished when the gait type was changed to abnormal in PD patients.

Printable hydrogels have also been used as electrode materials for the detection of biosignals, such as electrocardiography (ECG) and electromyography (EMG) [423,424]. For instance, Choi et al. [425] developed a self-healing PVA/agarose hydrogel metal composite. The raw data were processed using a Fourier Transform and transferred or collected in real-time via Bluetooth. For EMG measurements, the signal to noise ratio (SNR) of the developed electrode was 4.05 dB, which was significantly higher than that of commercial electrodes (2.91 dB). However, the measured ECG signals exhibited five distinct waves, closely resembling those recorded by commercial devices. In another study [420], *in vivo* monitoring of electrophysiological signals was demonstrated by adhering printable electrodes directly onto the heart. This approach successfully distinguished normal cardiac rhythms from those associated with myocardial infarction. Multimaterial electrodes were printed using three hydrogel inks, including PVA as the substrate, and Poly (3,4-ethylenedioxythiophene) polystyrene sulfonate (PEDOT: PSS) as the conductive ink and encapsulation hydrogel ink (Fig. 9c (i)). The adhesive strength of the printed hydrogels on an *ex-vivo* heart model was demonstrated even under mechanical deformation (Fig. 9c (ii)). Owing to its high conductivity, which decreases the tissue impedance, the ECG signals showed distinct atrial excitation (P wave), ventricular excitation (QRS wave), and ventricular relaxation (T wave) comparable to the commercially available stainless-steel electrodes**.** The electrodes were further employed in healthy rat hearts as well as myocardial infraction-induced hearts for *in vivo* analysis (Fig. 9c (iii)). A clear ST-segment elevation was observed in the ECG of the myocardial infraction-induced hearts, whereas this was absent in healthy hearts (Fig. 9c (iv)). These implantable electrodes provided precise electrocardiogram mapping, making them valuable for cardiovascular disease diagnosis. Other applications of printable hydrogels as flexible sensors include detecting gases [426,427], pressure [413,428] and biosensing applications [415,426,429]. Recent studies have shown that incorporating high-surface-area nanomaterials-such as MWCNTs, GO, and AuNPs-within SLA, DLP, or extrusion-based printed hydrogel matrices markedly enhances transduction efficiency, structural integrity, and analyte sensitivity, facilitating wearable biosensors with improved signal stability [430].

In addition to conductivity measurements, other sensing mechanisms, such as piezoresistive, capacitive, triboelectric, and piezoelectric properties, are gaining increasingly popularity [431,432]. Piezoresistive sensing is the most widely used mechanism of printable hydrogels, owing to the ease of fabrication and high sensitivity. These sensors rely on electrical resistance change when mechanical strain or pressure is applied, making them suitable for sensing pressure, force, and motion. Several studies have reported that the sensitivity of piezoresistive sensors can be improved by introducing micropores into the structure, advocating for the importance of 3D printing technology in designing hydrogels for sensing [[433], [434], [435]].

Capacitive sensors typically consist of two electrodes, by which they detect changes in capacitance when the distance between the two electrodes changes. When an external stimulus, such as pressure, is applied to the printable hydrogel surface, an increase in the capacitance occurs due to the decrease in the distance between the two electrodes [436]. It has been reported that the performance of the capacitive sensors depends on the electrical conductivity of the electrodes and the deforming ability of the dielectric layers, thus making printable hydrogels promising choices for both the dielectric and the electrode layer [437,438].

In recent years, various research groups have used piezoelectric sensors for dynamic pressure monitoring [439,440]. Unlike piezoelectric sensors which require specific materials, triboelectric hydrogel sensors offer a wide variety of material choices, making them suitable for diverse sensing applications [441]. As the hydrogel interacts with another material or with materials within the same matrix, the frictional contact and separation create an electric charge that can be detected as a signal.

In addition to these established flexible sensing mechanisms, recent studies have demonstrated significant advances in hydrogel and microneedle-based sensors integrated into closed-loop platforms for real-time monitoring and on-demand therapy. For example, Liang S. et al. [442] have recently developed a self-powered, closed-loop epidermal patch that integrates real-time skin hydration detection, drug microneedle therapy, and energy harvesting for the treatment of atopic dermatitis. Their patch incorporates a piezoelectric generator that captures mechanical energy from patient motion to power the system, along with a hydration sensor, a microneedle treatment module and flexible circuit. Upon detecting abnormal hydration levels for 65 s, the system automatically activates the therapeutic module by heating hyaluronic acid-based microneedles (∼42 °C) to release dexamethasone sodium phosphate (DEX), thereby providing timely targeted therapy and moisturization to the affected area within minutes. Therapeutic results in a mouse model of atopic dermatitis demonstrate that this device can successfully treat atopic dermatitis, improving epidermal thickness, IL-4 levels, and spleen size and mass. Parrilla M. et al. [443] have introduced the first microneedle based electrochemical sensor for transdermal monitoring of methotrexate (MTX). They modified hollow microneedles with conductive pastes to develop a three-electrode electrochemical system, and the working electrode was functionalized with crosslinked chitosan (CHI/GA) to provide antibiofouling and preconcentration capabilities. The functionalization with CHI/GA enables selective adsorption of MTX at the electrode surface while preventing protein diffusion. The functionalized sensor was characterized both *in vitro*, using protein-enriched artificial interstitial fluid (AISF), and *ex vivo*, demonstrating high linearity, reversibility, and long-term stability essential for wearable applications. Complementarily, an iontophoretic hollow array system (IHMAS) was developed for on-demand transdermal MTX delivery, providing a versatile platform for closed-loop therapeutic management.

Leveraging these strategies for continuous biomarker monitoring, Dai Y. et al. [444] developed wearable sensor patches incorporating hydrogel microneedles for minimally invasive extraction of interstitial fluid (ISF) and on-site electrochemical analysis. The hydrogel microneedles, composed of methacrylated hyaluronic acid (MeHA), were engineered with optimized size and composition to maximize ISF extraction and minimize tissue invasiveness. The patch integrates a three-electrode electrochemical system, with glucose oxidase (GOx) and lactate oxidase (LOx) immobilized on Prussian Blue-modified electrodes, enabling simultaneous detection of glucose and lactate. *In vitro* evaluations showed sensitivities of 0.024 ± 0.002 μA mM^−1^ for glucose and 0.0030 ± 0.0004 μA mM^−1^ for lactate, with linear ranges of 0.1–3 mM and 0.1–12 mM, respectively. *In vivo* studies in a mouse model confirmed continuous glucose monitoring with a sensitivity of 0.020 ± 0.001 μA mM^−1^ and a detection range of 1–8 mM, demonstrating excellent correlation with commercial meters. Lactate sensing revealed characteristic peak-response patterns consistent with metabolic pathways. This platform integrates predictive modeling to compensate for signal delays, achieving response times of less than 10 min for glucose and lactate, outperforming conventional ISF or blood-based measurements. The combination of hydrogel microneedles, stretchable electronics, and advanced electrochemical circuits provides a robust, reliable, and minimally invasive platform for continuous, real-time monitoring of multiple ISF biomarkers.

In recent years, printable hydrogel-based sensors have shown significant promise as flexible and biocompatible devices for real-time healthcare monitoring, personalized medicine, wearable devices, and implantable diagnostics [445]. Hydrogels serve as wearable, skin-conforming devices that enable non-invasive and continuous health data collection, making them particularly valuable for the management of chronic conditions such as diabetes [446] or cardiovascular diseases [447]. Additionally, hydrogel-based sensors could be incorporated into implantable systems for drug delivery and *in vivo* monitoring, for example, real-time glucose [435] or pH detection [448]. Hydrogel sensors have been demonstrated as effective substrates in diagnostic platforms for detecting biomarkers in blood, saliva, or tears, aiding in rapid disease detection and personalized therapeutic interventions [435].

A promising approach to advancing sensor technology lies in the development of multifunctional capabilities. Beyond the conventional adherent sensors, where strain arises directly from the substrate to which the sensor is affixed, strain can also be remotely induced by external stimuli such as pH variations, electric fields, or magnetic fields. The integration of additive manufacturing with stimuli-responsive smart materials, capable of altering their shape, functionality, or performance in response to environmental triggers, underpins the rapidly evolving field of 4D printing. For example, Shi et al. [449] utilized Pluronic F-127 diacrylate to fabricate biocompatible micelle-based hydrogels with high structural precision via DLP 3D printing. These hydrogels exhibited shape-memory behavior upon rehydration, enabling conformal attachment and demonstrating 4D printing characteristics. LiCl treatment produced conductive ionic hydrogels with antifreezing, antiswelling, and water-retentive properties. As capacitive flexible sensors, they show high sensitivity, durability, and stable performance in multimodal sensing, even at −20 °C. Chen et al. [450] introduced a hybrid 4D printing strategy that integrates multi jet fusion (MJF) with direct ink writing (DIW) to fabricate multifunctional liquid crystal elastomer–shape memory polymer (LCE-SMP) composites. This approach enables rapid production of SMPs with tunable electrical conductivity, followed by DIW printing of LCEs with programmable mesogen alignment. The resulting composites exhibit reversible photo-actuation with high output power and robust self-sensing capabilities, offering real-time feedback on performance during actuation tasks.

Using a different approach, Hou et al. [451] developed a 4D-printed ultraflexible strain sensor that balances high sensitivity and broad sensing range while incorporating multifunctionality. The sensor is composed of carbon nanotubes/liquid metal hybrids and iron powders embedded in an Ecoflex matrix, optimized for DIW. The cured sensor demonstrated excellent electromechanical, thermal, and magnetic properties, including thermal stability above 300 °C, improved conductivity, and a stable resistance-temperature profile. Magnetic responsiveness enabled additional functions like location and speed detection, making it suitable for high-temperature and home care applications. Another 4D-printed sensor based on pyramid Kirigami mechanical metamaterials (PKMM), integrating triboelectric and piezoelectric mechanisms for multi-dimensional mechanical signal detection sensor has been developed by Liu et al. [452]. The sensor features shape reconfigurability, surface conformality, and high sensitivity across a broad pressure range. It enables precise displacement monitoring, conformal tracking of elbow motion, and multi-point load analysis through an axial and modular island-bridge array. The design demonstrates strong potential for applications in biomechanical monitoring and smart protective gear. Moreover, bioinspired chiral metamaterials with wave ligaments, modeled after the collagen fiber architecture of biological tissues, were 4D-printed for use in flexible sensors [453]. The bi-phase TPE@PLA-SMP composites combine shape memory polylactic acid (PLA-SMP) as the active phase with thermoplastic elastomer (TPE) as the passive phase. Their mechanical performance was modulated by tuning geometric parameters, ligament gradients, and TPE distribution patterns, with 0° and 45° TPE orientations producing characteristic J-shaped displacement–force curves. The metamaterials exhibited programmable, thermally reconfigurable structures and tunable energy absorption, with specific energy absorption ranging from 0.92 to 0.38 kJ/kg. Flexible sensors fabricated by integrating cellulose nanocrystals (CNCs) and carbon nanotubes (CNTs) into a PVA hydrogel matrix (CNC-CNT@PVA) delivered stable electrical signals under repeated mechanical loading. A wearable motion monitoring system further demonstrated the platform's potential for applications in flexible electronics and smart wearables.

However, most of these applications remain in the preclinical or developmental phase. As such, their progression to clinical trials requires thorough safety and efficiency evaluations, along with compliance with regulatory requirements. Key challenges, including biocompatibility, long-term stability, integration with electronic systems, and large-scale production, must be addressed to ensure successful clinical translation.

## 7 3D printed epidermal patches for personal care innovation

Extending beyond therapeutic and diagnostic applications, 3D printed epidermal patch designs also support cosmetic and broader personal care applications, enabling localized delivery of cosmetic bioactive agents, skin hydration, and personalized skincare while leveraging the same conformable, responsive, and programmable architectures. Epidermal patches and MAPs have gained increasing popularity for routine self-care, particularly for targeted skin areas, such as wrinkling skin of the face, neck, or eyelids, as well as for treating scars, zits and blemishes [454]. Additionally, full-face sheet masks designed to treat skin conditions like acne or improving skin condition [455,456], are experiencing growing consumer satisfaction and widespread use. However, conventional skin patches and MAPs are lagging behind the growing demand for product personalization and consumer preferences for specific cosmetic ingredients and product formulations [457]. Demand for personalized skin care is expected to further increase with the advent of wearable sensors/devices, smart device applications, and AI, allowing identification of the consumer's skin characteristics [[458], [459], [460], [461]]. In this context, the application of 3D-printing in the cosmetic and personal industry holds great promise, potentially ushering in an era of personalized products that offer greater efficacy and consumer satisfaction.

### 7.1 Conventional patches

Despite great prospects, research efforts involving 3D-printing of conventional patches for cosmetic applications are scarce, as indicated by the limited number of detailed review articles [23,462]. A research topic that attracted early attention in this respect implicated anti-acne 3D-printed patches [[463], [464], [465]]. For instance, adhesive patches and masks loaded with salicylic acid, as comedolytic agent, were personalized to the shape of a patient's nose using FDM and SLA 3D-printing techniques for acne treatment [463]. The SLA technique provided a nose-shaped patch with better resolution, higher drug loading, and faster release with maintained drug stability. Another personalized anti-acne patch with specific shape and size was produced by extrusion-based 3D printing of a hydrogel incorporating niosomes, as a delivery system, encapsulating the anti-acne drug cryptotanshinone [465]. Data from a rat model demonstrated that integrating drug delivery with 3D printing technology significantly enhanced the patch's efficacy and safety. Hashem et al. [464] developed topical salicylic acid anti-acne patches using Eudragit EOP for FDM-based 3D printing. This approach allowed printing at a lower temperature which preserved the drug stability.

3D-printed hydrogel patches infused with salicylic acid and metronidazole were utilized to treat rosacea and maskne conditions induced by prolonged use of personal protective face masks among healthcare professionals and the general public [466]. Other cosmetic skin conditions were also treated by personalized skin care hydrogel patches fabricated by extrusion-based 3D printing. For example, Manousi et al. [24] developed extrusion-based 3D printed hydrogel face patches using Iota carrageenan as base material of the hydrogel inks. These were co-formulated with sodium hyaluronate and glycerol as moisturizers along with *Camellia sinensis* leaf distillate as antiseborrheic agent to address the requirements of individual skin types. In this context, Chen et al. [467] designed a partition multi-effect 3D-printed precision-care gel facial mask to accommodate the physiological characteristics of different parts of the face. The mask enabled the delivery of hexapeptide to the wrinkled forehead and nasolabial fold, arbutin to the pigmented cheek for a whitening effect, and salicylic acid to the nose and chin for oil control. The mask could overcome the inadequacies of the single skincare effect of commercial face masks. Bom et al. [468] investigated personalized under-eye 3D-printed skincare patches tailored to individual skin needs and preferences, and assessed consumer acceptance using emotional sensing compared to a conventional market product. Affective analysis indicated greater consumer acceptance of the 3D-printed patches, highlighting the potential of advanced technologies to enhance skin health and user satisfaction.

Recent advances included the development of 3D-printed transparent face masks for preventing and treating facial hypertrophic scars in burn patients, including young children [[469], [470], [471]]. A custom facial mask was developed using a smartphone 3D scanner and desktop 3D printing of rigid polylactic acid (PLA) filament and semi-rigid thermoplastic polyurethane [472]. The digital workflow for the production of the 3D-printed mask is patient-friendly and can be used for resource-intensive healthcare. To address inadequate pressure distribution of burn scar by conventional facial compression masks in curved areas, e.g., cheeks, slope of the nose and around the mouth, Hwang et al. [473] developed a customized 3D-printed compression mask with pressure sensors. A 12-week clinical controlled trial involving 48 facial burn scars in 12 inpatients indicated significant improvement in scar thickness, skin hydration, and various assessment scale parameters.

In addition to 3D printing, multidimensional printing technologies are increasingly being investigated in the realm of personalized care, showing significant potential for cosmetotextiles. In this context, 4D printing shows significant potential for the development of cosmetotextiles, intelligent fabrics incorporating shape-memory polymers (SMPs) that can respond to individual skin needs by modulating the release of active ingredients such as moisturizers, anti-aging compounds, or antimicrobial agents [34]. These materials can also dynamically adjust garment fit based on real-time physiological data, offering highly customized and responsive skincare solutions.

### 7.2 Microneedle array patches (MAPs)

3D printing endows MAPs for cosmetic and dermatological applications with unique advantages, enabling the fabrication of complex microstructures and tip profiles using minimal resources to achieve personalized effects such as anti-aging, skin hydration, and rejuvenation [474,475]. Although the application of 3D-printed MAPs in the cosmetic field is still in its infancy, their potential has been documented by a few recent studies. For example, a DLP 3D-printed personalized MAP fabricated using a PEGDA/NVP photopolymer was used for the delivery of acetyl-hexapeptide 3 (AHP-3), a hydrophilic and large molecular weight anti-wrinkle small peptide [476]. The ability to penetrate human cadaver dermatomed skin, while maintaining the MNs integrity after compression and minimal cytotoxicity of the polymer to human dermal fibroblasts, demonstrated its potential for effective wrinkle management. In another study, Islam et al. [194] explored the use of 3D-printed hollow MNs for the treatment of skin wrinkles using diverse anti-wrinkle agents, comparing their effectiveness to conventional treatments like lasering and Botulinum toxin.

For potential hypertrophic scar treatment, morphology-customized microneedles—featuring spiral, conical, cylindroid, ring-like, arrow-like, and tree-like shapes—were fabricated using SLA 3D printing. Incorporation of Rhodamine B enabled visualization of shape-dependent skin penetration [372]. In vivo studies using New Zealand rabbit models demonstrated that the tree-shaped 3D-printed MNs loaded with verapamil significantly enhanced transdermal drug delivery and therapeutic efficacy for hypertrophic scar treatment. More recently, a triamcinolone acetonide-loaded MAP was 3D-printed by FDM using PLA/chemically treated river snail shell powder composites for keloid treatment [477]. The patch efficiently enhanced the drug percutaneous delivery, demonstrating a promising clinical potential.

Beyond skin applications, a customized 3D-printed MAP was developed by Yuan et al. [478] for hair regeneration using static optical projection lithography. In a mouse model, the patch promoted enhanced hair regrowth with improved quality within a precisely controlled area. Cellular and molecular analyses revealed in situ recruitment of macrophages, initiation of hair follicle stem cell proliferation, and activation of the Wnt/β-catenin signaling pathway. These findings underscore the potential of 3D-printed MAPs as a personalized therapeutic strategy for hair loss, an often-distressing dermatological condition with substantial psychosocial impacts.

However, the fabrication of 3D-printed MAPs for cosmetic applications faces several challenges, including the selection of suitable materials, limited manufacturing throughput, and an incomplete understanding of how complex geometries influence functional performance [372]. Furthermore, the lack of appropriate physiological models for evaluating efficacy and safety poses an additional barrier, potentially delaying the translation and commercialization of cosmetic 3D-printed MAPs.

Overall, 3D-printed epidermal patches constitute a multifunctional skin-interfacing platform that spans dermal and transdermal drug delivery, wound healing, and skin regeneration through shared material and design principles. Advanced architectures further enable photodynamic therapy, sensing, and cosmetic applications, supporting spatiotemporally controlled, adaptive, and personalized skin interventions.

## 8 Clinical trials

A comprehensive analysis of clinical trials of printable hydrogel patches was performed using the ClinicalTrials.gov database. All the active, completed, recruiting, and enrolling trials were included, while terminated, suspended, or withdrawn trials were excluded. An initial collection of potentially admissible clinical trials was gathered by searching for “3D printing AND hydrogel” (3 trials) and “3D printing AND surgical patch” (4 trials) in trial intervention and “3D printing AND hydrogel” (3 trials) and “3D printing AND surgical patch” (16 trials) as a search terms. The dataset was refined by removing duplicates, excluding observational or non-interventional clinical trials, and excluding trials where 3D printing was not actively involved. The resulting dataset contained 14 unique clinical trials, as summarized in Table 4. It is noteworthy that some of the trials have used 3D printing of hydrogels for educational purposes to recreate models of complex pathologies, enabling better surgical approaches. This approach is particularly well-suited for pediatrics, which suffers from the rarity of its pathologies and a large spectrum of size and morphology [479].

**Table 4:** Representative clinical trials on 3D printed hydrogels and patches.

National Clinical Trial (NCT) Number	Study Title	Acronym	Conditions	Sponsor	Collaborators	Reference
NCT03416387	Applicability of 3D Printing and 3D Digital Image Reconstruction in the Planning of Complex Liver Surgery	LIV3DPRINT	Liver diseases, surgery	Hospital Universitario Virgen de la Arrixaca	-	[480]
NCT06147024	Utilizing 3D Printed Personalized Aortic Lesion Models in Preoperative Assessment	-	Aortic aneurysm	Fu Jen Catholic University	-	[481]
NCT05700526	Customized Bone Allografts by 3D-printing	3D-MALF 2	Musculoskeletal deformity, Musculoskeletal diseases, Musculoskeletal disorder	Istituto Ortopedico Rizzoli	University of Bologna	[482]
NCT04505020	The Innovation of 3D Printing for Preoperative Planning in Hip Preservation Surgery	-	Femoro acetabular impingement	Nova Scotia Health Authority	-	[483]
NCT06291662	Evaluation of Performance Characteristics and Applicability in Oncology of Devices Customized Medical Devices Made by 3D Printing	-	Sarcoma, intracranial neoplasm, pelvic bone neoplasm	Kathleen McGreevy	-	[484]
NCT04552054	Mixed Reality Technique Combined With 3D Printing Navigational Template for Localizing Pulmonary Nodules	MR&3D Local	Video-assisted thoracic surgery, lung cancer, pulmonary nodule - solitary, pulmonary nodule - multiple	Wen-zhao Zhong	Guangdong Provincial People's Hospital	[485]
NCT03964064	I125 Seed Implantation vs Stereotactic Radiotherapy for Pancreatic Cancer	Ckvssip	Pancreatic cancer non-resectable, brachytherapy, radiotherapy	Peking University Third Hospital	Beijing 302 Hospital, Guangxi Ruikang Hospital, Tengzhou Central People's Hospital	[486]
NCT05982418	Improvement of RARP Outcomes Via 3D Printed/Virtual Prostate Models	RARP-3D	Prostate cancer	Guy's and St Thomas' NHS Foundation Trust	King's College London	[487]
NCT03913416	Can Pre-operative Flexible 3D Models of Pulmonary Malformations Facilitate Thoracoscopic Resection	3DLP	Pulmonary malformation	Hospices Civils de Lyon	-	[488]
NCT05283252	The 3-Dimensional Printed Guide in Endodontic Microsurgery	-	Endodontic disease, endodontic re-treatment failure	Damascus University	-	[489]
NCT04266327	RISI in the Treatment of Recurrent Metastatic SCC of Thoracic Inlet Lymph Nodes	-	Brachytherapy, squamous cell carcinomas	Peking University Third Hospital	-	[490]
NCT06051747	Patient-Customized Bioprinting Technology for Practical Regeneration of the Respiratory Tract	Trachea	Thyroid cancer	Ja Seong Bae, MD, PhD	Korea Health Industry Development Institute	[491]
NCT04399239	AuriNovo for Auricular Reconstruction	AuriNovo	Microtia	3DBio Therapeutics	-	[492]
NCT06782711	Efficacy and Safety of 3D-Printed PEEK Skull Implants in Cranioplasty	-	Cranial defects	Cimet scientific corporation	Consejo Nacional de Humanidades, Ciencias y Tecnologias	[493]

Recent clinical trials have explored the use of 3D-printed hydrogels in surgical simulation to enhance preoperative planning. Notably, 3D templates have shown promise in modeling bronchial (NCT04552054) [485], vascular (NCT06853054) [494], and parenchymal anatomy (NCT03913416) [488]. This permits a better understanding of the anatomical particularities of each patient, reducing the risk of intra-operative conversions to thoracotomy with a direct benefit for the patients. The 3D printing technology has also been employed to fabricate personalized aortic blood vessel models with lesions (NCT06147024) [481], 3D models for percutaneous nephrolithotomy (NCT03272529) [495], 3D prostate models (NCT05982418) [487], and simulation of bone corrections (NCT05700526) [482]. These studies have determined that the surgeons’ anatomical knowledge is enhanced by the manipulation of 3D printed/virtual models constructed from automated segmentations, thus reducing surgical errors while providing better positive resection margins and functional outcomes. It is pertinent to point out that findings of these investigations have not been yet fully disclosed. Anyway, progress in clinical applications for 3D-printed hydrogel scaffolds is still limited, as there are very few clinical trials underway to assess their potential benefits. For instance, a phase 1/2 trial conducted between 2021 and 2023 aimed to gather preliminary safety data on unilateral microtia ear reconstruction using AuriNovo™ (NCT04399239), a 3D-bioprinted living tissue ear implant based on collagen [492]. Another phase 1/2 trial started in 2023 and expected to end in 2025, involving the creation of a patient-specific 3D-printed trachea loaded with nasal cavity stem cells and nasal septum cartilage cells (NCT06051747) [491].

Despite significant advancements in the field of 3D bioprinting, the limited number of clinical trials reveals that the transition from laboratory research to clinical practice remains slow and requires further investigation and effort. Challenges and technological hurdles, such as technical complexity, safety concerns, and funding limitations, hinder the initiation of clinical trials for these innovative technologies [496]. For instance, ensuring sterility in highly porous or hydrogel-based printed structures remains technically demanding, as conventional sterilization methods such as autoclave and gamma irradiation could irreversibly alter material properties or compromise structural integrity. Similarly, maintaining the viability, functionality, and reproducibility of cell-laden constructs during fabrication, storage, and implantation is technically challenging and represents a major hurdle for clinical translation. These issues complicate compliance with GMP requirements and increase the complexity of scaling up production for clinical use. In addition, translating the results from *in vitro* and animal studies to humans is difficult because of biological variability. Before initiating a clinical trial, it is also crucial to assess the safety of the technology to prevent any adverse effects on patients. This process demands substantial time and resources and the lack of harmonized regulatory guidelines, together with ethical considerations related to the fabrication of functional tissues and complex biological constructs, continues to slow clinical research and commercialization pathways. Nevertheless, 3D printing technologies are expected to experience substantial growth in the coming years. As technical challenges related to material standardization, manufacturing reproducibility, and regulatory approval pathways are progressively addressed, an increasing number of preclinical studies and clinical trials will likely emerge, and help to accelerate the translation of these technologies into clinical practice.

## 9 Regulatory challenges and market potential

The rise of the 3D printing industry presents a significant challenge for regulators, as the existing regulatory frameworks are designed for mass-manufactured, standardized products, and are not well-suited to ‘mass-customized’ patient-specific devices [[497], [498], [499]]. The regulatory challenge of 3D-printed medical devices does not only stem in the complex nature of 3D printing technology but also in the unique characteristics of personalization and decentralization of this manufacturing process [500]. Traditional manufacturing methods have created standard production processes that are largely unaffected by the manufacturers or locations involved. However, 3D printing has introduced variability, challenging regularity stability and adding complexity to the oversight of decentralized manufacturing. Additionally, many 3D-printed devices have been implanted under the “custom-made" exemption, often bypassing rigorous regulatory oversight. In many jurisdictions, the regulatory framework excludes patient-specific devices based on the assumption that these devices pose a lower risk or are intended for exceptional cases rather than for standard treatment [499,501]. From a regulatory standpoint, it is essential to define how these products are classified, whether as pharmaceuticals, biologics, medical devices, or a combination of these categories. Accurate classification of a new product is crucial for commercialization, as it determines the regulatory strategy, timelines, the investment needed for evidence and documentation, and potential risks to the business [502]. The rapid evolution of these technologies makes it challenging to define a standardized translational pathway. Typically, translation proceeds from prototype fabrication and *in vitro* or animal testing, through regulatory consultation and GMP-compliant manufacturing, to phased clinical trials and regulatory submission, followed by post-market surveillance to ensure long-term safety and performance. Safety and quality considerations are central to clinical translation, encompassing material biocompatibility, mechanical integrity, dose delivery consistency, sterility, and post-application skin compatibility, in accordance with standards such as ISO 13485, ISO 14971, and ISO 20417.

3D-printed devices are rapidly evolving, transitioning from passive constructs such as titanium-based hip implants designed for minimal interaction with surrounding tissues [503] to bioactive materials like tissue regeneration scaffolds that actively stimulate and support new tissue growth. This active role directly drives a crucial change in the applicable regulatory framework, aligning it with that of medicinal products [504]. If a 3D-printed solution is classified as a medicinal product, it will require extensive documentation and stricter market entry standards, resulting in lengthier and costly clinical trials. While this process guarantees high safety and efficacy standards, it may take longer to complete. While research and development costs will increase, the end products will be offered to healthcare providers at a price that reflects their quality and effectiveness.

New regulatory challenges emerge whenever a new manufacturing process is developed. The challenges can stem from technical aspects of the automated fabrication process, such as variability in the fabrication methods, digital data processing, and the software system used. Additionally, the undefined properties of bioprinted products can pose challenges, such as inconsistencies in the strength and stability of scaffolds. 3D-printed devices, especially hydrogels, are typically fabricated by combining various bioprinting materials to facilitate printing (commonly through extrusion) or to produce new composite biomaterials with specific functions. The effectiveness and safety of the developed biomaterials remain primarily uncertain, and the available information is frequently minimal. It is crucial to address this gap to ensure confident and informed use in clinical applications. On the other hand, components used in bioinks, such as cell culture media, biological agents, and natural polymers, may raise significant safety concerns, as they could contain regulated or potentially bioactive substances requiring thorough evaluation [499]. Unlike the traditional medical device industry, where material properties are well-defined and standardized with established risk profiles, a lack of certified “medical grade" bioprinting materials for human clinical use exists. Beside the characteristics of biomaterials, the fabrication process may lead to structural defects, including irregular surfaces, edge flaws, or interlayer inconsistencies [505]. Therefore, it is essential to address and mitigate these risks to ensure the safety and effectiveness of bioprinted products.

Incorporating living cells into 3D printing inks or resins enables the fabrication of living 3D constructs for applications in regenerative medicine and disease modeling, while also introducing an added layer of complexity to the printing process [499]. From a translational and regulatory perspective, 3D bioprinted products face all the challenges associated with conventional 3D printed devices and those specific to regenerative medicine, tissue engineering, biomaterials, and stem cell technology [506]. Therapies that include live cells introduce significant complexity and uncertainty risks, all of which must be considered when developing such constructs [507,508]. This increased complexity suggests that new regulations for 3D printed products may not automatically apply to 3D bioprinted products.

Regulators face the challenge of balancing the need to mitigate potential risks associated with new medical devices, while allowing commercial development and freedom to innovate within the sector. Overly stringent regulations may hinder product development that benefits consumers, but it is essential to consistently and transparently protect public health [502]. This challenge is especially evident in emerging technology fields like 3D printing in medicine, where the potential risks are often unclear. The rapidly evolving printing technologies necessitates the creation of a flexible regulatory framework that goes beyond the existing static traditional regulations. The current regulatory frameworks established by bodies, such as the U.S. Food and Drug Administration (FDA) and the European Medicines Agency (EMA), are primarily designed for traditional manufacturing methods. Many patient-specific 3D printed devices have been implanted without significant regulatory oversight because they fall under the ‘custom-made’ exemption. However, these regulations were created when personalized devices were uncommon, and it is becoming increasingly clear that the ‘custom-made’ exemption may no longer be acceptable from a risk management perspective [499,506,509,510]. In 2021, the Therapeutic Goods Administration (TGA) in Australia made notable efforts to refine and clarify the frameworks governing medical devices. They subdivided and expanded the definition of “personalized medical devices” into new subcategories, which include “custom-made,” “patient-matched,” and “adaptable medical devices” [511]. Despite these advancements, challenges remain in aligning current regulatory approaches with the rapidly evolving field of 3D printing technology.

Addressing regulatory challenges is essential for ensuring patient safety and the effectiveness of medical products, as well as for understanding the economic implications tied to the development and commercialization of 3D printed medical devices. Current market estimates indicate a rapid increase in the use of 3D-printed medical devices, with the market projected to grow from $2123.11 million in 2021 to $6583.50 million by 2028, at a compound annual growth rate (CAGR) of 17.5% [512]. A well-defined regulatory landscape featuring standardized testing and a streamlined approval process could foster opportunities for growth and innovation in the 3D printing industry, particularly for smaller companies and startups. A robust regulatory framework can also enhance market credibility and increase consumer trust.

The rapid advancement in research and development could give rise to a new wave of regenerative therapies that could outpace the current regulatory framework, which is primarily focused on specific product types. For instance, the introduction of AI-driven personalized design has the potential to significantly reduce the economic costs associated with 3D-printed medical devices [513]. However, the integration of AI technologies in medicine presents both technical and regulatory challenges [499]. In today's rapidly evolving landscape of 3D-printed medical devices, it is crucial to implement a tailored regulatory system. By fostering strong collaborations between regulatory agencies and industry leaders, it is possible to address these challenges effectively and establish a cohesive, predictable regulatory environment that promotes innovation while ensuring patient safety.

## 10 Conclusions and future perspectives

The landscape of epidermal patches has been fundamentally reshaped by the integration of 3D printing technologies, which have enabled unprecedented control over material composition, structural design, and functional performance. This review has illustrated how both conventional and microneedle-based patch platforms are benefiting from innovations in printable hydrogel formulations and multi-dimensional printing methods. Protein-based, polysaccharide-based, and nanocomposite hydrogels, each offering tailored mechanical, rheological, and biological properties, facilitate precise modulation of drug release kinetics, skin conformability, and cellular compatibility. Emerging hydrogel systems with enhanced functionalities, such as nanomaterial-free, self-oxygenating, and stimuli-responsive variants, represent a substantial evolution toward “smart” epidermal interface. These materials are capable of interacting with their physiological environments, responding to pH, temperature, or enzymes to optimize therapeutic performance. Such properties, combined with 4D and emerging 5D printing techniques, are driving the development of next-generation patches that not only mimic native skin microarchitecture but also adaptively respond to movement, biochemical cues, and environmental stimuli.

Hydrogel-based biosensors, integrated into these platforms, offer promising tools for non-invasive, continuous health monitoring, particularly for chronic diseases such as diabetes and cardiovascular disorders, by detecting biomarkers like glucose, pH, or electrolytes in real-time via sweat or interstitial fluid. One of the key takeaways from the current body of research is the growing convergence between engineering precision and biological functionality. The refinement of printing resolution and fidelity, augmented by 4D and 5D printing techniques, now enables the fabrication of patches that not only mimic skin microarchitecture but also dynamically respond to real-time physiological or environmental stimuli. These materials support personalized, responsive therapeutic strategies and set the stage for advanced systems capable of simultaneous biosensing, drug delivery, and tissue regeneration.

In skin care, 3D printed epidermal patches are rapidly evolving beyond traditional delivery systems. Personalized hydrogel-based facial masks and microneedle arrays have been developed to address conditions such as acne, rosacea, hyperpigmentation, or skin aging. These patches are tailored not only in shape but also in content using combinations of peptides as well as moisturizing agents or anti-inflammatory agents based on skin parameters. Among the most promising advances in cosmetic dermatology are microneedle patches fabricated via high-resolution 3D printing techniques such as SLA, DLP or FDM. These patches enable precise control over microstructure geometry, improving skin penetration, mechanical stability, and drug delivery efficiency.

Despite significant advances, clinical and commercial translation remains limited by persistent challenges in scalability, regulatory compliance, and long-term biocompatibility. For instance, while many materials and devices perform well in preclinical studies, their transition to human use is hampered by complex regulatory frameworks and inconsistent standards for 3D printed biomedical devices. Moreover, the integration of living cells or bioactive molecules into patches adds layers of complexity in terms of sterilization, storage, and distribution logistics. These aspects necessitate concerted efforts among regulatory bodies, researchers, and manufacturers to develop standardized protocols for quality assurance and regulatory approval. Furthermore, the biological interface of epidermal patches demands deeper investigation. Long-term studies on skin integrity, immune responses, and microenvironmental interactions are essential to ensure safety and efficacy. There is also a need to better understand how different skin types, across age, ethnicity, and pathology, interact with various patch designs and materials. This includes evaluating the impacts of mechanical forces, humidity, and microbiota on the performance and degradation of patches over time. Addressing these variables will be crucial in optimizing patch performance for diverse patient populations.

From a fabrication standpoint, although 3D and 4D printing technologies are steadily maturing, 5D printing still requires substantial development in hardware, software, and cost-efficiency to be viable at scale. The ability to manipulate materials along multiple axes holds immense potential for producing anatomically conformal patches that adhere seamlessly to complex skin topographies; however, current technological and cost limitations restrict widespread adoption. Future research must focus on improving printing speed, resolution, and multi-material integration to make these advanced techniques more accessible and clinically viable. On the other hand, ongoing and future clinical trials play a crucial role in validating the real-world applicability of 3D-printed epidermal patches from both clinical and translational standpoints. Current investigations into wound healing and transdermal drug delivery are promising, but broader clinical evidence is necessary to validate efficacy, safety, and cost-effectiveness across different therapeutic areas. As such, collaborative networks that integrate academic research, clinical expertise, and industrial know-how will be instrumental in accelerating the path from bench to bedside.

Looking ahead, the integration of biosensing capabilities with epidermal patches represents one of the most exciting frontiers. The coupling of microelectronics with 3D printed hydrogel structures could allow for real-time monitoring of biomarkers such as glucose, pH, or cytokines, creating multifunctional patches that diagnose, deliver, and adapt therapeutics simultaneously. Such systems would be especially valuable for chronic conditions like diabetes, eczema, and psoriasis, where continuous monitoring and adaptive treatment could dramatically improve patient outcomes. In the cosmetic sector, the fusion of AI-driven skin diagnostics with customizable 3D printing is likely to revolutionize how consumers interact with skincare products. Home-use printers or point-of-care devices could fabricate patches tailored to an individual's current skin condition, informed by real-time imaging or sensor data. This hyper-personalized model not only enhances consumer satisfaction but also promotes the development of data-driven cosmetic formulations with quantifiable benefits.

Future progress in this field is expected to follow a gradual and structured roadmap. As discussed throughout this review, substantial efforts have been devoted to optimizing printable biomaterials, improving printing resolution, and validating patch performance in preclinical models. In the short term, the integration of multifunctional capabilities, including biosensing modules, stimuli-responsive systems as platforms for drug delivery, and enhanced skin conformability for wearable applications, is expected to further improve the performance and versatility of these platforms. Alongside material development, high-resolution 3D printing techniques including SLA, DLP, and FDM will be further refined to achieve reproducible microstructures in both conventional and MAPs. Early integration of hydrogel-based biosensors will allow for real-time monitoring of pH, glucose, and other biomarkers, setting the stage for multifunctional platforms which enable simultaneous sensing and delivery of therapeutics. Preclinical studies in wound healing, TDD, and personal care applications will continue to provide critical data on biocompatibility and mechanical stability, establishing the foundation for clinical translation. In the medium term, the convergence of multidimensional printing technologies, digital health platforms, and AI-assisted design could enable fully personalized epidermal patches capable of real-time physiological monitoring with capabilities such as remotely triggering drug release will enable adaptive therapeutic intervention. In the long term, by bridging advanced materials science, bioprinting technologies, and clinical needs, the next generation of epidermal patches promises to deliver personalized, responsive, and multifunctional solutions that may redefine the standard of care in dermatology and in related fields of clinical practice.

## CRediT authorship contribution statement

**Labiba K. El-Khordagui:** Writing – review & editing, Writing – original draft. **Salma E. El-Habashy:** Writing – original draft. **Abdolreza Simchi:** Writing – review & editing. **Hebat-Allah S. Tohamy:** Writing – original draft. **Maria Letizia Focarete:** Writing – original draft. **Mariangela Rea:** Writing – original draft. **Luana Di Lisa:** Writing – original draft. **Snigdha Roy Barman:** Writing – original draft. **Amit Nain:** Writing – original draft. **Ovidio Catanzano:** Writing – original draft. **Joshua Boateng:** Writing – original draft. **Jagan Mohan Dodda:** Writing – review & editing, Writing – original draft, Conceptualization.

## Data availability statement

The data described in the article are available at https://zenodo.org/uploads/17814839. We would appreciate if other researchers could benefit from our literature and results. This will foster discussions and collaboration among scientists worldwide.

## Ethics approval and consent to participate

This is no ethics approval and consent to participant involved in this article.

## Declaration of competing interest

The authors declare that they have no known competing financial interests or personal relationships that could have appeared to influence the work reported in this paper.
